# A simple open source bioinformatic methodology for initial exploration of GPCR ligands’ agonistic/antagonistic properties

**DOI:** 10.1002/prp2.600

**Published:** 2020-07-13

**Authors:** Athanasios A. Panagiotopoulos, Christina Papachristofi, Konstantina Kalyvianaki, Panagiotis Malamos, Panayiotis A. Theodoropoulos, George Notas, Theodora Calogeropoulou, Elias Castanas, Marilena Kampa

**Affiliations:** ^1^ Laboratory of Experimental Endocrinology School of Medicine University of Crete Heraklion Greece; ^2^ Laboratory of Biochemistry School of Medicine University of Crete Heraklion Greece; ^3^ Institute of Chemical Biology National Hellenic Research Foundation Athens Greece

**Keywords:** agonist, antagonist, biological activity prediction, docking, GPCR, in silico, OXER1

## Abstract

Drug development is an arduous procedure, necessitating testing the interaction of a large number of potential candidates with potential interacting (macro)molecules. Therefore, any method which could provide an initial screening of potential candidate drugs might be of interest for the acceleration of the procedure, by highlighting interesting compounds, prior to in vitro and in vivo validation. In this line, we present a method which may identify potential hits, with agonistic and/or antagonistic properties on GPCR receptors, integrating the knowledge on signaling events triggered by receptor activation (GPCRs binding to G_α,β,γ_ proteins, and activating G_α_, exchanging GDP for GTP, leading to a decreased affinity of the G_α_ for the GPCR). We show that, by integrating GPCR‐ligand and G_α_‐GDP or ‐GTP binding in docking simulation, which correctly predicts crystallographic data, we can discriminate agonists, partial agonists, and antagonists, through a linear function, based on the ΔG (Gibbs‐free energy) of liganded‐GPCR/G_α_‐GDP. We built our model using two G_αs_ (β2‐adrenergic and prostaglandin‐D_2_), four G_αi_ (μ‐opioid, dopamine‐D3, adenosine‐A1, rhodopsin), and one G_αo_ (serotonin) receptors and validated it with a series of ligands on a recently deorphanized G_αi_ receptor (OXER1). This approach could be a valuable tool for initial in silico validation and design of GPRC‐interacting ligands.

**Abbreviations**
5‐oxo‐ETE: 5‐oxo‐6E,8Z,11Z,14Z‐eicosatetraenoic acid
cAMP: Cyclic adenosine monophosphate
GDP: Guanosine diphosphate
GPCR(L): Ligand‐bound GPCR
GPCR: G‐protein‐coupled receptor
GTP: Guanosine‐5'‐triphosphate
Gα: G alpha subunit
OXER1: Oxo‐eicosanoid Receptor 1
pdb: protein data bank
pdbqt: Protein Data Bank, Partial Charge (Q), & Atom Type (T)
RMSD: Root mean square deviation
ΔG: Difference in Gibbs‐free energy

## 1 INTRODUCTION

Progress in biochemistry and cell biology resulted in a better understanding of the events and necessary steps involved in the interaction of a cell with an administered drug substance, leading to the discovery and/or synthesis of novel pharmaceuticals. However, a novel drug development continues to be slow (FDA approved 59 novel drugs in 2018,^1^ including biological factors), as it involves the testing of an increasing number of chemical libraries for positive hits and the subsequent biological validation of promising candidates. Therefore, any progress leading to an initial discrimination of novel potential bioactive compounds could lead to a more accurate identification of possible novel drug candidates.

Of the 59 drugs approved in 2018, 23 target membrane components/receptors, whereas 11 target G‐protein‐coupled receptors (GPCR).^1^ Membrane receptors act as conveyors of extracellular signals into the cell. Membrane receptors can be distinguished as one‐pass single‐chain proteins, acting as mono‐ or oligomers and multiple passes proteins. Among the latter, the seven transmembrane helix (7TM) GPCR family contains ~800 members overall (of which ~400 are olfactory receptors). GPCRs are involved in different signal transduction pathways, triggered by a plurality of extracellular signals (including photons, light‐sensitive compounds, photons, odorants, pheromones, hormones, neurotransmitters, and a number of ligands, varying in size from small molecules to peptides to large (glycol)proteins). GPCR‐initiated signal transduction results in many physiological processes, interfering with the (patho)physiology of many systems, such as the endocrine (including the reproductive), neurological or cardiovascular systems. Such a wide impact, makes GPCR a preferential drug target candidate group.^2^, ^3^, ^4^ Indeed, GPCR‐interacting drugs account for ~34% of the global market share.^3^ However, only a small fraction of GPCRs (206 entries according to https://gpcrdb.org/structure/statistics) have been crystallized to date, making difficult the prediction of novel pharmacological substances.

Molecular docking plays a major role in identifying molecules that might fulfill the requirements of drug development. The applied methodologies simulate the interaction of ligands (small molecules or peptides) with corresponding receptors, in monomeric or oligomeric states. The derived solutions are represented as scoring function (usually reported as the difference in Gibbs‐free energy for molecular association, denoted as ΔG, in kcal/mol, relying on the enthalpy, the entropy and the temperature of the complex), which allows the evaluation of the ligand interaction with the receptor.^5^ In recent years, an increased number of commercial and open source software has been released (see,^6^ for a recent discussion of available resources, and,^7^ for open source solutions). Furthermore, the existence and release of open libraries with chemical structures also accelerated the implementation of this process.^8^

In the field of GPCR pharmacology, the integration of GPCR‐ligand interactions (see, ^9^ for a recent example analysis) resulted in a high success rate of GPCR‐targeting ligands, translated in successful drug design and achieving 78% success rate in Phase I, 39% in Phase II and 29% in Phase III clinical trials ^2^ (see also,^10^ for a successful recent paradigm). However, in spite of the identification of GPCR downstream signaling events (G_α,β,γ_ complex, or arrestin signaling) triggered by receptor activation (see,^11^, ^12^, ^13^ for recent reviews), no attempts have been made to integrate such a knowledge into the search of novel pharmacophores or drugs. Here, we have developed and present a pipeline for GPCR‐ligand interactions and candidate identification, based on free online resources and programs, that also integrates the subsequent steps of molecular docking to GDP‐ and/or GTP‐linked G_α_ protein binding. We show that it can correctly predict small ligand putative agonistic or antagonistic nature, presenting a valuable tool that could significantly accelerate the search of novel molecules in GPCR pharmacology.

## 2 MATERIAL AND METHODS

### 2.1 In silico methods

**Figure 1:**
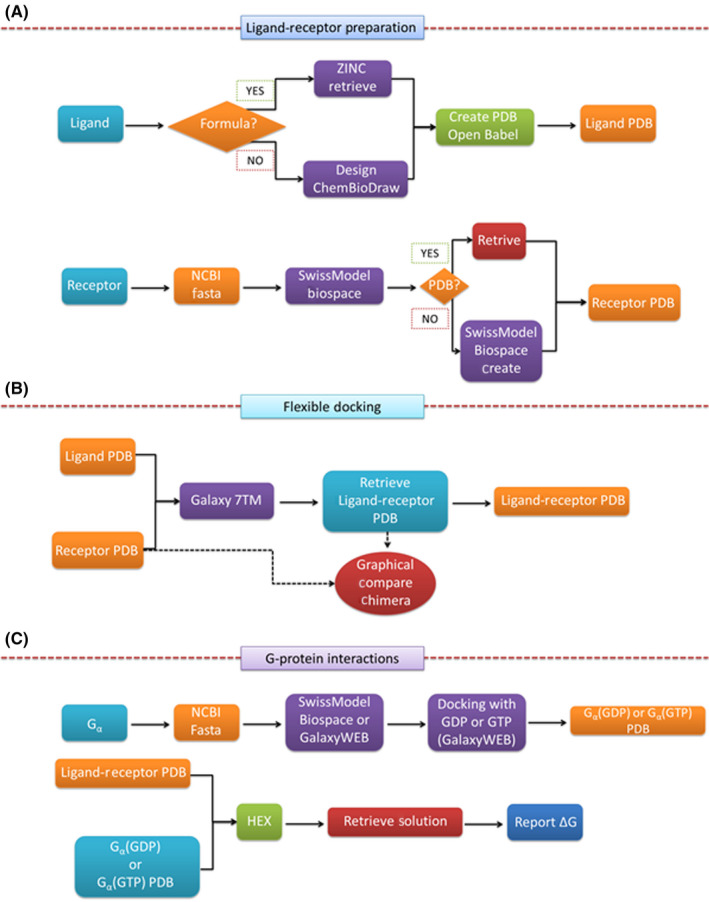
Flowchart of the three steps of the algorithm presented in this paper: (A) Ligand and receptor preparation; (B) Flexible ligand‐receptor binding; (C) G_α_ protein preparation and interaction with liganded receptor. See text and online supporting information 2 for details

Our approach consists of three sequential phases: (A) Ligand and receptor preparation, (B) ligand‐receptor docking and (C) G_α_‐protein interaction (Figure 1 and Supporting Information 2, which provides an illustrated User's Manual).

#### 2.1.1 Ligand and Receptor Preparation

- For receptor preparation: the sequence of human receptors, in fasta format, were retrieved from the NCBI protein database (https://www.ncbi.nlm.nih.gov/protein/) and introduced to the Swiss Model Biospace (http://swissmodel.expasy.org/interactive).^14^
- If a crystalized receptor file (bound or not with an agonist or an antagonist) was available, the system returned the code (and corresponding pdb 3D coordinates file of the receptor or its complex with an agonist or an antagonist, from data stored in the Protein Data Bank (https://www.rcsb.org/).^15^ Whenever the structure contained a ligand, the receptor structure was manually extracted (using a text editor) from the returned pdb file. This did not interfere with the subsequent (flexible) binding, as a full backbone and side chain flexible binding was performed (see below). All protein pdb codes, used in this study, are presented in Table S1.
- If a crystalized structure was not available, the 3D structure of the receptor was simulated by molecular modeling calculations. In this case, the fasta receptor file was introduced in the Swiss Model Biospace (http://swissmodel.expasy.org/interactive),^14^, ^16^ which returned a series of files/crystalized solutions with a variable coverage homology based on the sequence identity to the file in question. We have retained solutions with a coverage homology ≥ 50%‐70%). Please refer to the Swiss Model Biospace for further details of the modeling methodology.
- Known ligands were retrieved from the ZINC database (http://zinc.docking.org/),^17^ usually in a canonical smiles format. Novel molecules were designed in ChemBioDraw (v12.0, Perkin Elmer, Boston, MA, free for Academic use from the University of Cambridge,any other chemical drawing program, such as ChemSketch (https://www.acdlabs.com/resources/freeware/chemsketch/), BKChem (http://bkchem.zirael.org/) or Symyx Draw (https://symyx‐draw.jaleco.com/) can be used at this stage) and the structures were also translated in canonical smiles format. Subsequently, pdb files were created with the Open Babel program (http://openbabel.org).^18^ Ligands (agonists, partial agonists, and antagonists) for each receptor were retrieved from the gene cards web resource (www.GeneCards.org).^19^

#### 2.1.2 Ligand‐receptor docking experiment

Flexible docking algorithms can be broadly divided into methods, in which flexibility is attained during the ligand‐binding interaction (on‐the‐fly methods) and methods applying multiple receptor or ensemble poses, at the beginning or during the simulations (see,^6^ for a discussion). As our goal was to provide a solution, applicable to known or novel GPCRs, in which experimental and/or crystallographic data might not be available, we have opted for on‐the‐fly approach. We have used the online server Galaxy 7TM (galaxy.seoklab.org), in which a full on‐the‐fly ligand and receptor flexibility is implemented.^20^ First, we have used the server for the prediction of the possible binding grooves of each molecule. In our approach, we have restricted our results to only the orthosteric binding site of the molecule (module GalaxySite). Subsequently, we performed a fully flexible binding of the ligand and receptor molecules (module Galaxy7TM). The server uses an algorithm, based on the GalaxyDock2 docking,^21^ which, after an automatic prediction of the ligand binding pocket, permits a full ligand/receptor flexibility during binding simulation. This step is followed by optimization and subsequent refinement, through a specific algorithm named GalaxyRefine,^22^, ^23^ which permits a protein‐ligand structure refinement, by applying iterative side chain repacking and overall structure relaxation,^20^, ^22^, ^23^ returning the pdb files of the best ligand‐receptor solutions. The 3D structures of the liganded and unliganded receptor were compared, using the UCSF Chimera 1.11.2 program,^24^ available from https://www.cgl.ucsf.edu/chimera/. In cases where a crystal was retrieved, the retained solution was compared with the crystal structure. In addition, special attention was paid to confirm that ligands were bound to the orthosteric binding pocket of the corresponding GPCR. Finally, we have compared the correct pose of the ligands, by extracting them from the crystal structure and the retained solution, with a text editor, and compared their RSMD with the Chimera program.

#### 2.1.3 G‐protein interactions

A subsequent step of GPCR‐ligand activation is the binding with G‐proteins, specific sites. More specifically, G_α_ is bound to intracellular activated receptor loops 2 and 3, and G_β,γ_ is bound to intracellular chain 8,^25^, ^26^ initiating specific intracellular signaling events. In this work, we have examined the interaction of GPCRs with G_α_ proteins. It is to note that, after binding of G_α_ protein (bound to GDP and denoted here as G_α_‐GDP), the nucleotide is exchanged, after receptor activation, to GTP (G_α_‐GTP), and the G_α_‐GTP protein is liberated (due to a decrease of affinity for the GPCR) and subsequently triggers specific signaling pathways. Here, we simulated the interaction of known and novel GPCRs with G_α_ proteins.

At a first step, we have retrieved the sequences of G_α_‐proteins, in fasta format, from the NCBI protein database, and after a SwissDock generation of a 3D structure, with known crystal templates, were introduced to the GalaxyWEB to generate and refine the structures, as discussed above for the receptor files. This step was necessary, as the reported crystal structures of the different G_α_ molecules may contain significant gaps. The retrieved structures of G_α_‐proteins were then docked with GDP or GTP in a fully flexible on‐the‐fly method, in the GalaxyWEB server and the liganded G_α_ pdb files were recovered. The same controls, as for the ligand‐GPCR binding were performed for the Gα‐GDP or GTP retained solutions (comparison of the structures by superposition, ligand pose comparison).

The ligand‐receptor and G_α_‐GDP or ‐GTP pdb files were used as input in the Hex 8.0.8 program (http://hex.loria.fr/),^27^ a specialized, locally executed, program, for protein‐protein, or protein‐nucleic acid interactions, based on a spherical rotated protein complexes, taking into account both surface shape and electrostatic charge. Hex returns, through a graphical user interface, a set of > 100 solutions, with the corresponding ΔG values. We have manually inspected and retained only solutions (usually scored first) in which G_α_ molecules bind to GPCRs intracellular loops 2 and 3.

### 2.2 Validation of the obtained solutions

The obtained GPCR‐G_α_ models, obtained from the above procedure, were compared with the reported structures of liganded receptor‐G_α_ proteins. We have retrieved data for the liganded β‐adrenergic receptor (PDB code 3SN6),^28^ the μ‐opioid receptor (PDB code 6DDE),^29^ the rhodopsin receptor (PDB code 6CMO),^30^ the serotonin receptor (PDB code 6G79),^31^ the adenosine A1 receptor (PDB code 6D9H),^32^ co‐crystalized with corresponding Gα proteins. Data were inspected in UCSF Chimera program, by superpositioning of the two structures and the corresponding total and local RMSD value (in Å) were retrieved, with Needleman‐Wunsch alignment ^33^ and with the use of BLOSUM‐62 matrix.^33^

### 2.3 In vitro validation assay

As our goal was to use the proposed algorithm as a prediction tool for the agonistic or antagonistic character of novel ligands on specific GPCRs, we have further validated our in silico results, by exploring the interaction of a series of pregnenolone analogs ^34^ and polyphenol molecules ^35^, as agonists or antagonists of the novel deorphanized GPCR OXER1.^36^, ^37^ OXER1 is an oxo‐eicosanoid receptor, on which 5‐oxo‐ETE is reported to be the physiological agonist and which can also bind other oxo‐eicosanoids, products of arachidonic acid cellular transformation. Recently, we have identified this receptor as a membrane androgen binding site,^38^ with testosterone acting antagonistically on cAMP production and kinases signaling. It is to note that OXER1 binds to a G_αi_ protein and decreases intracellular cAMP production, whereas testosterone, in equimolar concentration, reverts this inhibition by ~50%.^38^ Therefore, in order to validate our in silico data, we have assayed cAMP production in DU145 human prostate cancer cells, bearing OXER1, according to our previous report.^38^

Cells (from Braunschweig, Germany) were cultured in RPMI‐1640 culture medium supplemented with 10% fetal bovine serum (FBS), at 37°C, 5% CO_2_. All media were purchased from Invitrogen (Carlsbad, USA) and all chemicals from Sigma (St. Louis, MO), unless otherwise stated. 5‐oxo‐ETE (5‐Oxo‐(6*E*,8*Z*,11*Z*,14*Z*)‐6,8,11,14‐eicosatetraenoic acid), was from Tocris), Testosterone (Sigma Aldrich), TC150, TC151, and TC153 were synthesized at the Institute of Chemical Biology, National Hellenic Research Foundation, Athens, Greece, B2 and B5 polyphenols were obtained from Professor J. Vercauteren (University of Montpellier, France), whereas Epicatechin was purchased from Sigma Aldrich.

The cyclic adenosine monophosphate (cAMP) production after OXER1 stimulation by 5‐oxo‐ETE (10^‐7^M) alone, or in the presence of testosterone or the other compounds (10^‐6^M, see Results) was examined, with a gain‐of‐signal competitive immunoassay (Promega cAMP Glo TM, Madison, WI). Since OXER1 is a G_αi_‐coupled receptor, forskolin (15 μM) was used to stimulate cAMP production and reveal the inhibitory effect of 5‐oxo‐ETE. The antagonistic effect of testosterone and other agents was assayed as follows (Figure S3): cells were pretreated with the different compounds at a concentration of 10^‐6^M for 15 min at 37°C, prior to the addition of 5‐oxo‐ETE, and cAMP was further assayed. The produced luminescence signal was read in a Microplate Fluorescence Reader (BIO‐TEK Instruments Inc Winooski, Vermont, USA). Results were expressed as % reversion of the 5‐oxo‐ ETE effect, in the presence of forskolin.^38^

### 2.4 Statistical Analysis

Discriminant analysis was performed with the SPSS V21 program (IBM, SPSS Statistics), whereas group comparisons were made with the GraphPad Prism V6.0.5 (GraphPad Software Inc). A statistical threshold of *P* < .05 was retained for significance.

## 3 RESULTS

### 3.1 Implementation of the proposed bioinformatic solution

#### 3.1.1 Training set

##### Ligand‐Receptor interaction

At a first step, we have performed an in silico docking of known small molecules (total number: 78), on six different human GPCRs, crystalized or not, as our method was oriented towards the identification of novel substances for human diseases. We have used the crystalized β_2_‐adrenergic (pdb 3SN6 ^28^), dopamine D_3 _(pdb 3PBL ^39^), μ‐opioid (pdb 6DDE ^29^), adenosine A_1_ (pdb 6D9H ^32^), and rhodopsin (pdb 6CMO ^30^) receptors. For prostaglandin DR_2_ receptors, as a crystal was not available, our model was based on the human C_5a_ anaphylatoxin chemotactic receptor 1, pdb 5O9H ^40^ (see Material and Methods for details). The receptor and ligand molecules (in pdb format) were then introduced in the Galaxy 7TM server, and a fully flexible (ligand and receptor) binding was performed.

Results (as changes of the Gibbs‐free energy changes, ΔG, in kcal/mol) are shown in Tables 1, 2, 3, column GPCR‐Ligand.

**Table 1:** Fully flexible ligand binding results on β_2_‐adrenergic (pdb 3SN6) and prostaglandin DR_2_ (based on pdb 5O9H) receptors, together with the liganded GPCR (GPCR(L)) binding to G_αs_ in its GDP and GTP‐bound forms. All data are reported as differences in the Gibbs‐free energy (ΔG), expressed in kcal/mol. The effect column presents the reported action of the compound (bibliography) and the predicted effect by the proposed model (Model). See text for details

RECEPTOR	LIGAND	GPCR‐Ligand (kcal/mol)	GPCR(L)‐G_α_GDP (kcal/mol)	GPCR(L)‐G_α_GTP (kcal/mol)	EFFECT Bibliography/Model
β_2_‐adrenergic	Bitolterol	−16.7	−910.6	−700.1	Ago/Ago
	Formoterol	−13.4	−877.5	−700.9	Ago/Ago
	Isoprenaline	−10.8	−933	−699	Ago/Ago
	Levosalbutamol	−12	−890.7	−695	Ago/Ago
	Orciprenaline	−11.1	−892.3	−696	Ago/Ago
	Ritodrine	−11.8	−924.4	−704.4	Ago/Ago
	Salbutamol	−11.4	−961.1	−691.3	Ago/Ago
	Salmeterol	−16.8	−956.9	−695.2	Ago/Ago
	Terbutaline	−11.2	−853.1	−697	Ago/Ago
	ICI118,551	−10.6	−725.1	−707.5	Antago/PA
	Butoxamine	−12.1	−706.4	−696.6	Antago/PA
	Propranolol	−11.6	−719.7	−706.6	Antago/PA
	BI‐167107	−15.4	−971.3	−705.6	Ago/Ago
Prostaglandin DR_2_	Prostaglandin‐E_2_	−14.3	−1079	−475.6	Ago/Ago
	Prostaglandin‐F_2a_	−14.7	−1091.9	−431.3	Ago/Ago
	Prostacyclin	−14.6	−1003.2	−455	Ago/Ago
	Fevipiprant	−14.9	−462.2	−412.2	Antago/Antago
	Ramatroban	−15.4	−422.1	−416.4	Antago/Antago
	Setipiprant	−14.9	−442.8	−418	Antago/Antago

Abbreviations: Ago, Agonist; Antago, Antagonist; GPCR(L), Ligand‐bound GPCR; PA, Partial Agonist.

**Table 2:** Fully flexible ligand binding results on dopamine D_3_ (pdb 3PBL), μ‐opioid (pdb 5C1M), Adenosine A_1_ (pdb 6D9H), and Rhodopsin (pdb 6CMO) receptors, together with the liganded GPCR (GPCR(L)) binding to G_αi_ in its GDP and GTP‐bound forms. All data are reported as differences in the Gibbs‐free energy (ΔG), expressed in kcal/mol. The effect column presents the reported action of the compound (bibliography) and the predicted effect by the proposed model (Model). See text for details

RECEPTOR	LIGAND	GPCR‐Ligand (kcal/mol)	GPCR(L)‐G_α_GDP (kcal/mol)	GPCR(L)‐G_α_GTP (kcal/mol)	EFFECT Bibliography/Model
Dopamine D_3_	Dopamine	−9.6	−779.9	−349	Ago/PA
	Quinpirole	−9.8	−719.7	−378.8	Ago/PA
	5OH‐DPAT	−8.8	−864	−348.4	Ago/Ago
	Pergolide	−9.4	−780.2	−400.7	Ago/PA
	Captodiame	−10	−793	−311	Ago/PA
	Apomorphine	−6.7	−804.9	−376.3	Ago/Ago
	Aripiprazole	−16.9	−761.1	−327.7	PA/PA
	Cariprazine	−11.3	−763.6	−388.8	PA/PA
	Buspirone	−11.1	−772.3	−334.5	PA/PA
	Pardoprunox	−5.2	−787.1	−349.4	PA/PA
	Nafadotride	−8.5	−652.5	–373.3	Antago/Antago
	Raclopride	−10.7	−685.4	−330	Antago/Antago
	Haloperidol	−10.5	−683	−321.6	Antago/Antago
	Amisulpride	−10.2	−686	−396.5	Antago/Antago
	Cyproheptadine	−6.1	−690.8	−285.7	Antago/Antago
	Risperidone	−12.1	−682.7	−370.7	Antago/Antago
	Acetylmorphone	−9.6	−855.2	−615.4	Ago/Ago
	Benzhydrocodone	−10.9	−821.3	−616.9	Ago/Ago
μ‐opioid	Heroin	−12.4	−1045.4	−620.7	Ago/Ago
	Methadone	−8.7	−902.4	−561.6	Ago/Ago
	Nicocodeine	−12.0	−855.1	−526.4	Ago/Ago
	Butorphanol	−10.2	−769.8	−540.4	PA/PA
	Ciprefadol	−9.0	−773.2	−656.4	PA/PA
	Cyclorphan	−8.9	−779.8	−669.2	PA/PA
	Ketorfanol	−9.0	−778.6	−638.6	PA/PA
	Xorphanol	−9.0	−783.3	−674.4	PA/PA
	Moxazocine	−8.5	−799.0	−629.7	PA/PA
	Nalbuphine	−10.1	−780.7	−589.9	PA/PA
	Nalmefene	−9.8	−697.2	−650.7	Antago/Antago
	Nalodeine	−9.7	−703.1	−594.9	Antago/Antago
	Nalorphine	−10.7	−699.8	−651.2	Antago/Antago
	Naloxone	−9.3	−698.4	−575.7	Antago/Antago
	Naltrexone	−9.9	−691.3	−619.2	Antago/Antago
	Levallorphan	−8.8	−698.4	−584.0	Antago/Antago
	DAMGO	−16.3	−861.0	−582.0	Ago/Ago
Adenosine A_1_	ADO	−8.7	−1079.6	−646.1	Ago/Ago
	CCPA	−12.5	−1062.6	−659.9	Ago/Ago
	CPA	−11.9	−1009.9	−563.6	Ago/Ago
	N(6)‐Cyclohexyladenosine	−11.4	−1099.7	−679.7	Ago/Ago
	Tecadenoson	−11.9	−888.4	−585.8	Ago/Ago
	Selodenoson	−13	−990.8	−638.6	Ago/Ago
	Caffeine	−6.3	−631.4	−372.6	Antago/Antago
	Bamifylline	−12.1	−674.2	−553.3	Antago/Antago
	CGS‐15943	−9.2	−531.9	−356.2	Antago/Antago
	Theophylline	−6.1	−547.1	−469	Antago/Antago
Rhodopsin	Retinal	−9.9	−972.9	−359.2	Ago/Antago
	Halothane	−4.2	−377.7	−335.9	Antago/Antago
	Palmitic Acid	−9.9	−447.7	−356.6	Antago/Antago
	Zoledronic Acid	−10.7	−442.1	−326.3	Antago/Antago

Abbreviations: Ago, Agonist; Antago, Antagonist; GPCR(L), Ligand‐bound GPCR; PA, Partial Agonist.

**Table 3:** Fully flexible ligand binding results on the serotonin receptor (pdb 6G79), with the liganded GPCR (GPCR(L)) binding to G_αo_ in its GDP and GTP‐bound forms. All data are reported as differences in the Gibbs‐free energy (ΔG), expressed in kcal/mol. The effect column presents the reported action of the compound (bibliography) and the predicted effect by the proposed model (Model). See text for details

RECEPTOR	LIGAND	GPCR‐Ligand (kcal/mol)	GPCR(L)‐G_α_GDP (kcal/mol)	GPCR(L)‐G_α_GTP (kcal/mol)	EFFECT Bibliography/Model
Serotonin	Ergotamine	−17.5	−1193.5	−368.5	Ago/Ago
	Oxymetazoline	−9.9	−830.2	−369.7	Ago/Ago
	Sumatriptan	−9.4	−993.4	−320.8	Ago/Ago
	Zolmitriptan	−10.5	−886.7	−398	Ago/Ago
	Dextromethorphan	−11.1	−713.8	−376.4	PA/PA
	Ziprasidone	−9.1	−692.5	−338	PA/Antago
	Asenapine	−8.9	−736.9	−380.2	PA/PA
	Vortioxetine	−9.9	−701.9	−356.6	PA/Antago
	Metitepine	−10.4	−618	−362.7	Antago/Antago
	Yohimbine	−10.2	−588.9	−312.2	Antago/Antago
	Metergoline	−12.2	−542.6	−302.9	Antago/Antago
	Isamoltane	−9.9	−523.3	−325.6	Antago/Antago

Abbreviations: Ago, Agonist; Antago, Antagonist; GPCR(L), Ligand‐bound GPCR; PA, Partial Agonist.

##### Interaction of the ligand‐receptor complex with G_α_‐proteins

A subsequent step following ligand‐GPCR interaction is the binding of the liganded receptor to the heteroprotein complexG_α,β,γ_,^11^, ^12^, ^13^ triggering specific signaling events. G_α_ proteins interact with intracellular loops 2 and 3, whereas G_β,γ_ with intracellular loop 3 and the intracellular C‐terminal helix 8.^25^ G_α_ proteins are bound to guanine nucleotides; specifically, G_α_ proteins are bound to GDP, and in this form, they interact with the ligand‐activated GPCR (GPCR(L)). The affinity of the GPCR(L)‐G_α_ decreases substantially when an exchange of GDP by GTP occurs, leading to G_α_ dissociation from the GPCR(L) complex and the initiation of intracellular signaling events.^25^

**Figure 2:**
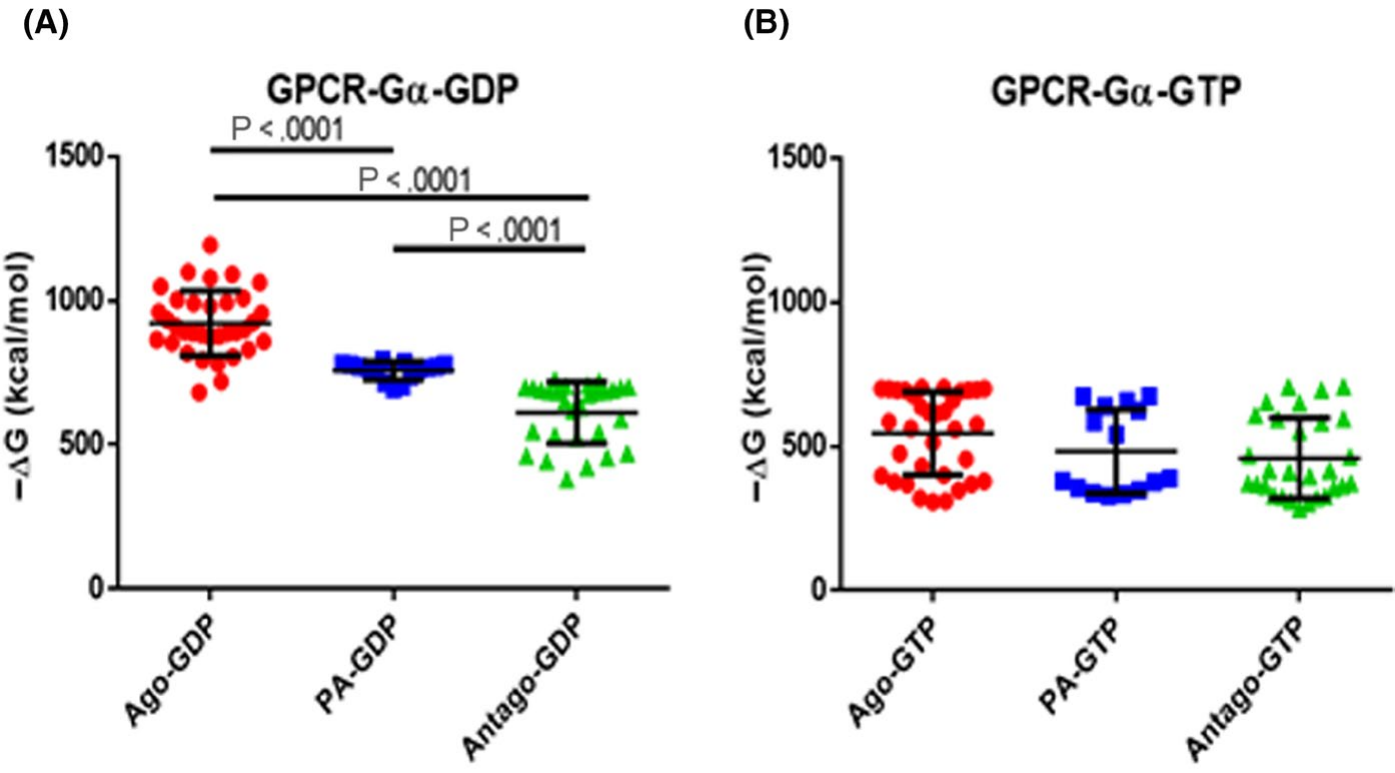
ΔG GPCR(L)‐G_α_ values (presented in Tables 1, 2, 3), upon agonist, partial agonist (PA) and antagonist binding. In (A), the negative ΔG GPCR(L)‐G_α_GDP value is shown, whereas in (B) the corresponding negative ΔG GPCR(L)‐G_α_GTP value is depicted. Post hoc group comparisons were made after ANOVA, with the Turkey's multiple comparison test, in GraphPad Prism V6

Interaction of the liganded receptor with G_α_‐GDP and G_α_‐GTP complexes is also shown in Tables 1, 2, 3. We have used G_αs_‐, G_ao_‐, or G_αi_‐ molecules according to the reported physiological interaction of each receptor with G_α_ proteins. In this case, as the two interacting structures are macromolecules, the resulting ΔG values were much higher.^41^ When GDP was exchanged for GTP on the G_α_ proteins (Tables 1‐3), the interaction of G_α_ proteins with the receptor was significantly decreased. In this case, the obtained values do not differ among agonists, partial agonists, and antagonists (Figure 2).

##### Verification of the GPCR‐ligand binding and G_α_ interaction

In view of the potential application of the proposed tool for the identification of possible small molecule agonistic and antagonistic candidates for GPCRs, we have performed a number of verifications of our approach:

**First,** we simulated the interaction of unliganded GPCRs with either nonliganded G_αs/i/o_ proteins or G_αs/i/o_ proteins bound to either GDP or GTP. The same test was performed with liganded GPCRs (Table S2). We show that unliganded GPCRs interact with a substantially lower affinity, or do not interact at all, with either nonliganded or GDP/GTP‐bound G_α_ proteins. In addition, we show that liganded GPCRs do not interact with nonliganded G_α__s/i/o_ proteins. This result confirms the validity of our approach, which is in line with the physiological function concerning the initiation of signaling by GPCRs.^11^, ^12^, ^13^

**Second,** we have compared the retained solutions of the ligand receptor complexes with those deposited in the PDB database. In all cases, a very small RMSD was found between the simulated solution and the crystal structure (Specific examples are presented in Figure S1), suggesting the very good match of our simulation with crystallography‐obtained data.

**Third,** we have compared (whenever possible) the simulated structure of the complex [GPCR‐L]‐[G_α_‐GDP] with available crystals (human β‐adrenergic (pdb 3SN6),^28^ the μ‐opioid (pdb 6DDE),^29^ the rhodopsin (pdb 6CMO),^30^ the serotonin (pdb 6G79),^31^ and the adenosine A_1_ receptor (pdb 6D9H),^32^ co‐crystalized with the corresponding G_α_ proteins) (Figure S2). We report that, a very close match for all solutions, with the exception of the rhodopsin receptor (RMSD 15.4 Å). In the latter, a very good match was found at the interacting part of the G_α_ protein, whereas the observed differences in G_α_ proteins might be attributed to the recalculation of these proteins’ 3D structure, due to major disruptions of the protein structures in the G_α_ crystal.

**Finally**, we have tried to simulate G_α_‐GDP binding to an unrelated multipass membrane protein (aquaporin monomer, extracted from pdb 6KXW). All three G_α_‐GDP complexes did not interact with this protein monomer, corroborating about the specificity of the simulated interaction.

In view of the above, we have concluded that our approach may indeed correctly simulate the interaction of known GPCRs with their corresponding G_α_‐proteins.

##### G_α_ binding to the liganded receptor can discriminate between GPCR agonists and antagonists

After determining the different ΔGs for ligand‐GPCR binding and for liganded GPCR‐liganded G_α_ interaction (presented in Tables 1, 2, 3), we explored whether these data could be used for the prediction of agonistic or antagonistic properties of the different ligands. A backward elimination discriminant analysis, with the 78 compounds presented here and their reported agonistic, antagonistic, or partial agonistic properties retained only ΔG of the liganded GPCR‐GDP‐bound G_α_, protein as a significant discriminant element. A linear function of this factor (0.010 x ΔG GPCR(L)‐G_α_GDP + 7.895) was sufficient to correctly discriminate 93% of antagonists, 87% of partial agonists, and 88% of agonists (F = 84.089, *P* = 4.31^‐20^). Using group centroids, we have estimated the cut‐offs of the three groups (agonists, partial agonists, and antagonists), through a weighted mean calculation (Weighted Mean=((Mean_1_xN_1_)+(Mean_2_xN_2_))/(N_1_ + N_2_), where N is the number of substances used for the calculation). A cut‐off of 1.231 (corresponding to a ΔG GPCR(L)‐G_α_GDP of −666 kcal/mol) between antagonists and partial agonists and a cut‐off of −0.978 (corresponding to a ΔG GPCR(L)‐G_α_GDP of −887 kcal/mol) between partial agonists and full agonists was calculated. This prediction is reported in the last column of Tables 1‐3. As shown, in the majority of cases (70/80, 87.5%) a correct classification was obtained; however, in 8/80 cases, reported action and prediction were not obtained. Inspection of the chemical structures of misclassified substances did not provide a valid clue about this misclassification. However, in the majority of cases, these misclassified compounds have a receptor‐Gα value near the cut‐off of the different categories. We presume that, with a better calculation of the classification intervals with a larger number of compounds and/or GPCRs, this 12.5% mis‐classification might improve.

### 3.2 Validation set

#### 3.2.1 Classification of novel compounds

At a first step, we have retrieved, from the list of FDA‐approved drugs for 2017 and 2018,^1^, ^42^ four compounds, characterized as agonists or antagonists of GPCRs (Prucalopride as a selective 5HT4 receptor, Lofexidine as an agonist of α2A adrenergic receptor, Latanoprostene as a selective agonist of PgF receptor, and Naldemedine as a μ‐opioiod receptor antagonist). We have applied our method, in order to provide agonistic or antagonistic properties of the compounds (Table 4). We have verified whether these drugs could interact with the receptor we have analyzed in this work. Surprisingly, all compounds interact with other GPCR subtypes, with a relative high affinity, docked to the correct ligand binding pocket of each molecule. However, neither Prucalopride, nor Lofexidine or Latanoprostene induce a binding of the corresponding G_α_ protein. In contrast, Naldemedine induces a G_αi_ binding, with an affinity of −657 kcal/mol, correctly classifying it as an opioid receptor antagonist.

**Table 4:** Simulation data of four novel compounds approved by the FDA in 2018 (1). For each compound its affinity (Galaxy Docking) and its interaction with the corresponding G_α_ protein are shown. The interaction of each drug with the GPCR analyzed here and with its cognate receptor (for which an FDA approval was provided) is shown. X denotes nonassociation

Ligand	Receptor	Galaxy Docking	HEX Docking with G_α_GDP	HEX Docking with G_α_GTP	Comment
Prucalopride	5‐HT_1B_	−12.323	X	X	Selective Agonist of 5‐HT_4_ Receptor
	5‐HT_4_ (G_αs_)	−13.566	−1065.67	−690.85	
Lofexidine	α_B2_‐Adrenergic	−10.619	X	X	Selective Agonist of α_2Α_‐Adrenergic Receptor
	α_2Α_‐Adrenergic (G_αi_)	−8.933	−1061.31	−473.26	
Latanoprostene	PTGDR2	−20.039	X	X	Selective Agonist of Prostagladine F Receptor
	Prostagladine F‐R (G_αq_)	−19.614	−1029.24	−551.30	
Naldemedine	μ‐Opioid	−15.576	−657.09	−594.69	Opioid Receptor Antagonist

At a second step, we have investigated the interaction of Prucalopride, Lofexidine, and Latanoprostene with 5HT4 receptor, α2A adrenergic and PgF receptor, respectively. Neither of these three human receptors have been crystalized yet. We have therefore used the Swiss Model Biospace ^14^ to provide the most promising solution of its 3D structure for each receptor, by introducing each receptor sequence in fasta format. The best returned solutions were based on crystals 6AK3 (prostaglandin E receptor) for PgF receptor, crystal 5V54 (5‐HT1B receptor) for the α2Α‐Adrenergic Receptor, and crystal 3PDS (B_2 _adrenoreceptor) for 5‐HT_4_. These models have further been refined (and completed whenever necessary) in the Galaxy Refine routine of the Galaxy Web server, and binding of the corresponding compounds was performed, followed by the binding of the GDP‐ or GTP‐bound corresponding G_α_ protein (the protein used is denoted in parentheses in the first column of Table 4). Applying our cut‐off values −666 and −887 kcal/mol for GDP‐bound Gα protein, we show that we have correctly identified the three agonistic drugs.

#### 3.2.2 Detection of agonists and antagonists for a novel receptor (OXER1)

A valid prediction method should provide useful hints about the agonistic or antagonistic properties of both crystalized or not GPCRs. Hence, we used our approach to predict the agonistic‐antagonistic properties of a number of substances, of very different molecular structure (lipids, steroids, polyphenols), on the oxo‐eicosanoid receptor OXER1.

OXER1 was deorphanized in 2002‐3 and was found to be the endogenous receptor for the arachidonic acid metabolic product 5‐oxo‐ETE, produced through the action of 5‐lipoxygenase (5‐LOX) and peroxidase.^36^, ^37^ However, recently, we have reported that OXER1, coupled to G_αi_ protein, also mediates membrane‐initiated androgen actions (see,^38^ and references herein), with testosterone acting as an antagonist. As OXER1 has not been crystallized yet, we have used the Swiss Model Biospace ^14^ to provide the most promising solution of its 3D structure and retained a solution, based on P2Y purine receptor, for docking simulations.^38^

G_αi_‐GDP bound to the 5‐oxo‐ETE (agonist)‐ or testosterone (antagonist)‐ OXER1 complex, with a ΔG GPCR(L)‐G_α_GDP of −836 and −663 kcal/mol, respectively (Table 5). We have also calculated the affinity of a series of derivatives of arachidonic acid biotransformation, which have been previously reported to act as partial OXER1 agonists (see https://genecards.weizmann.ac.il/v3/cgi‐bin/carddisp.pl?gene=OXER1 and references therein). Obtained ΔG GPCR(L)‐G_α_GDP values are intermediate between 5‐oxo‐ETE and testosterone, verifying their partial agonistic nature (Table  5).

**Table 5:** Fully flexible ligand binding results on the OXER1 receptor, together with the liganded GPCR (GPCR(L)) binding to G_αi_ in its GDP‐ and GTP‐bound forms. All data are reported as differences in the Gibbs‐free energy (ΔG), expressed in kcal/mol. Data from 5‐HETE, 12‐HpETE, 15‐HpETE, 12‐HETE, and 15‐HETE were from previous studies, and extracted from the Gene Cards web site, whereas data for all other compounds were experimentally verified, through an inhibition of 5‐oxo‐ETE effect on cAMP production. Here, the maximum inhibition of forskolin stimulated inhibition of cAMP production by 1 μM 5‐oxo‐ETE (the natural ligand of OXER1 receptor) was set as 100% inhibition, and data obtained by all other compounds were compared to this maximum value at a similar 1 μM concentration, added simultaneously with 5‐oxo‐ETE. Please refer to the Material and Methods section, to Figure 3E and text of reference (38), and to Figure S3 for further details. The effect column presents the reported action of the compound (bibliography), the experimental validation (experimental), and the predicted effect by the proposed model (Model). See text for further details

RECEPTOR	LIGAND	GPCR‐Ligand (kcal/mol)	GPCR(L)‐G_α_GDP (kcal/mol)	GPCR(L)‐G_α_GTP (kcal/mol)	% cAMP INHIBITION (Experimental data^a^)	EFFECT Bibliography/ (Experimental)/ Model
OXER1	5‐oxo‐ETE	−14.5	−896.5	−528.3	100	Ago/(Ago)/Ago
	Testosterone	−10.9	−663	−507.9	51 ± 2.55	Antago/(Antago)/Antago
	5‐HETE	−14.5	−710.8	−565.5	NA	PA/PA
	12‐HpETE	−14.2	−713.8	−521.4	NA	PA/PA
	15‐HpETE	−14.1	−758.3	−544	NA	PA/PA
	12‐HETE	−14.5	−717.7	−566.3	NA	PA/PA
	15‐HETE	−13.3	−723.8	−566.6	NA	PA/PA
	TC150	−15.4	−657.3	−544.2	48 ± 1.92	NA/(Antago)/Antago
	TC151	−16.1	−645.2	−541.1	66 ± 4.81	NA/(Antago)/Antago
	TC153	−14.5	−635.2	−572.6	54 ± 3.23	NA/(Antago)/Antago
	B2	−25.4	−665.2	−485.1	48 ± 3.47	NA/(Antago)/Antago
	B5	−25.1	−736.2	−590.4	25 ± 5.31	NA/(PA)/PA
	Epicatechin	−13	−642.8	−552.5	67 ± 2.98	NA/(Antago)/Antago

Abbreviations: Ago, Agonist; Antago, Antagonist; GPCR(L), Ligand‐bound GPCR; NA, Non‐available; PA, Partial Agonist.

^a^ Mean ± SE, n = 3.

In addition to the above compounds, we have tested a series of pregnenolone analogs, with reported antiproliferative activity in different cancer cell lines.^34^ As shown in Table 5, docking simulations revealed that TC150, TC151, and TC153 bind to OXER1 (they interact with the same binding grove as 5‐oxo‐ETE and testosterone, not shown) and the ligand‐receptor complex bound G_αi_ with a ΔG GPCR(L)‐G_α_GDP −657, −645, and −635 kcal/mol, respectively, pointing out an antagonistic nature, compatible with that of testosterone. Finally, a series of polyphenols (epicatechin and its dimers B2 and B5), which we have previously reported as mimicking membrane testosterone actions ^35^ showed ΔG GPCR(L)‐G_α_GDP values −642, −665, and −736 kcal/mol, respectively, identifying them as antagonists (epicatechin, B2) or partial agonist (B5).

In order to verify our prediction, we have experimentally tested whether these compounds can antagonize 5‐oxo‐ETE action on cAMP production, like testosterone^38^ OXER1‐G_αi_ interaction results in an inhibition of cAMP.^36^, ^37^, ^38^ This is experimentally tested by stimulating cAMP production in cells by forskolin and detecting the cAMP inhibition after incubation of cells with the corresponding ligands. We have previously shown that testosterone incubation of prostate cancer cells reverts the 5‐oxo‐ETE‐induced inhibition, in a dose‐dependent manner.^38^ Here, we have applied the same protocol using pregnenolone analogs and polyphenols, after forskolin stimulation of DU145 human prostate cancer cells and application of 5‐oxo‐ETE. Table 5 presents the normalized cAMP inhibition (5‐oxo‐ETE inhibition = 100%). Testosterone reverts this inhibition by 51%, at a concentration 1 μM, as reported previously.^38^ Of the tested compounds, all reverted 5‐oxo‐ETE cAMP inhibition by 48%‐67%, at the same 1 μM concentration, classifying them as antagonists of OXER1, with the notable exception of B5 procyanidin, which reverted 5‐oxo‐ETE cAMP inhibition by only 25%, classifying it as a partial agonist, as also suggested by the in silico binding data.

## 4 DISCUSSION

Drug development is a laborious procedure, necessitating the testing the interaction of a large number of potential candidates, with potential target (macro) molecules. Therefore, any method which could provide an initial screening of chemicals as positive hits, might be of interest for the selection of interesting compounds, which could decrease the time‐frame in drug discovery, prior to in vitro and in vivo validation. Here, we report a method (see Figure 1 for a schematic representation) which may be used for the initial, in silico screening of potentially active compounds, taking into account the binding of the ligand on the corresponding receptor and its subsequent simulated affinity for G_α_‐GDP. We report that the latter may correctly discriminate ~90% of substances between agonists, partial agonists and antagonists.

GPCRs‐related drugs account for 34% of all drug targets.^2^, ^3^, ^43^ In addition, several pharmacological substances, designed to interact with a single target, were found to mediate effects via several GPCRs, exhibiting a specific polypharmacological profile (see,^12^ for a discussion). However, the crystal structures of only 62 unliganded GPCRs are available today, and 206 in combination with different agonistic or antagonistic small molecules (https://gpcrdb.org/structure/statistics), whereas almost 100,000 distinct putative GPCR ligands have been reported in ChEMBL,^44^of them, biological activity has been reported only for only 3%. Our in silico approach, based on publicly available programs and web resources, may be used as an initial pipeline for the identification of compounds to be further tested as putative drug candidates. This was further verified here, with a noncrystalized GPCR (OXER1), on which, our pipeline correctly identified agonists and antagonists.

The novelty of our approach relies on exploiting, in addition to ligand‐GPCR fully flexible docking, an initial step of the subsequent signaling event, their interaction with G_α_‐proteins,^25^ to provide a quick initial estimate of ligand agonistic or antagonistic properties. In our analysis, agonistic ligands induce a significantly higher affinity for the liganded receptor G_α_‐GDP interaction. This affinity decreases substantially when the same G‐protein is bound to GTP, expressing the biologically relevant dissociation of the GTP‐bound G‐protein from the receptor and the initiation of intracellular signaling events.^25^ Our approach is based on bibliographic data from known ligand interactors of crystalized or noncrystalized G_αs_, G_αo_, or G_αi_‐interacting receptors. The obtained solutions were compatible with biological data and correctly predict the full or partially agonistic and antagonistic properties of the ligands. Furthermore, the obtained solutions of the liganded receptor‐GDP/GTP bound G_α_ heteroprotein complexes do not differ significantly from the corresponding crystal structures, whenever available. However, in its current form, the proposed approach has some drawbacks (not fully automated, necessitating human intervention for the selection of the G‐protein‐receptor binding solution and not taking into account GPCR‐β‐arrestin, G_β,γ_ or allosteric binding). In addition, the proposed cut‐offs may be refined with the addition of additional GPCRs and ligands, or modified if other simulation programs are used for the calculation of GPCR‐L and G_α_‐GDP affinities.

## 5 CONCLUSION

Our data clearly show that, by integrating sequential steps of receptor downstream signaling in ligand‐GPCR simulations, as expressed by GDP‐G_α_ binding, we can correctly predict the nature (agonist, antagonist, partial agonist) of a given small molecule. This approach, combined to properly implemented and successfully validated QSAR methods,^45^ may represent a useful addition to current research processes for the initial prediction and design of novel GPRC‐interacting molecules. It might be of interest to explore further whether similar initial estimates might be also applied on other, non‐GPCR, receptors, which could provide a generalization of our approach.

## DISCLOSURES

None declared.

## AUTHORS’ CONTRIBUTIONS

MK and EC conceived and designed the study, and wrote the paper. AP, CP, KK, and PM performed the analyses and experiments. AP, TC, GN, and PAT participated in its design and coordination and helped to draft the manuscript. All authors read and approved the final manuscript.

## ETHICAL STATEMENT

This article does not contain any studies involving animals or human participants performed by any of the authors.

## Supporting information

Supplementary Material

## Data Availability

The data that support the findings of this study are available from the corresponding author upon reasonable request. Some data may not be made available because of privacy or ethical restrictions.
