# Supplementary material for: A simple open source bioinformatic methodology for initial exploration of GPCR ligands’ agonistic/antagonistic properties
Source: Pharmacol Res Perspect. 2020 Jul 13;8(4):e00600. doi: 10.1002/prp2.600 (PMC7358596; doi:10.1002/prp2.600)
Supplement: Supplementary file 1 — Supplementary Material [file PRP2-8-e00600-s001.pdf]

Supporting Information

A simple open source bio-informatic methodology for initial exploration of GPCR ligands' agonistic/antagonistic properties

Athanasios A. Panagiotopoulos[1], Christina Papachristofi[1], Konstantina Kalyvianaki[1], Panagiotis Malamos[1], Panayiotis A. Theodoropoulos[2], George Notas[1], Theodora Calogeropoulou[3], Elias Castanas[1,*], Marilena Kampa[1,*]

Affiliations

1. Laboratory of Experimental Endocrinology, University of Crete, School of Medicine, Heraklion, 71013 Greece
2. Laboratory of Biochemistry, University of Crete, School of Medicine, Heraklion, 71013 Greece
3. Institute of Biology, Medicinal Chemistry and Biotechnology, National Hellenic Research Foundation, Athens, 11635, Greece

*** Corresponding authors**

Marilena Kampa (kampam@uoc.gr), and Elias Castanas (castanas@uoc.gr)

Table of Contents

Supporting Information 1 …………………………………………………………………………… 3

Supplemental Tables.. 3

Supplemental Table 1.. 3

Supplemental Table 2.. 4

Supplemental Figures.. 5

Supplemental Figure 1 5

Supplemental Figure 2 10

Supplemental Figure 3 11

References... 12

Supporting Information 2 …………………………………………………………………...…… 13

Concise Manual of the pipeline used for *in silico* drug screening……………………………….13

Supporting Information 1

Supplemental Tables

Supplemental Table 1

Table presents the PDB codes for the proteins utilized in molecular docking experiments. The nature of the co-crystalized ligand is also presented. “-“ indicates that the receptor crystal does not contain any ligand.

**Protein**	**PDB ID Code**	**Ligand**
$G_{\alpha s}$-bound β_2-adrenergic Receptor	3SN6	Agonist
C5a anaphylatoxin chemotactic Receptor 1	5O9H	Antagonist
$G_{\alpha i}$-bound dopamine D3 Receptor	3PBL	Antagonist
μ-opioid Receptor	6DDE	Agonist
Adenosine A1 Receptor	6D9H	Agonist
Rhodopsin Receptor	6CMO	Agonist
$G_{\alpha o}$-coupled serotonin Receptor	6G79	Agonist
$G_{\alpha s}$ Protein	3SN6	GTP
$G_{\alpha i}$ Protein	6DDE	-
$G_{\alpha o}$ Protein	6G79	-

Supplemental Table 2

Table presents the interaction of unliganded and liganded GPCRs with a $G_{\alpha s}$ protein-interacting β2-adrenergic and PTGDR2 receptor, $G_{\alpha i}$ protein-interacting Dopamine D_3, μ-Opioid, adenosine A1 and rhodopsin receptor and $G_{\alpha o}$ protein-interacting serotonin 1B receptor. In each case, interaction of the free or nucleotide (GDP or GTP) -bound corresponding G_{α} protein is reported. All data are reported as differences in the Gibbs free energy (ΔG), expressed in kcal/mol. X denotes that no interaction between the GPCR and the corresponding Gα protein was found.

**Receptor**	**GPCR - non-liganded Gα protein (Kcal/mol)**	**GPCR - Gα-GDP (Kcal/mol)**	**GPCR - Gα-GTP (Kcal/mol)**
β2-adrenergic receptor-(unliganded)	X	X	X
β2-adrenergic receptor-(Isoprenaline)	X	-933.0	-699.0
PTGDR2 receptor-(unliganded)	-357.6	X	X
PTGDR2 receptor-(Prostaglandin-E2)	X	-1080.0	-475.6
Dopaminergic receptor -(unliganded)	-281.7	-378.2	-288.8
Dopaminergic receptor -(Dopamine)	X	-779.9	-349.0
μ-Opioid receptor-(unliganded)	-254.5	-258.5	X
μ-Opioid receptor-(Heroin)	X	-1049.6	-621.5
Adenosine A1 receptor-(unliganded)	X	X	X
Adenosine A1 receptor (CCPA)	X	-1062.6	-659.9
Rhodopsin receptor (unliganded)	-445.2	X	X
Rhodopsin receptor (Retinal)	X	-682.1	-308.7
5-Hydroxytryptamine (serotonin) receptor 1B (5-HT1B) -(unliganded)	X	X	X
5-Hydroxytryptamine (serotonin) receptor 1B (5-HT1B) -(Ergotamine)	-354.2	-1193.5	-368.5

Supplemental Figures

Supplemental Figure 1

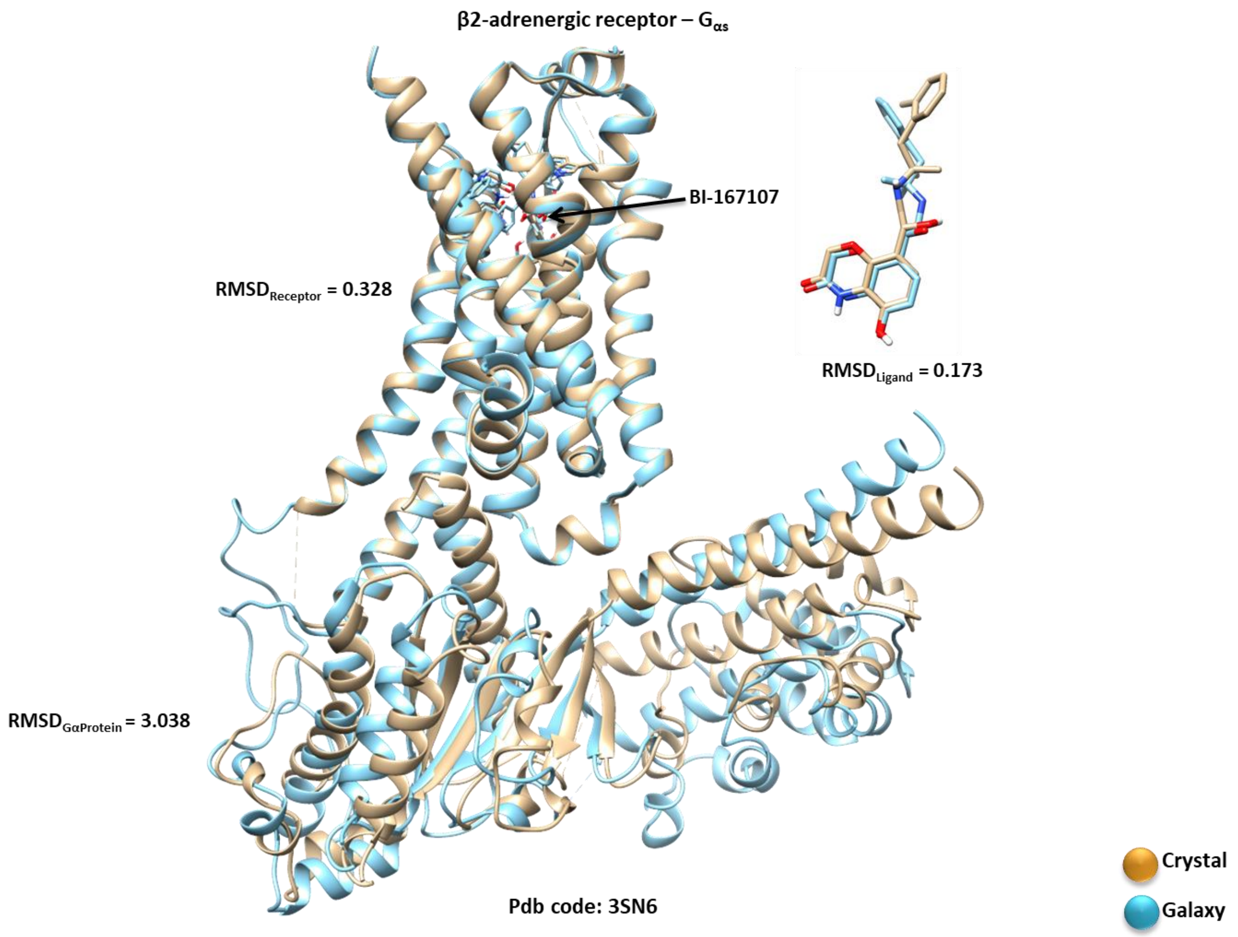

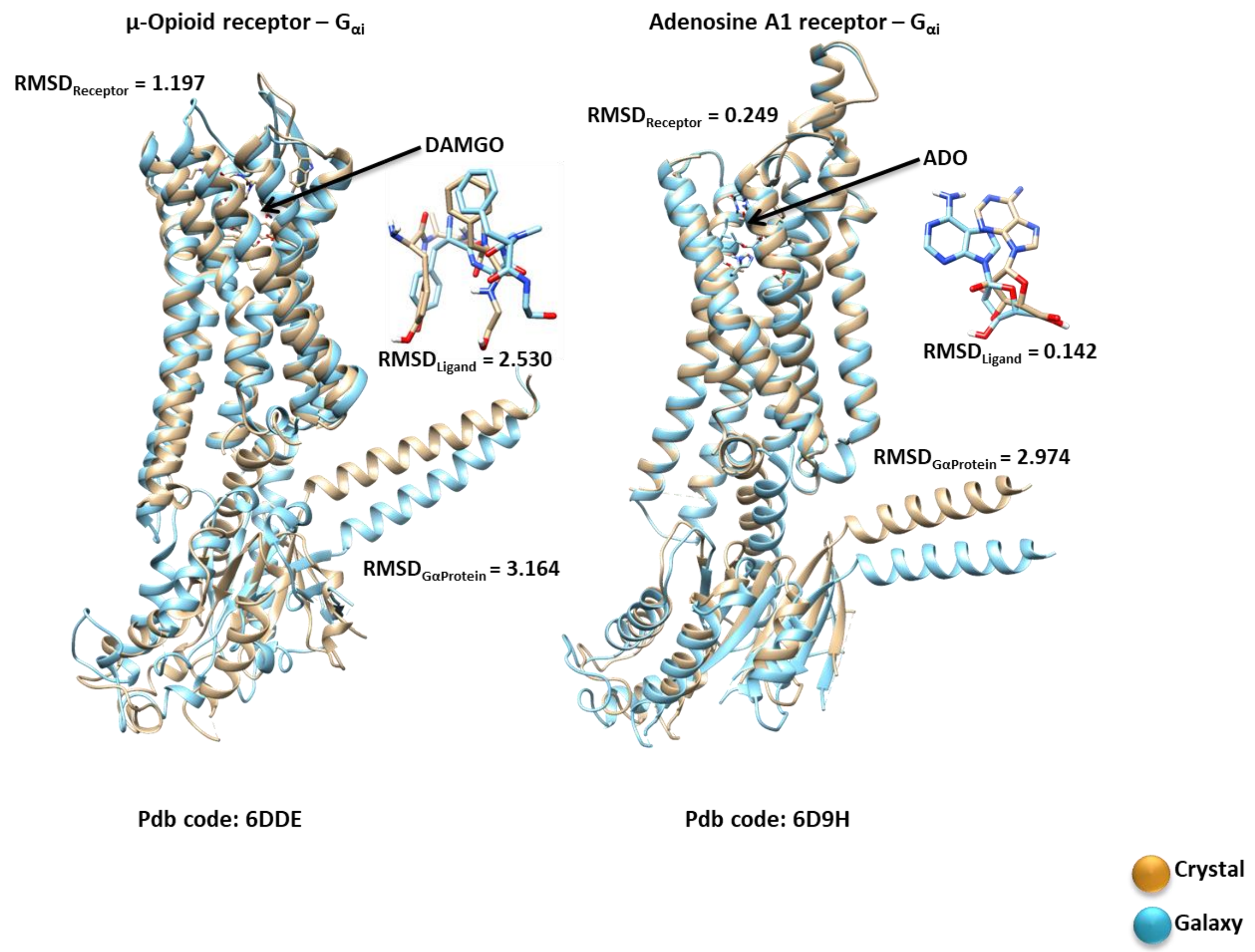
μ-Opioid receptor – Gαi
RMSDReceptor = 1.197
DAMGO
RMSDLigand = 2.530
RMSDGαProtein = 3.164
Pdb code: 6DDE
Adenosine A1 receptor – Gαi
RMSDReceptor = 0.249
ADO
RMSDLigand = 0.142
RMSDGαProtein = 2.974
Pdb code: 6D9H
Crystal
Galaxy

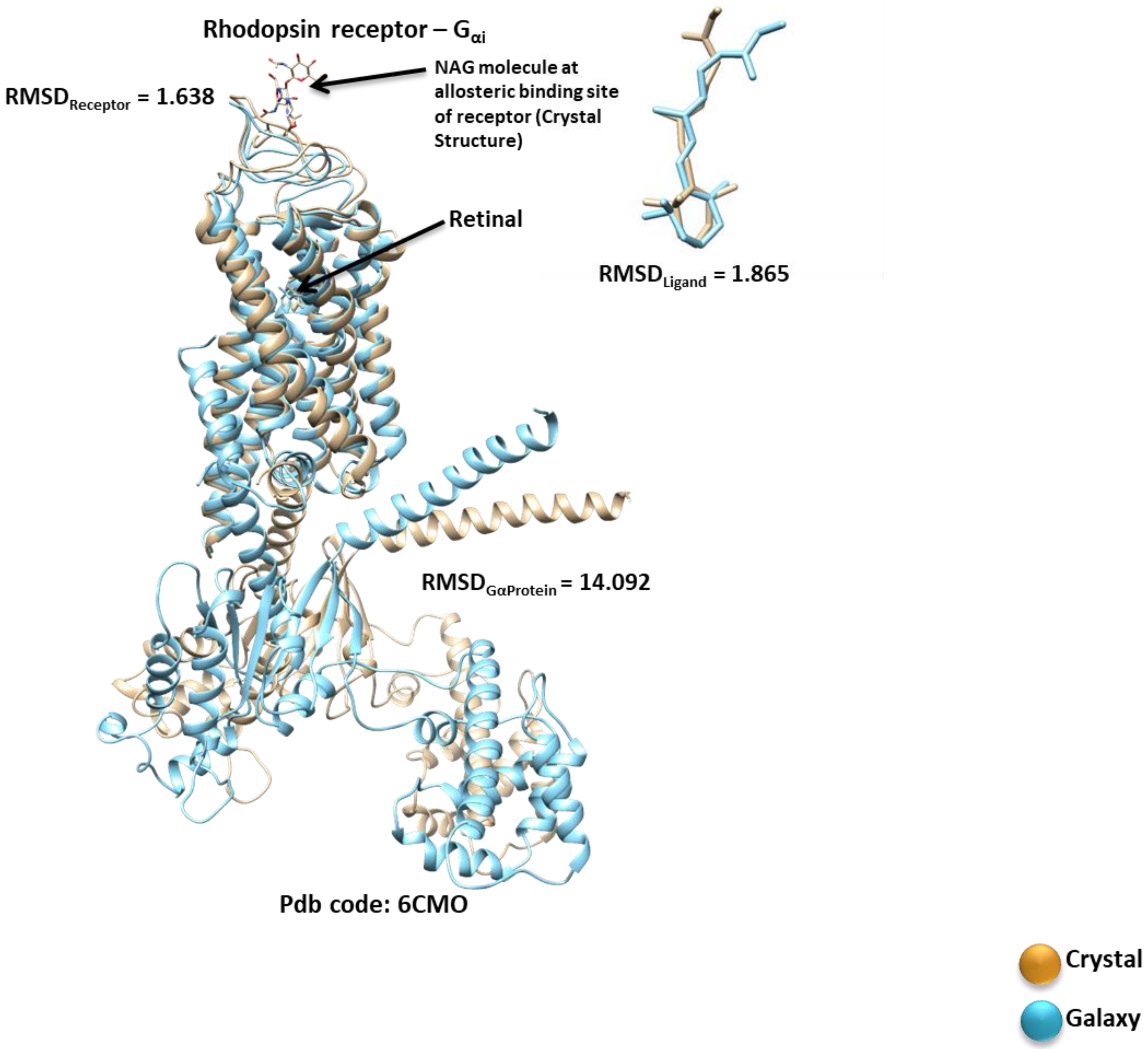
Rhodopsin receptor – Gαi
RMSDReceptor = 1.638
NAG molecule at allosteric binding site of receptor (Crystal Structure)
Retinal
RMSDLigand = 1.865
RMSDGαProtein = 14.092
Pdb code: 6CMO
Crystal
Galaxy

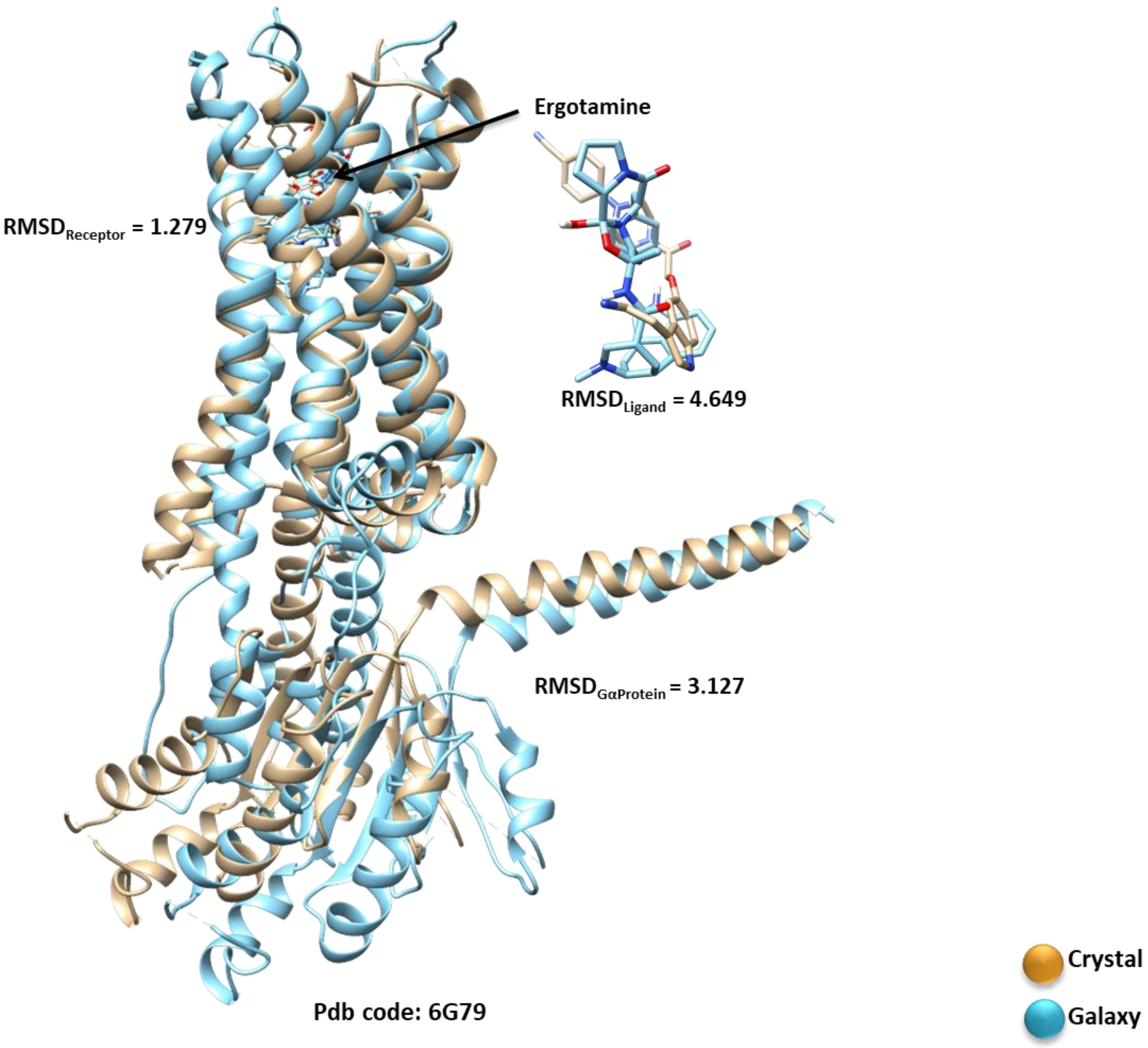

Figure depicts the comparison of the crystal structure of a liganded receptor (orange ribbon Crystal) and the retrieved solution of simulation with the same ligand, with the use of Galaxy 7 TM web server (blue, GalaxyWeb). Global RMSD is calculated with the Chimera program. Crystal pdb code is also depicted. In addition, the poses of the same ligand, extracted from the crystal structure and simulated by the GalaxyWeb program) are shown, and their RMSD is reported. In the case of the Rhodopsin receptor, the only available crystal is one with the ligand NAG bound to the allosteric site of a constitutively active receptor (PDB 6CMO (Kang et al., 2018)). In this case, we present retinal bound to a bovine rhodopsin receptor pose as compared to our model (compare also with https://pdb101.rcsb.org/motm/147).

For the serotonin receptor, the position of the protruding intracellular loop is hypothetical, as the crystal structures of the receptor lack supporting information. The existence of this loop has however also been

evidenced after modeling with GPCR-ModSim (http://open.gpcr-modsim.org/) (Esguerra et al., 2016) and Phyre2 (http://www.sbg.bio.ic.ac.uk/~phyre2/html/page.cgi?id=index) (Kelley et al., 2015) Web-servers.

Supplemental Figure 2

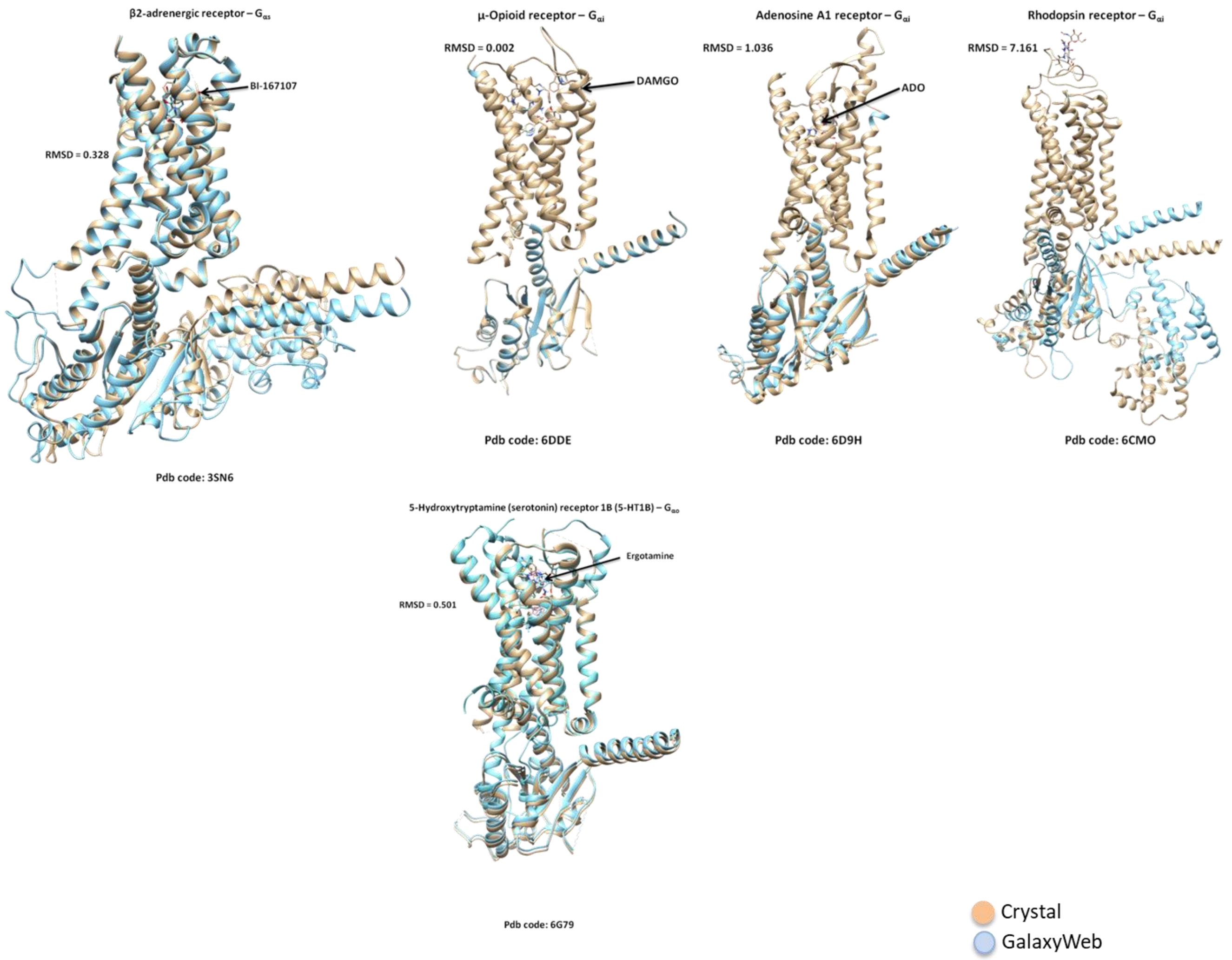

Figure depicts the comparison of the crystal structure of a liganded receptor-Gα-GDP hetero-complexes (orange ribbon Crystal) and the retrieved solution of simulation with the same ligand, with the use of Galaxy 7 TM web server and the HEX program (Cyan, GalaxyWeb). Global RMSD is calculated with the Chimera program. Crystal pdb code is also shown.

Supplemental Figure 3

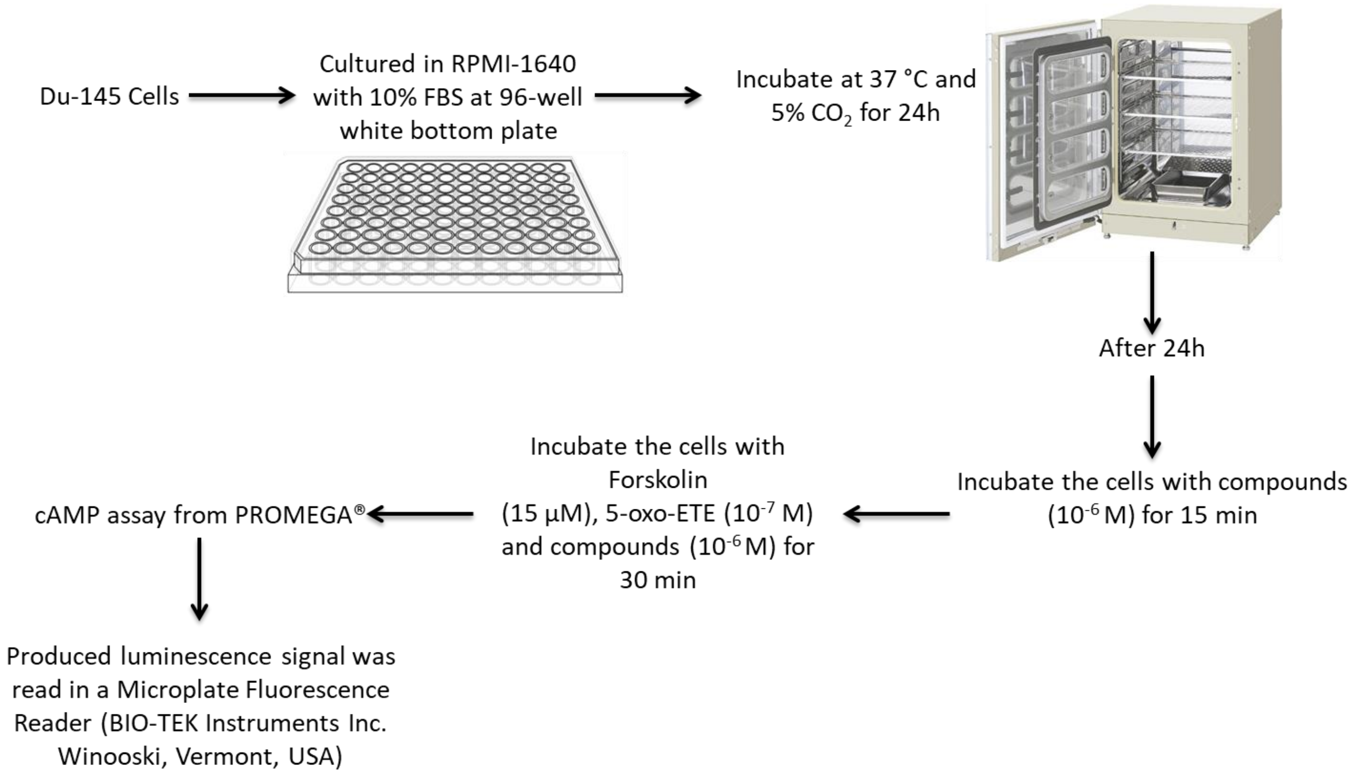

Figure presents the details of the cAMP production assay used for the identification of the inhibitory effect of compounds on OXER1 receptor (see (Kalyvianaki et al., 2017) for further details).

References

Esguerra M, Siretskiy A, Bello X, Sallander J and Gutierrez-de-Teran H (2016) GPCR-ModSim: A comprehensive web based solution for modeling G-protein coupled receptors. *Nucleic Acids Res* **44**(W1): W455-462.

Kalyvianaki K, Gebhart V, Peroulis N, Panagiotopoulou C, Kiagiadaki F, Pediaditakis I, Aivaliotis M, Moustou E, Tzardi M, Notas G, Castanas E and Kampa M (2017) Antagonizing effects of membrane-acting androgens on the eicosanoid receptor OXER1 in prostate cancer. *Sci Rep* **7**: 44418.

Kang Y, Kuybeda O, de Waal PW, Mukherjee S, Van Eps N, Dutka P, Zhou XE, Bartesaghi A, Erramilli S, Morizumi T, Gu X, Yin Y, Liu P, Jiang Y, Meng X, Zhao G, Melcher K, Ernst OP, Kossiakoff AA, Subramaniam S and Xu HE (2018) Cryo-EM structure of human rhodopsin bound to an inhibitory G protein. *Nature* **558**(7711): 553-558.

Kelley LA, Mezulis S, Yates CM, Wass MN and Sternberg MJ (2015) The Phyre2 web portal for protein modeling, prediction and analysis. *Nat Protoc* **10**(6): 845-858.

Supporting Information 2

Concise Manual of the pipeline used for *in silico* drug screening

Concise Manual of the pipeline used for *in silico* drug screening

Table of Contents

Introduction 2
Receptor Preparation 2
Existing crystal structure 2
Isolation of the receptor molecule 4
Non-existing crystal structure 6
Comparison of the crystal structure with the calculated model 9
Ligand preparation 14
Ligand-receptor docking 17
Ligand pose comparison 23
G-protein interactions 35
Gα protein model construction 35
Binding of Ga proteins with GDP or GTP 37
Binding of liganded receptor and liganded Gα protein 39

Introduction

The present detailed manual explaind the approach followed in the paper "A simple open source bio-informatic method for initial exploration of GPCR ligands' agonistic/antagonistic properties", by Athanasios A. Panagiotopoulos, Christina Papachristofi, Konstantina Kalyvianaki, Panagiotis Malamos, Panayiotis A. Theodoropoulos, George Notas, Theodora Calogeropoulou, Elias Castanas and Marilena Kampa.

The proposed algorithms are based on free programs and web sites, and provide detailed step-by-step intructions. The underlying rationale implicates the following steps:

1. Ligand and receptor preparation,
2. Ligand-receptor docking and
3. Gα-protein interaction.

We propose that, by modeling the Gα-GDP interaction with the liganded receptor, one can distinguish in silico the agonistic or antagonistic properties of a given ligand.

The steps leading to such a discrimination are described below.

Receptor Preparation

Existing crystal structure

If a crystal structure of a receptor already exists, one may download it directly from the online repository: https://www.rcsb.org/.

For example, the β_2-adrenergic receptor has been crystalized:

At the initial page of the PDB repository, type its name in the search field:

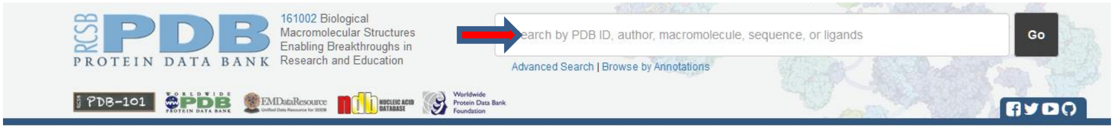

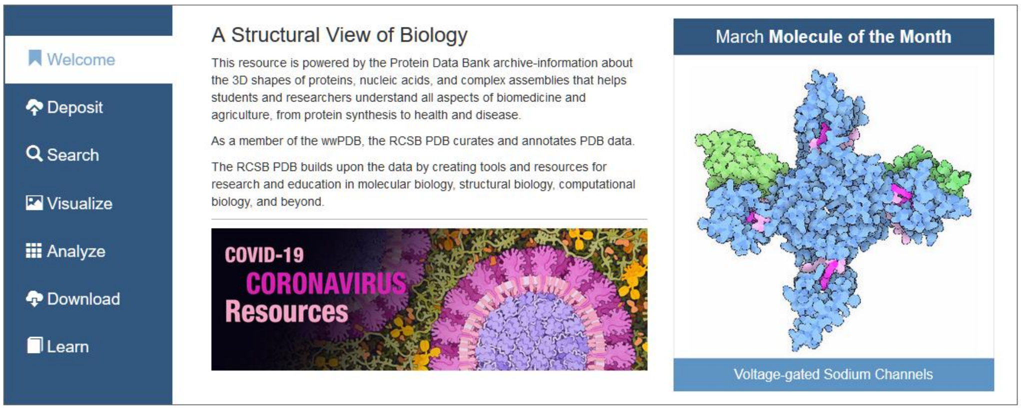

Restrict the search at a given species (ex Human):

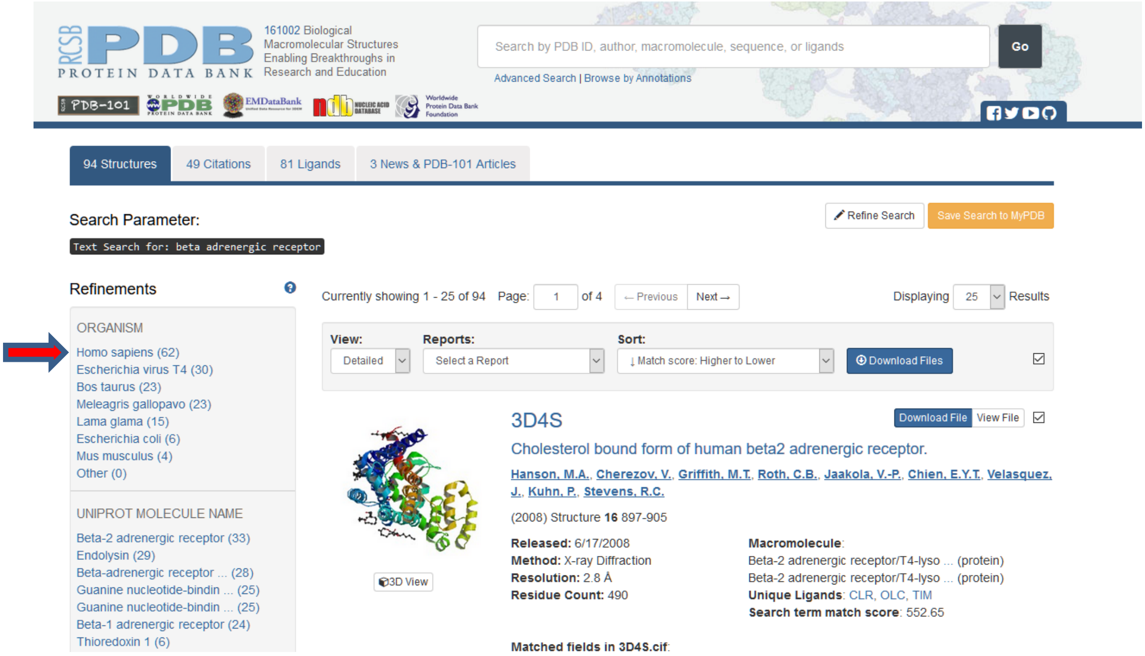

Let's choose a specific model:

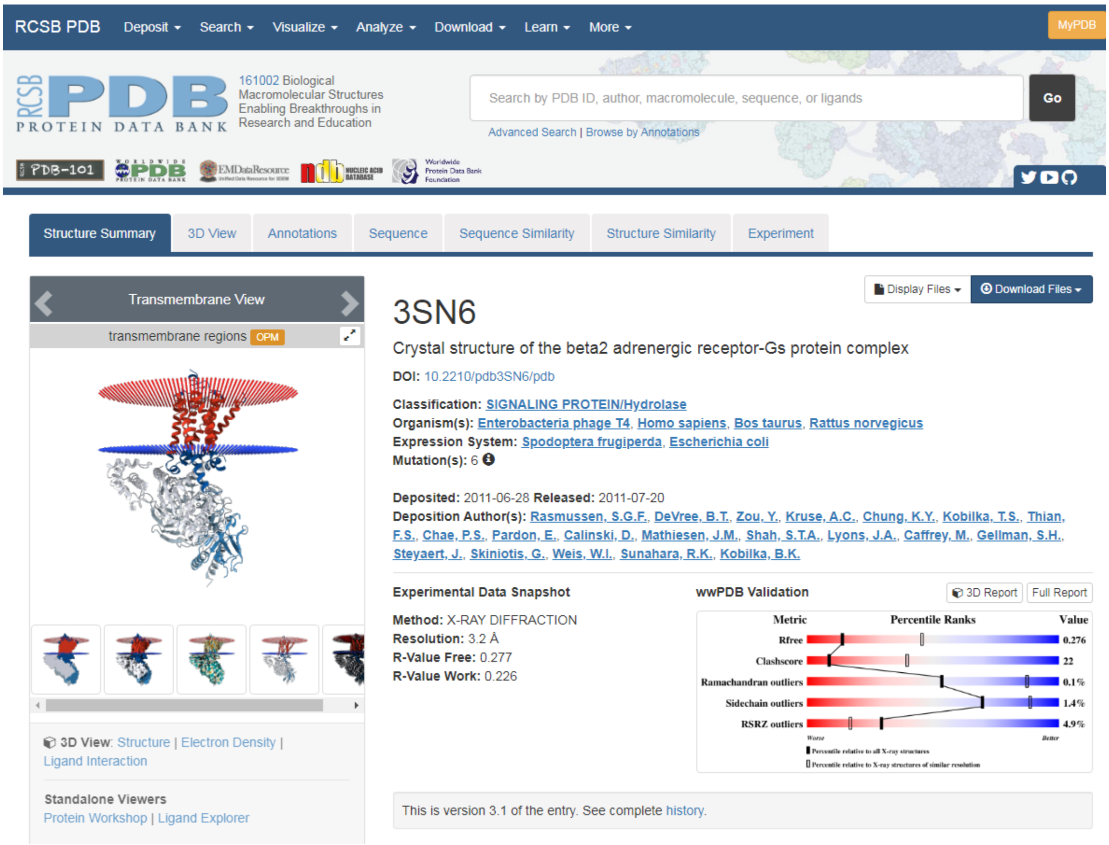

The specific model can be downloaded, in pdb format:

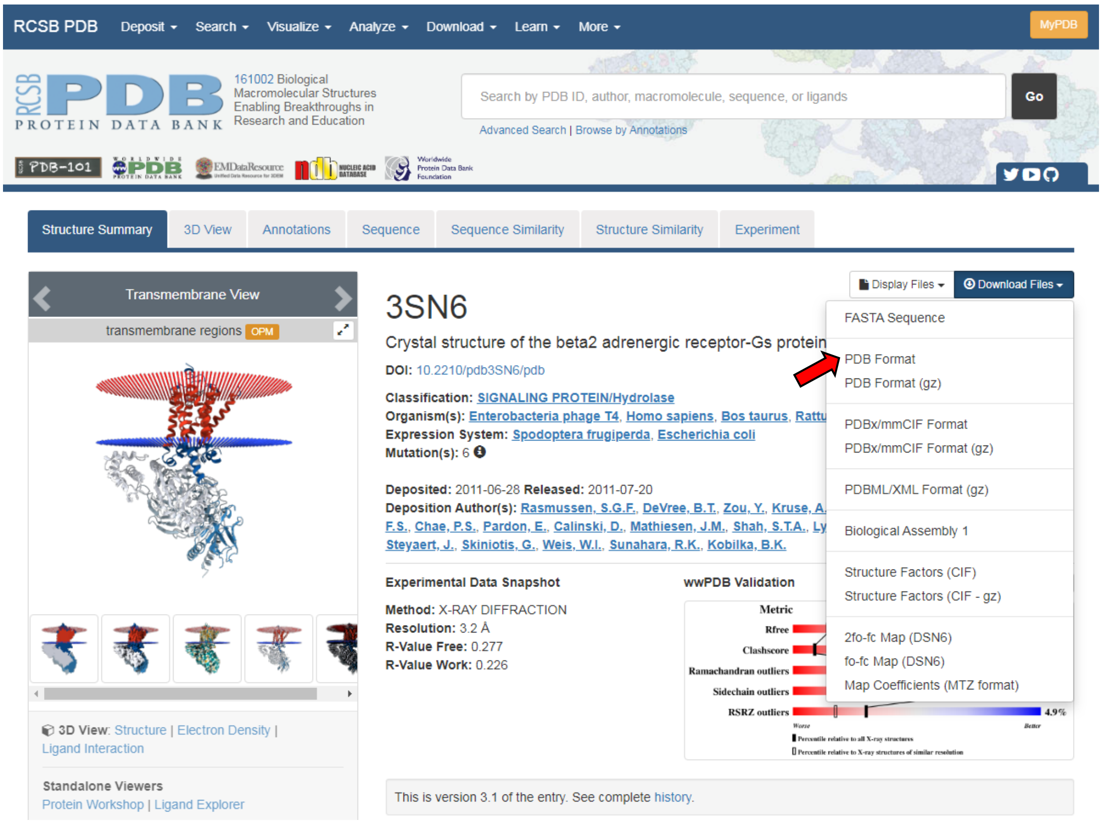

NOTE: Usually, crystals contain the receptor of interest, together with a specific ligand and/or associated with other proteins. In the provided example, the human beta-adrenergic receptor is associated with a Gαs protein, and the high affinity ligand BI-167107 is bound to its orthosteric binding groove. In such cases, please follow the following steps to isolate the receptor structure alone.

Isolation of the receptor molecule

Use a simple text editor (for example NotePad in windows, or the free editor EditPad Lite). In the provided example, search for the second incremental initiation of aminoacids and delete them:

```
ATOM   9279  CB  CYS R 341      40.858  24.535  16.354  1.00137.50           C
ANISOU 9279  CB  CYS R 341    15111  20594  16538   -671   1225    338       C
ATOM   9280  SG  CYS R 341      41.837  23.349  15.399  1.00138.40           S
ANISOU 9280  SG  CYS R 341    15372  20699  16516   -626   1287    221       S
TER    9281      CYS R 341
ATOM   9282  N   GLN N   1      40.237  24.525  61.779  1.00 68.65           N
ANISOU 9282  N   GLN N   1     6341   9017  10724    295    -17  -1794       N
ATOM   9283  CA  GLN N   1      39.194  23.588  62.179  1.00 68.30           C
ANISOU 9283  CA  GLN N   1     6255   9062  10632    296    -25  -1697       C
ATOM   9284  C   GLN N   1      37.852  24.018  61.605  1.00 62.83           C
ANISOU 9284  C   GLN N   1     5560   8397   9915    341     64  -1611       C
ATOM   9285  O   GLN N   1      37.798  24.845  60.704  1.00 77.18           O
ANISOU 9285  O   GLN N   1     7417  10157  11750    359    114  -1608       O
ATOM   9286  CB  GLN N   1      39.106  23.510  63.702  1.00 68.27           C
ANISOU 9286  CB  GLN N   1     6196   9138  10606    309    -31  -1760       C
ATOM   9287  CG  GLN N   1      38.333  22.312  64.221  1.00 71.74           C
ANISOU 9287  CG  GLN N   1     6594   9666  10997    293    -69  -1669       C
ATOM   9288  CD  GLN N   1      38.419  22.191  65.722  1.00 87.18           C
ANISOU 9288  CD  GLN N   1     8531  11686  12906    293    -88  -1725       C
ATOM   9289  OE1 GLN N   1      38.006  21.187  66.301  1.00 93.82           O
ANISOU 9289  OE1 GLN N   1     9358  12593  13698    270   -133  -1660       O
ATOM   9290  NE2 GLN N   1      38.964  23.218  66.366  1.00 90.77           N
ANISOU 9290  NE2 GLN N   1     9049  12104  13335    310    -54  -1816       N
ATOM   9291  N   VAL N   2      36.775  23.429  62.110  1.00 43.52           N
ANISOU 9291  N   VAL N   2     3071   6038   7426    355     79  -1536       N
ATOM   9292  CA  VAL N   2      35.423  23.831  61.745  1.00 47.06           C
ANISOU 9292  CA  VAL N   2     3512   6523   7846    401    165  -1454       C
ATOM   9293  C   VAL N   2      34.551  23.861  62.990  1.00 62.21           C
[illegible]
```

Using the same approach, extract also the ligand.

Control the pdb structure by inserting and drawing it in Chimera program (any other molecular drawing program, for example PyMOL, or VMD) can be used.

We can open the structure of receptor and ligand with Chimera program.

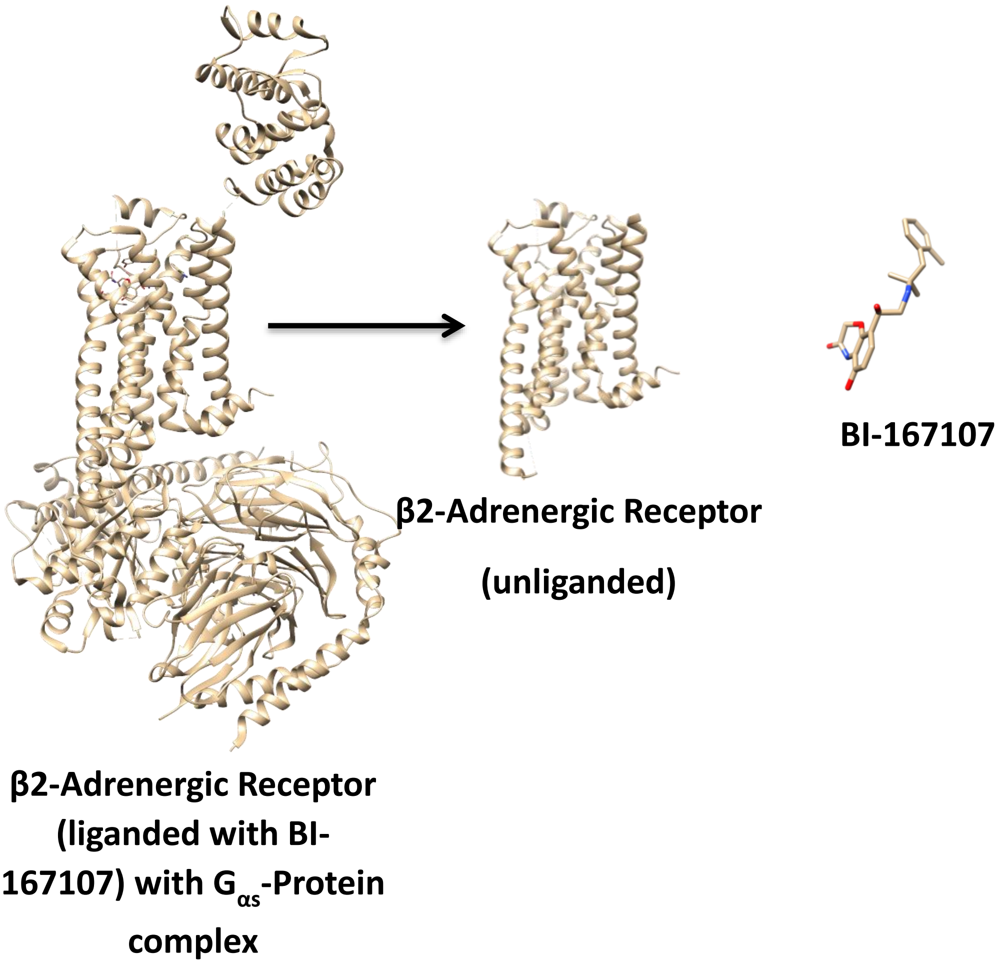

Non-existing crystal structure

The sequence of receptors, in fasta format, were retrieved from the NCBI protein database (https://ncbi.nlm.nih.gov/protein).

For example, for β_2-adrenergic receptor:

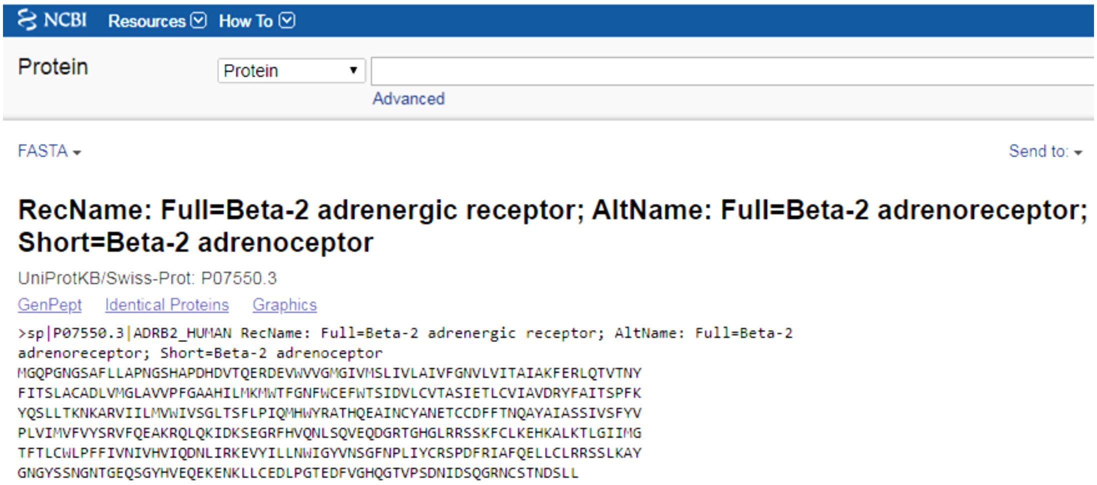

NCBI Resources How To

Protein Protein Advanced

FASTA Send to:

RecName: Full=Beta-2 adrenergic receptor; AltName: Full=Beta-2 adrenoreceptor; Short=Beta-2 adrenoceptor

UniProtKB/Swiss-Prot: P07550.3

GenPept Identical Proteins Graphics

```
>sp|P07550.3|ADRB2_HUMAN RecName: Full=Beta-2 adrenergic receptor; AltName: Full=Beta-2
adrenoreceptor; Short=Beta-2 adrenoceptor
MGQPGNGSAFLLAPNGSHAPDHDVTQERDEVWVVGMGIVMSLIVLAIVFGNVLVITAIAKFERLQTVTNY
FITSLACADLVMGLAVVPFGAAHILMKMWTFGNFWCEFWTSIDVLCVTASIETLCVIAVDRYFAITSPFK
YQSLLTKNKARVIILMVWIVSGLTSFLPIQMHWYRATHQEAINCYANETCCDFFTNQAYAIASSIVSFYV
PLVIMVFVYSRVFQEAKRQLQKIDKSEGRFHVQNLSQVEQDGRTGHGLRRSSKFCLKEHKALKTLGIIMG
TFTLCWLPFFIVNIVHVIQDNLIRKEVYILLNWIGYVNSGFNPLIYCRSPDFRIAFQELLCLRRSSLKAY
GNGYSSNGNTGEQSGYHVEQEKENKLLCEDLPGTEDFVGHQGTVPSDNIDSQGRNCSTNDSLL
```

The obtained sequence introduced to the Swiss Model Biospace (http://swissmodel.expasy.org). The following steps are necessary:

1. Start the modeling:

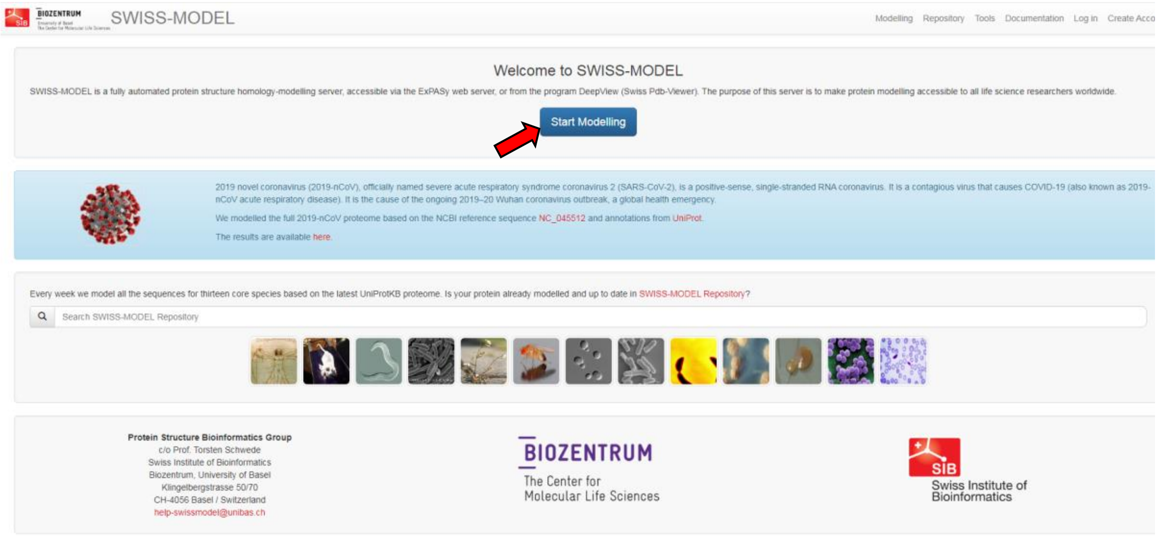

2. The retrieved sequence of the receptor is pasted in the adequate box:

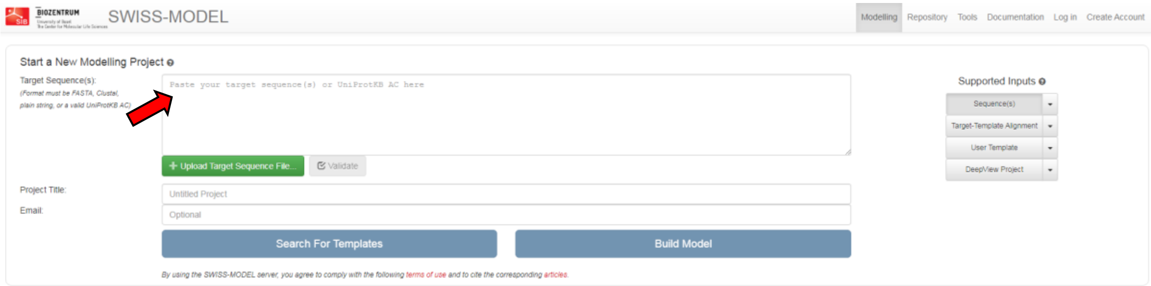

3. Push the button “Search for Templates”

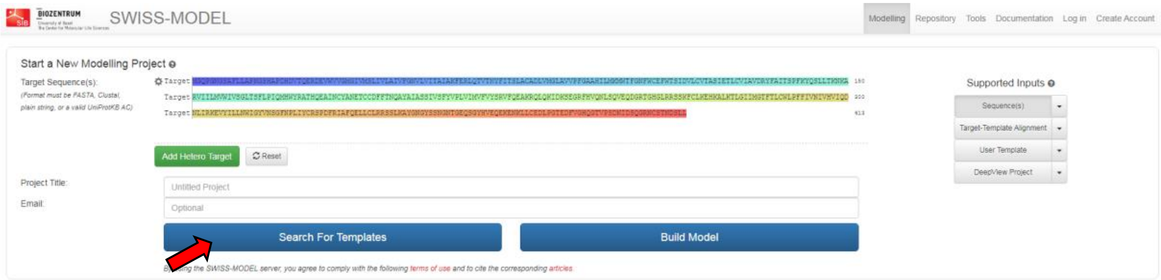

The web server searches for adequate templates:

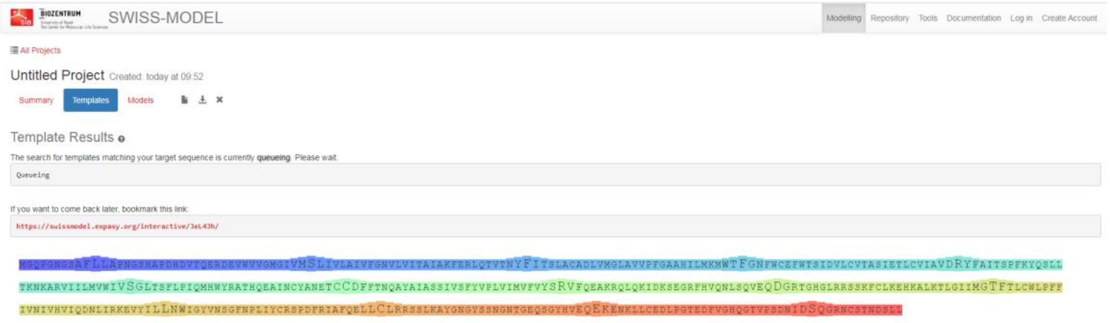

From the provided tabe, one should choose from the list, a template presenting the highest identity score. Then push the button “Build Models”:

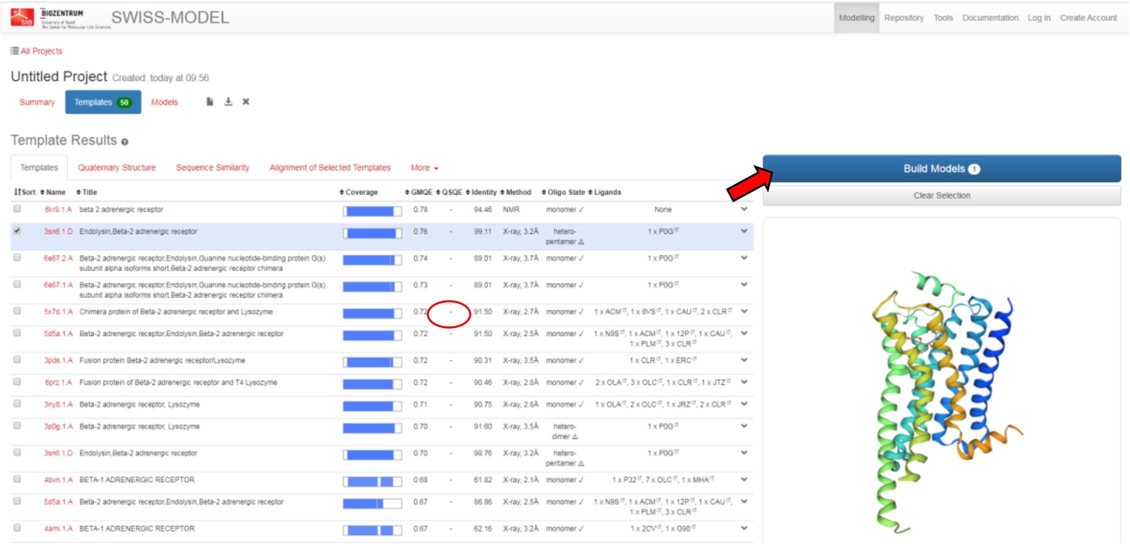

The server returns a 3D model, based on the chosen template. Please, inspect thoroughfully the model, although its final 3D structure may be modified during the fully flexible binding calculations, described below.

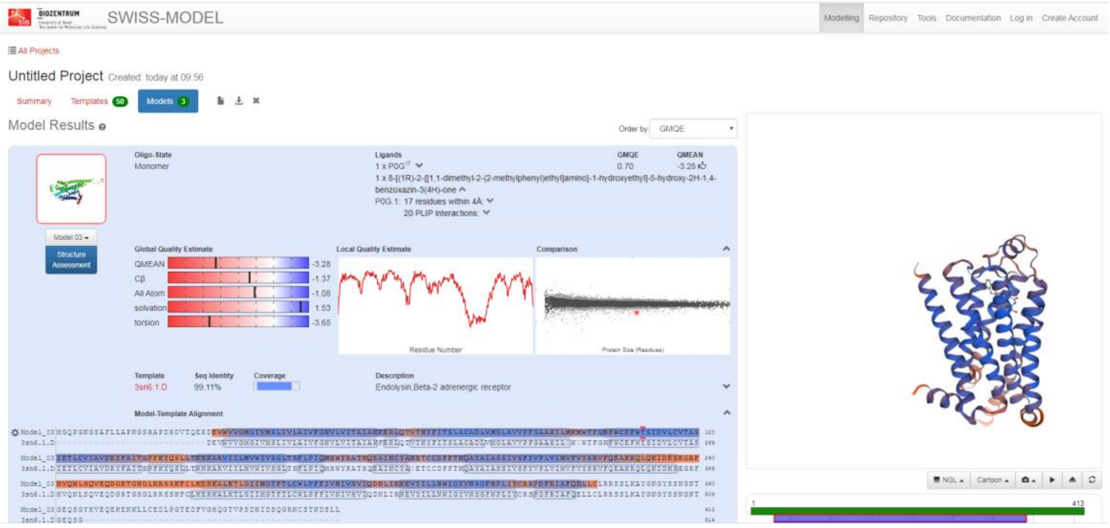

Download the model, in PDB format, by clicking the appropriate link.

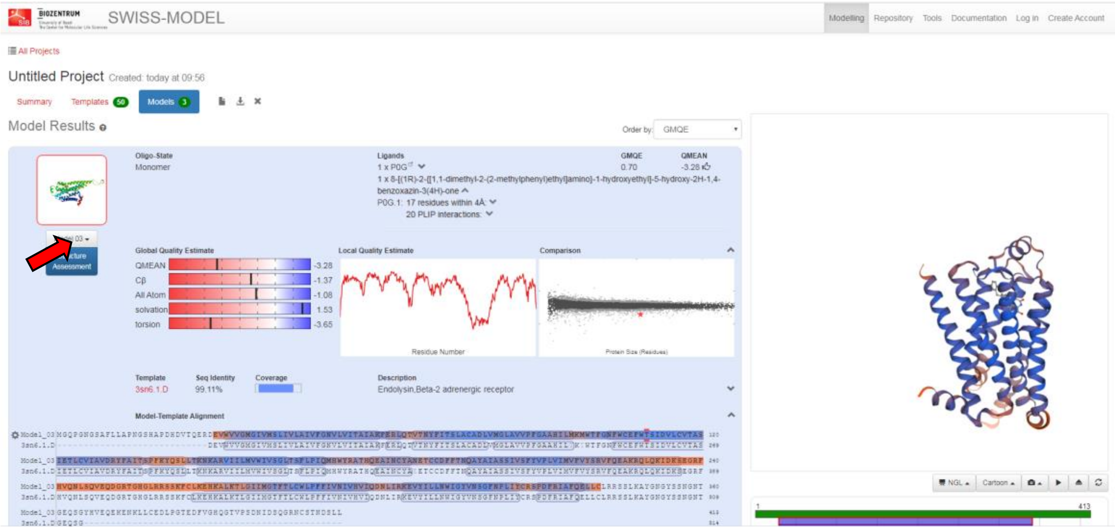

Comparison of the crystal structure with the calculated model

In any case that one wants to compare a crystal structure with the obtained bioinformatic solution, a superposition of the two molecules with a graphic program (here we used Chimera, but also pyMOL of VMD can also be used) is made.

As an example, we compare the extracted beta-adrenergic receptor from crystal 3SN6 and the obtained solution, as presented above:

We open the crystal structure of the receptor and the model from bioinformatic solution in Chimera program.

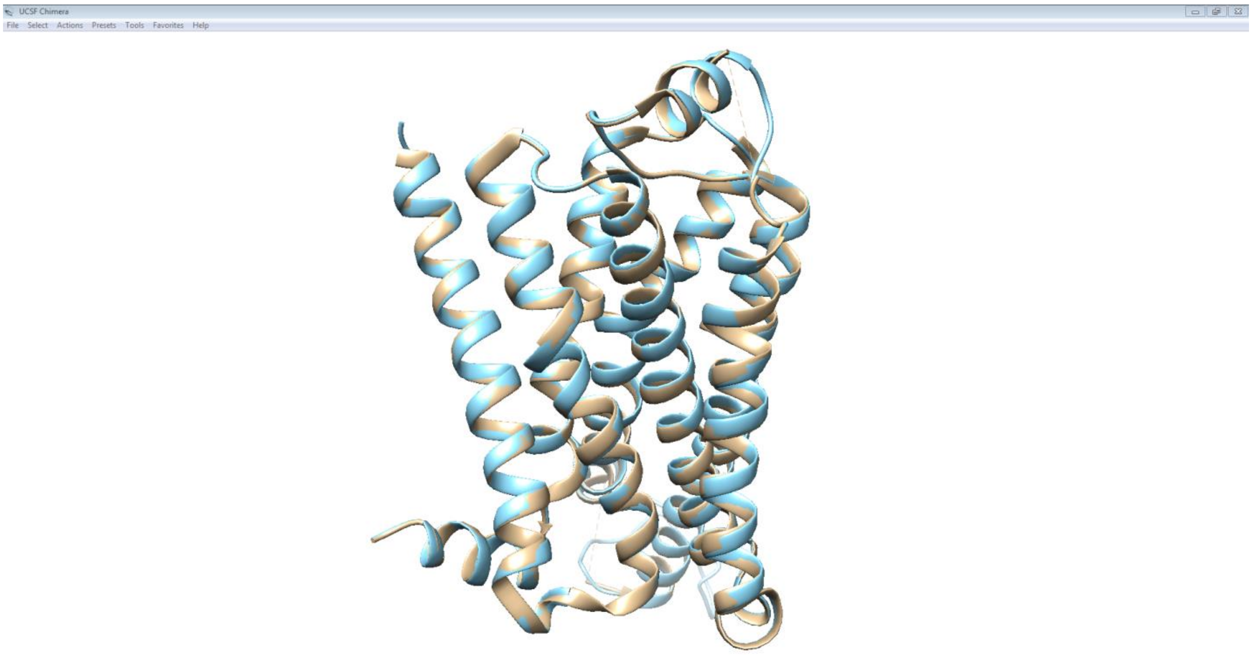

Compare the two structures using the MatchMaker tool from Structure Comparison Tools.

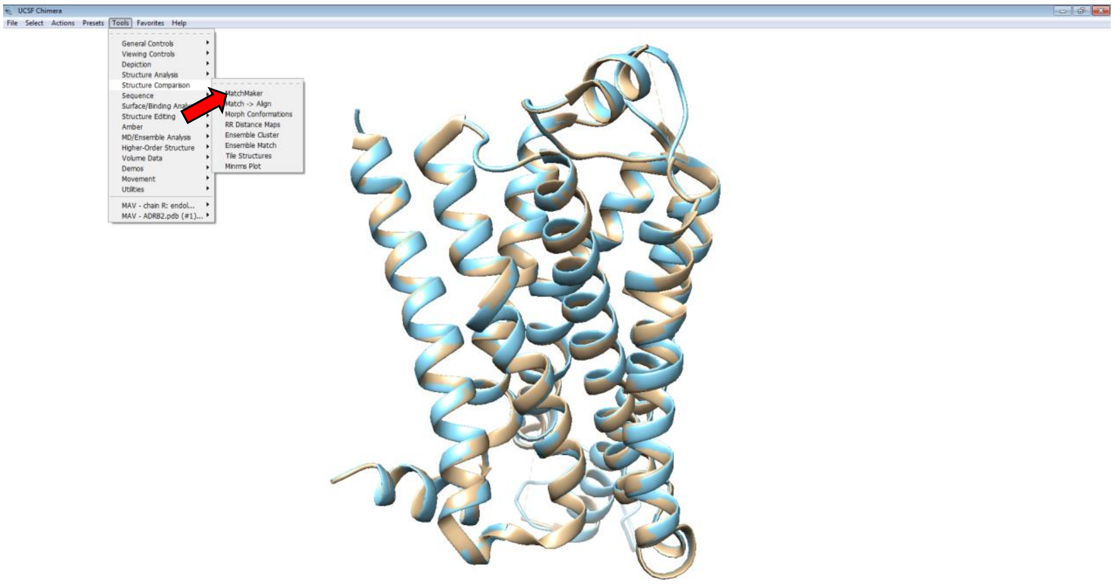

Select the first receptor as ’’Reference structure’’ and the second receptor as ’’Structure to match’’. Also, select the option ’’After superposition, compute structure-based multiple sequence aligment’’ and finally, apply the MatchMaker.

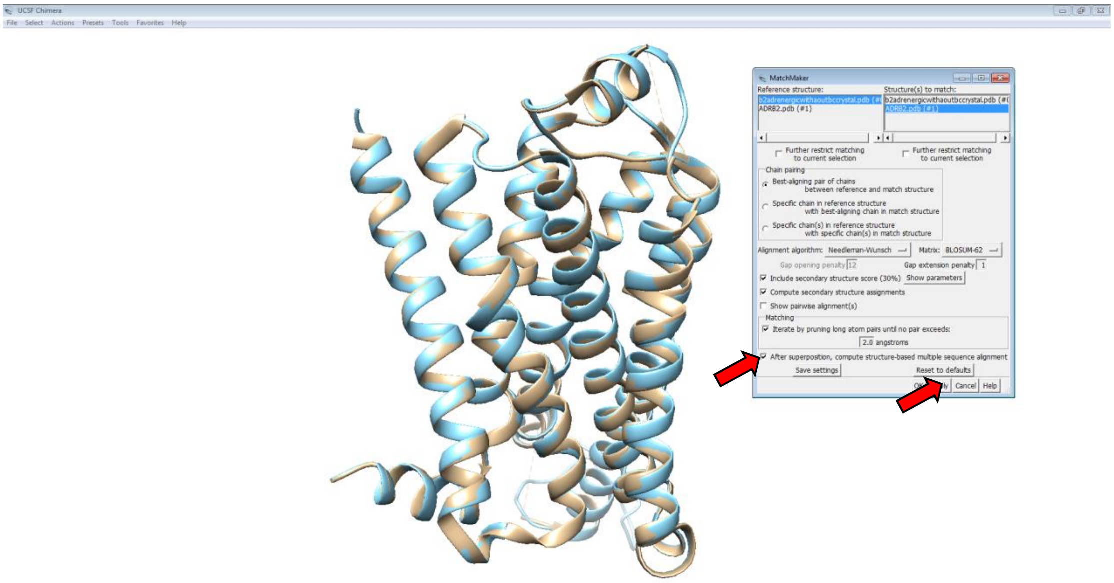

In the window that appears select ‘’Apply’’, to display the receptor sequences.

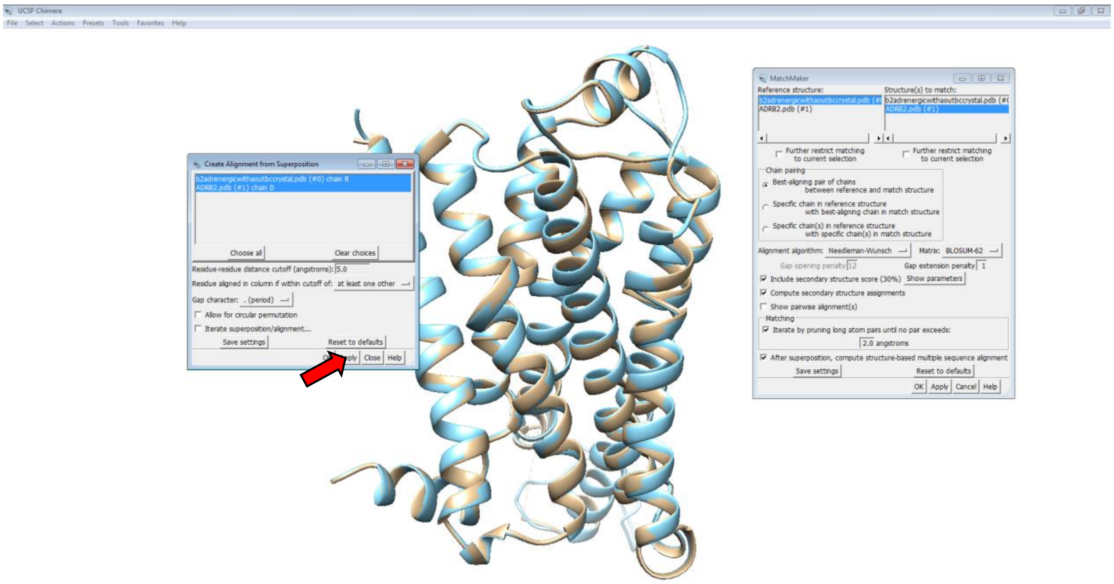

The sequences of the receptors being compared is displayed.

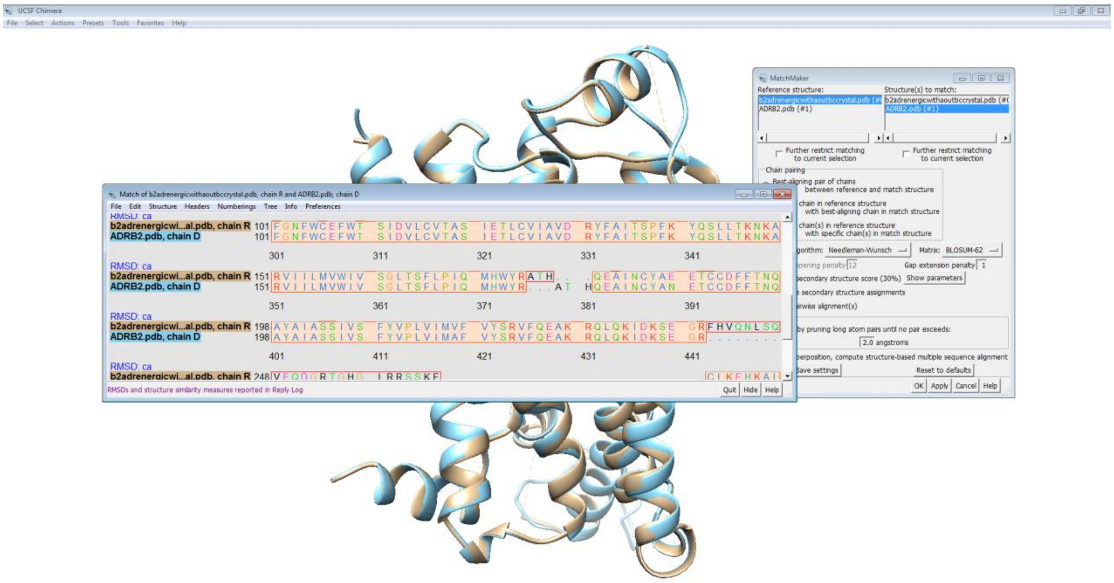

Select all amino acids to calculate the RMSD (root-mean-square deviation of atomic positions) of each region.

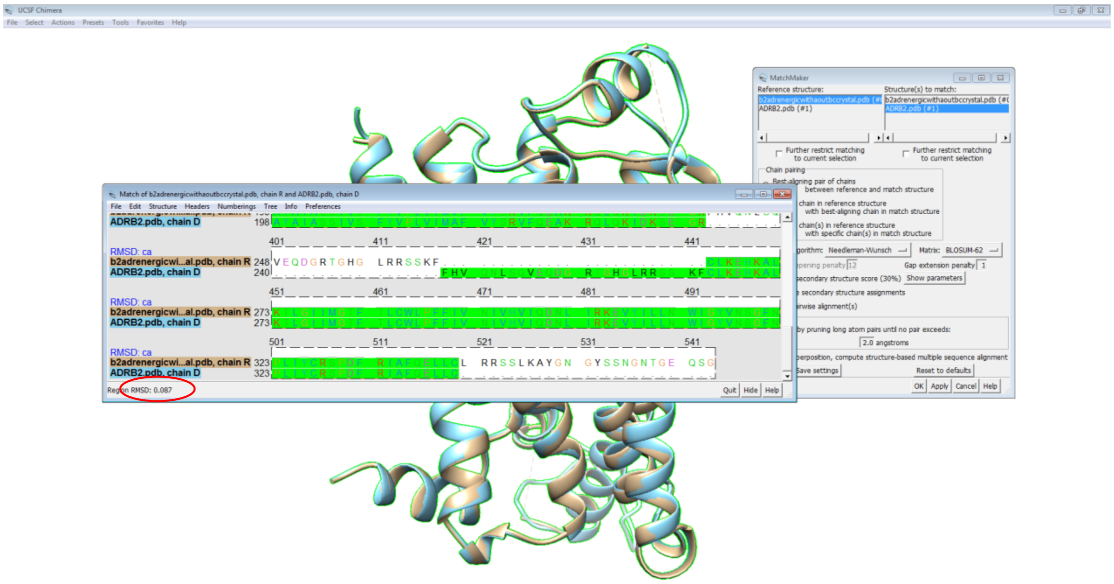

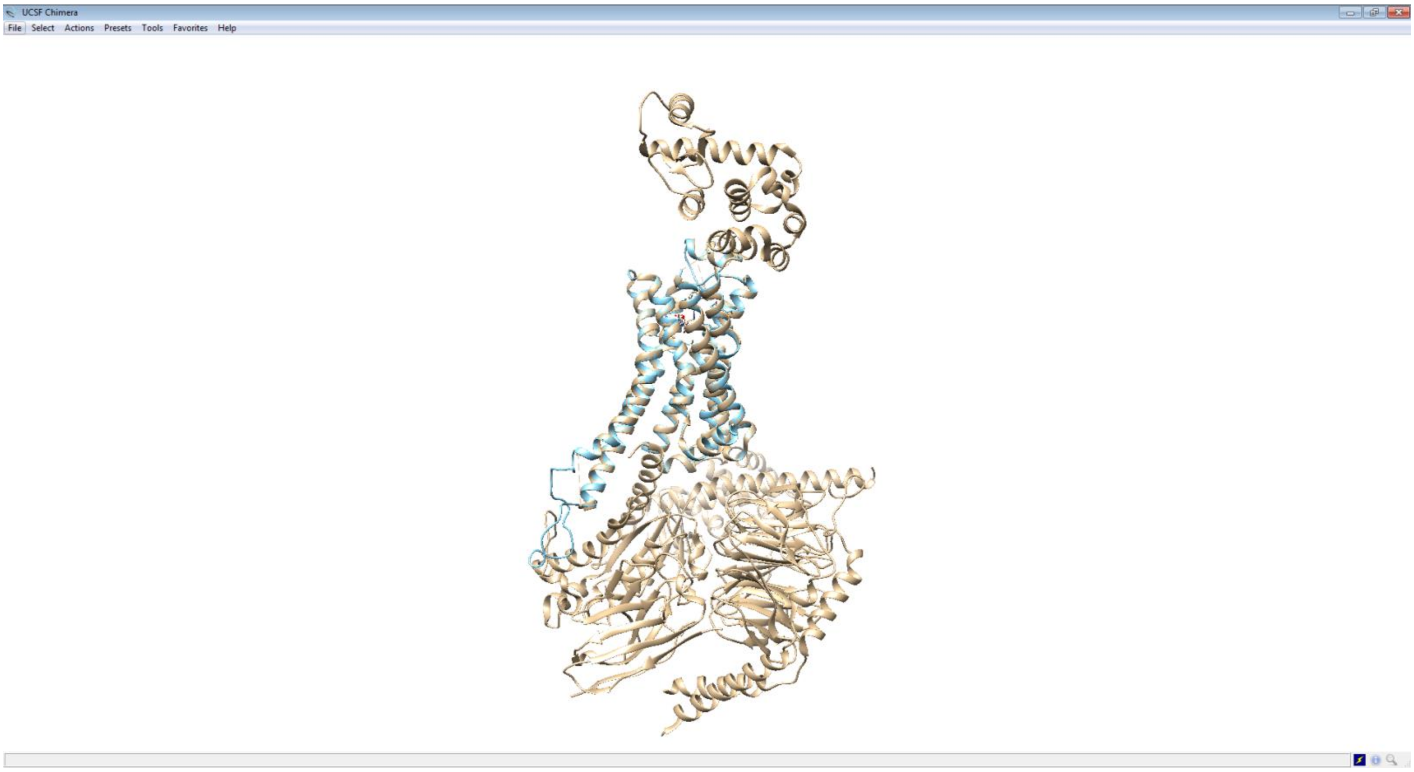

Also, we can keep only the structure of the receptor for the comparison:

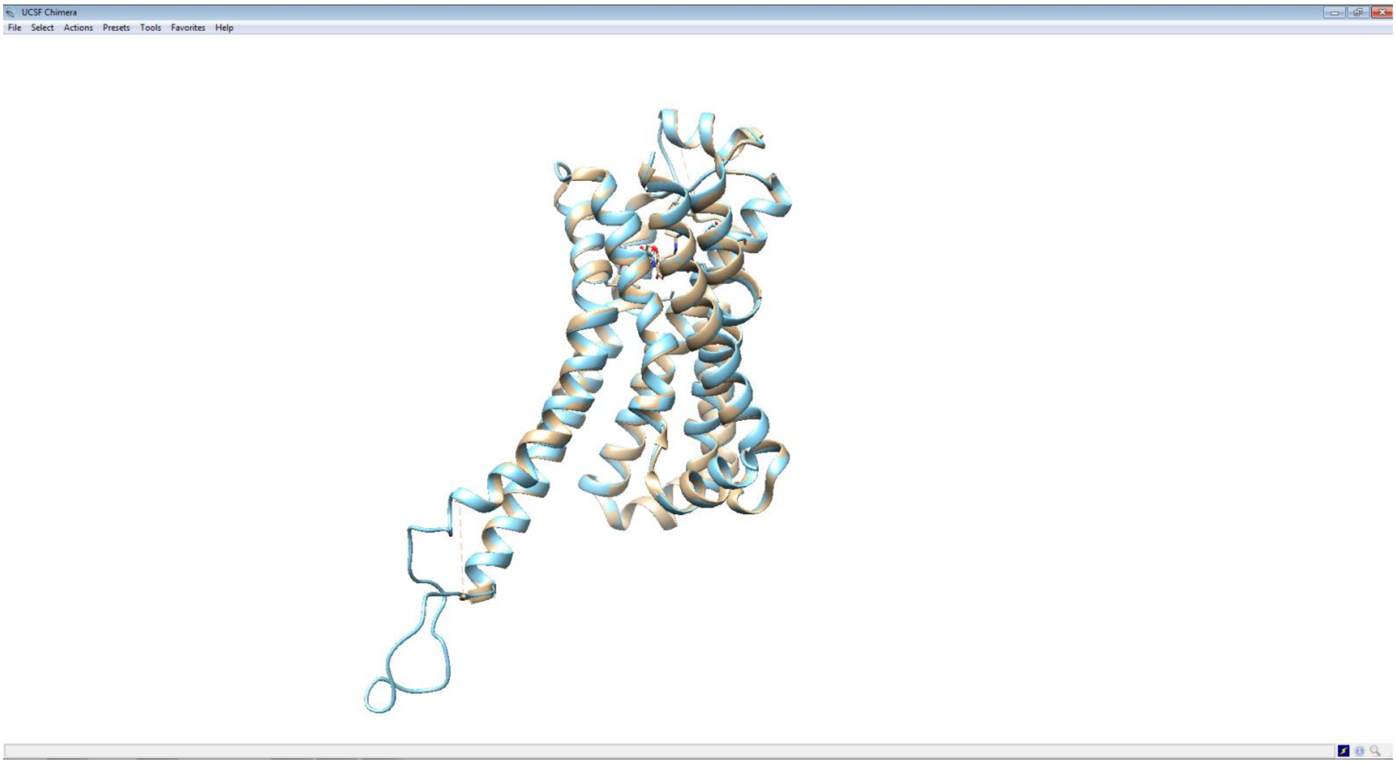
UCSF Chimera
File Select Actions Presets Tools Favorites Help

NOTE: The Swiss Model (as well as the Galaxy Web server) provide models based on the coverage homology of the GPCRs. However, other free servers (for example the GPCR ModSim or the GPCR-SSFE server), which perform a sequence alignment of the GPCR proteins provide very different conformations, with possible gaps in the receptor model. In the next figure, OXER1 models produced with the four different programs are shown.

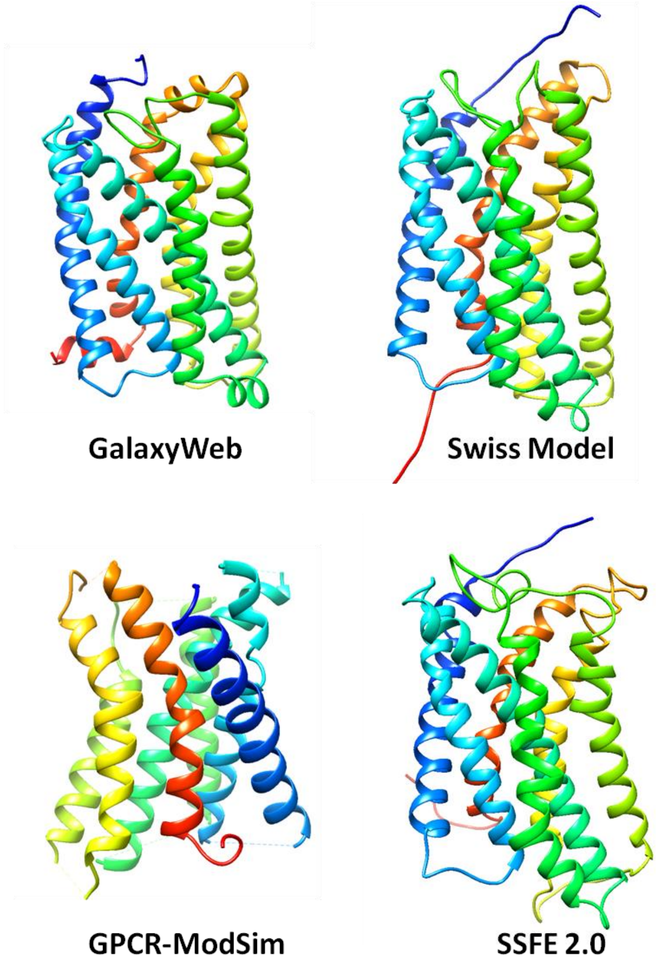

Ligand preparation

For ligand preparation, if we dealt with known molecules, their structures were retrieved from the ZINC database (http://zinc.docking.org/), usually in a canonical smiles format.

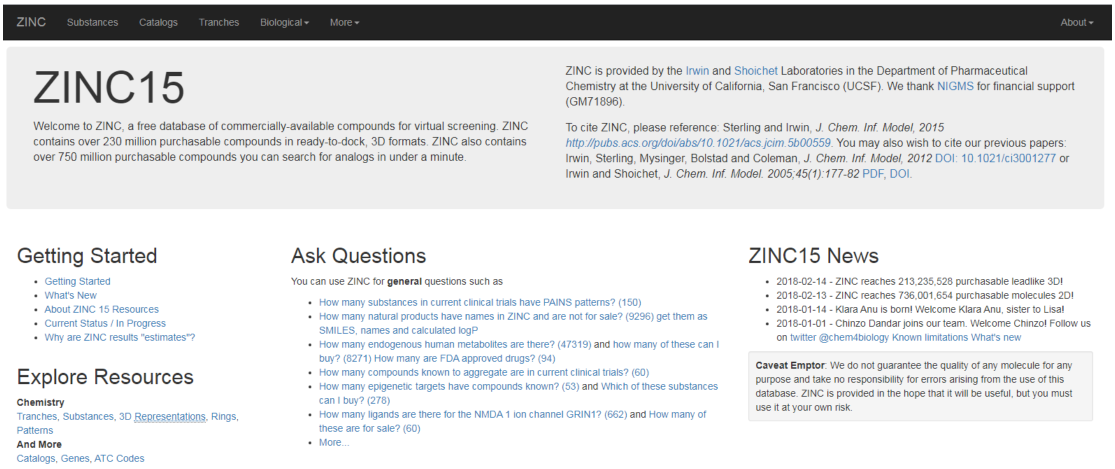

Novel molecules can be designed in ChemBioDraw (v12.0, Perkin Elmer, Boston, MA, free for Academic use). Other free drawing programs (ex ChemSketch, https://www.acdlabs.com/resources/freeware/chemsketch/, BKChem, http://bkchem.zirael.org/, or Symyx Draw, https://symyx-draw.jaleco.com/), can also be used.

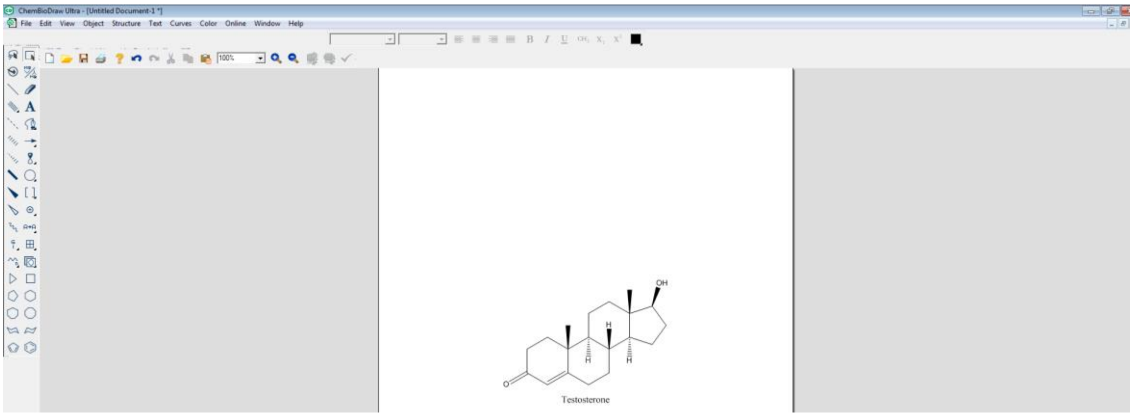

We can copy the smiles of a molecule from ChemBioDraw, if we click here:

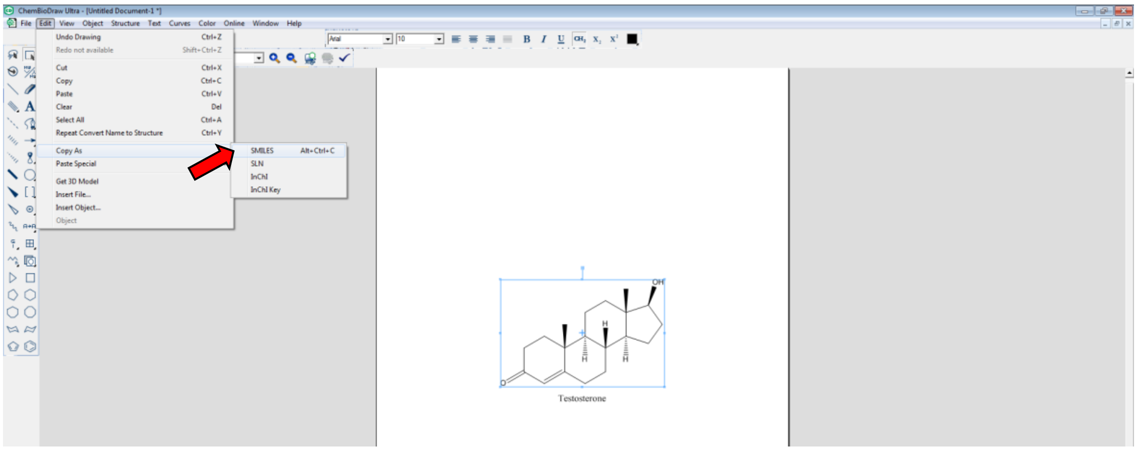

The structures were also transformed into a canonical smiles format. Pdb files were created with the Open Babel program (http://openbabel.org).

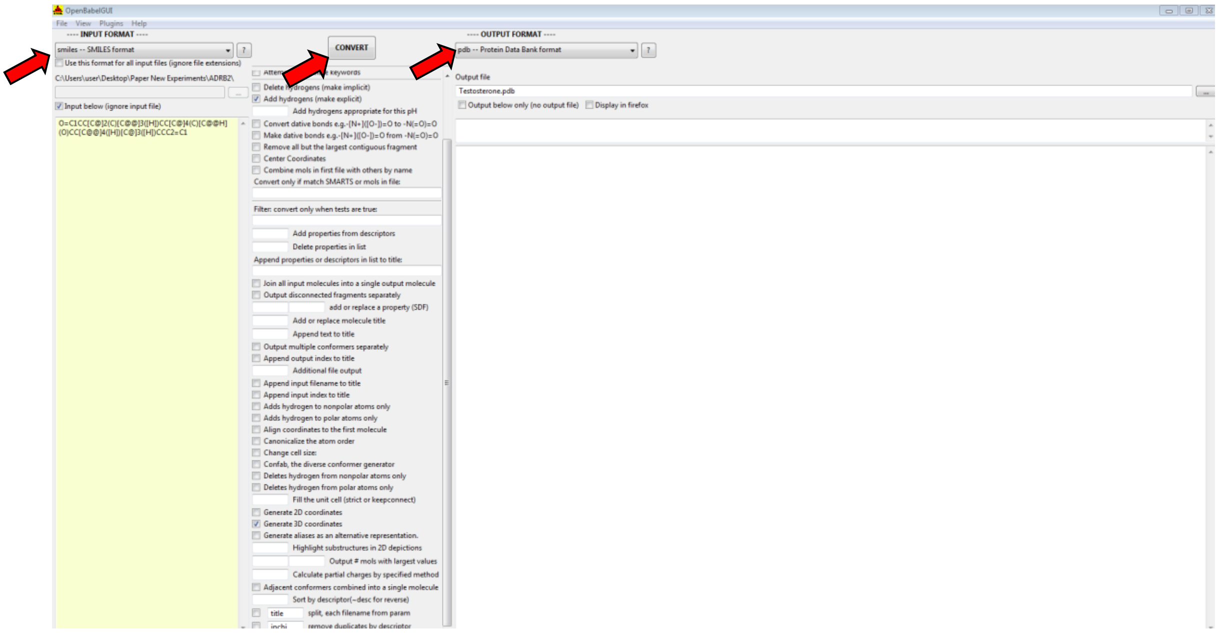

Ligand-receptor docking

Flexible docking algorithms (which are calculation-expensive) can be broadly divided into methods in which flexibility is attained during the ligand-binding interaction (on-the-fly methods) and methods applying multiple receptor or ensemble poses, at the beginning or during the simulations. As our goal was to provide a solution, applicable to novel GPCRs, in which experimental and/or crystallographic data might not be available, we have opted for on-the-fly approach. We have used the online server GalaxyWeb (http://galaxy.seoklab.org/), in which a full on-the-fly ligand and receptor flexibility is permitted. This server is based on the GalaxyDock2 algorithm, which, after automatic prediction of the ligand binding pocket, permits a full ligand/receptor flexibility. This step is followed by an optimization and a subsequent refinement, through a specific algorithm named GalaxyRefine, which permits a protein-ligand structure refinement, by applying iterative side chain repacking and overall structure relaxation. This server and the applied routines overperform solutions based on the MODELER algorithm, in different comparisons, including CASP9 & 10 and CAMEO. Its ranking was 5/13 web servers in the last CAPRI 2017 contest (http://www.capri-docking.org/resources/). The 3D structures of the liganded and unliganded receptor were compared, using the UCSF Chimera program.

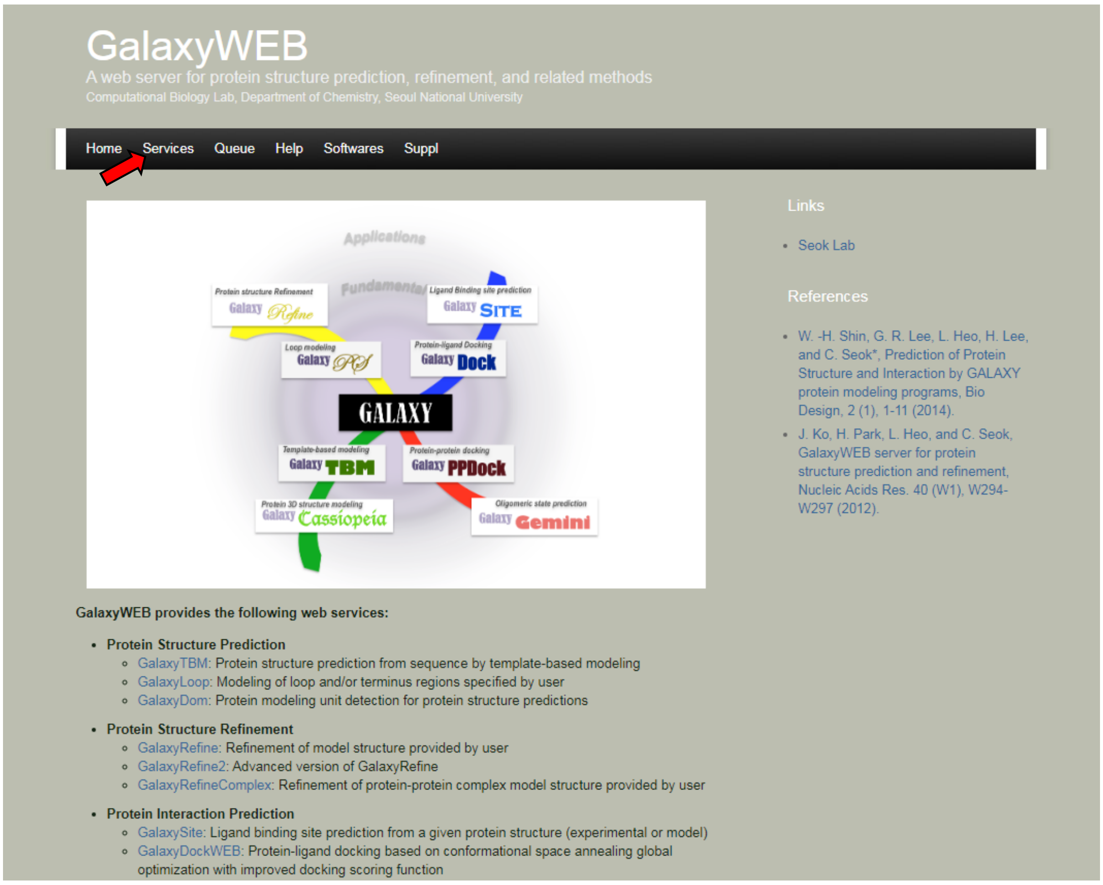

First select the **Site Service**, to find the binding site of the receptor:

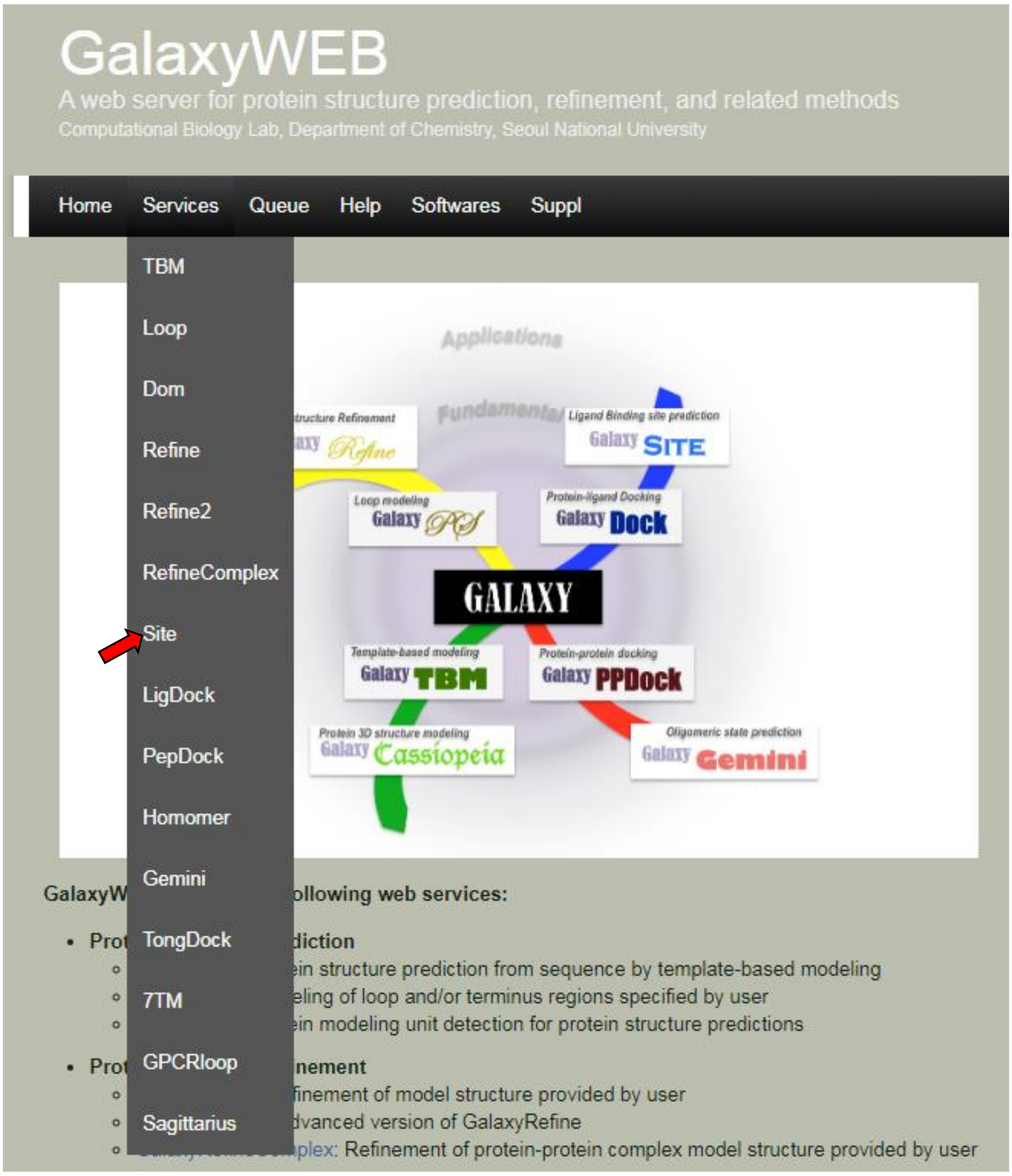

In the GalaxySite page service, fill the details of the receptor

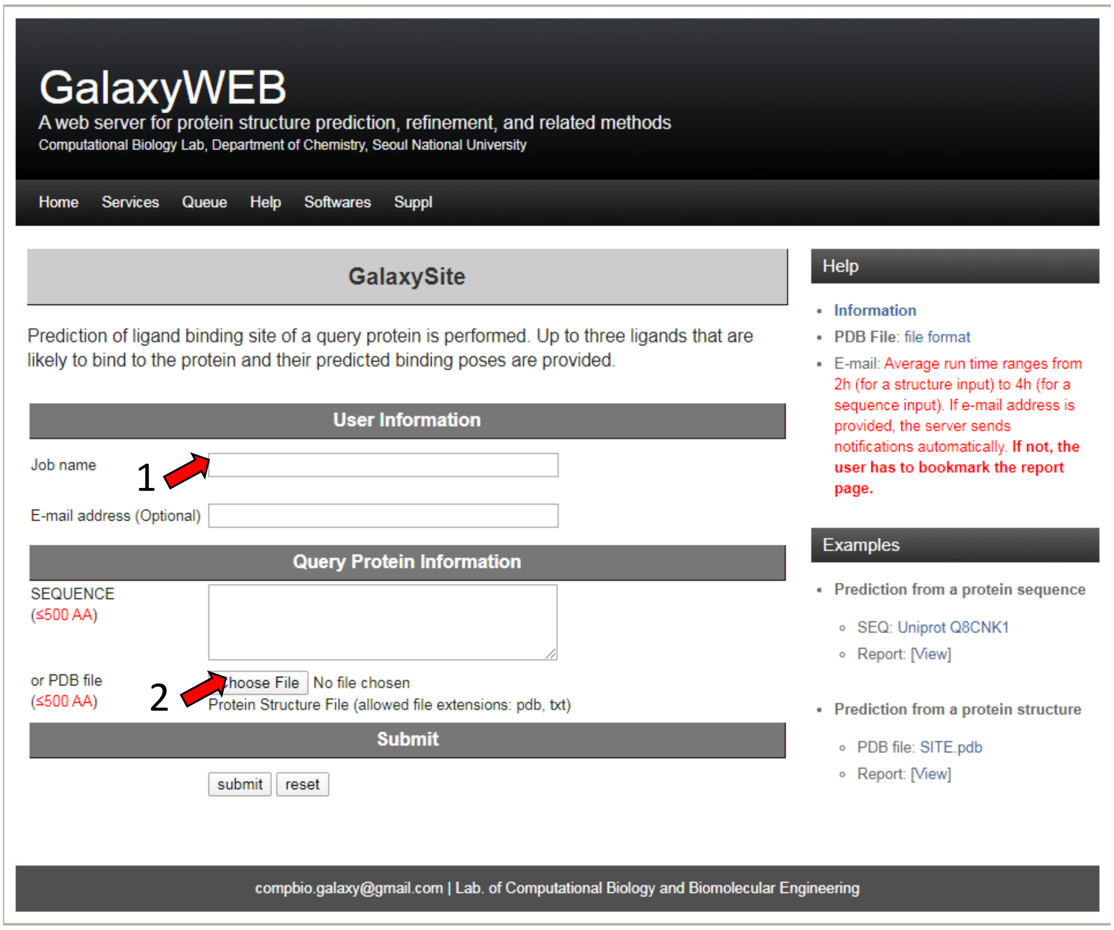

and submit the job:

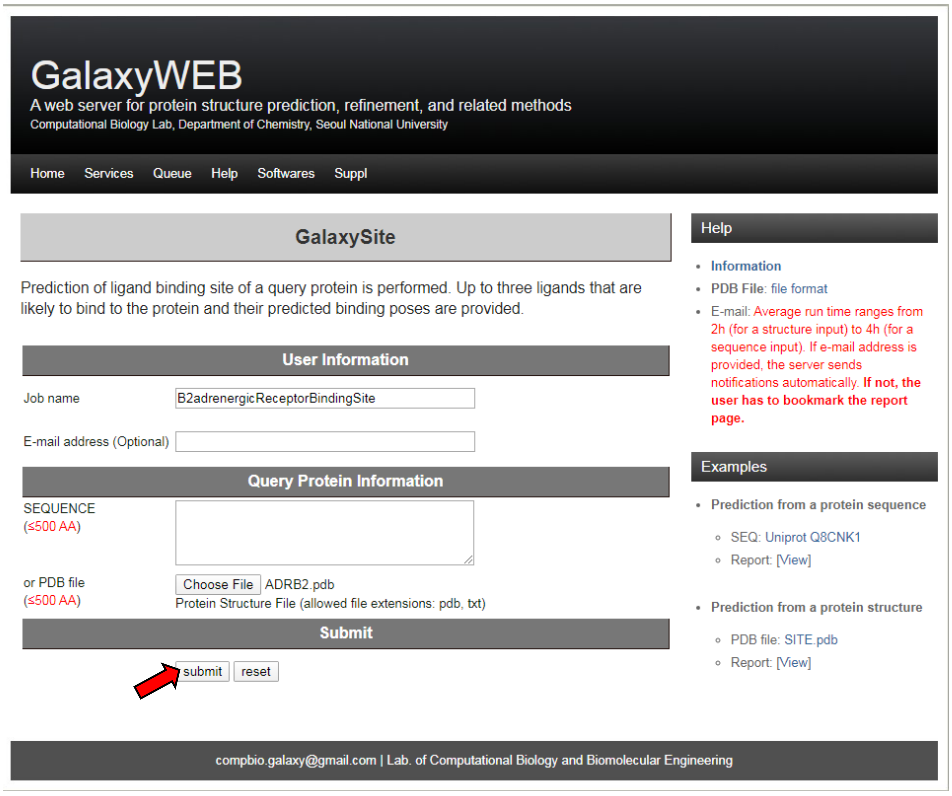

Once GalaxySite finishes the calculations, the page refreshes and includes the results. A number of high affinity ligands are also provided, together with the template molecule used for the computation of the receptor poses. Both solutions for the orthosteric and possible allosteric grooves are provided. Data/models (in pdb format) can be downloaded.

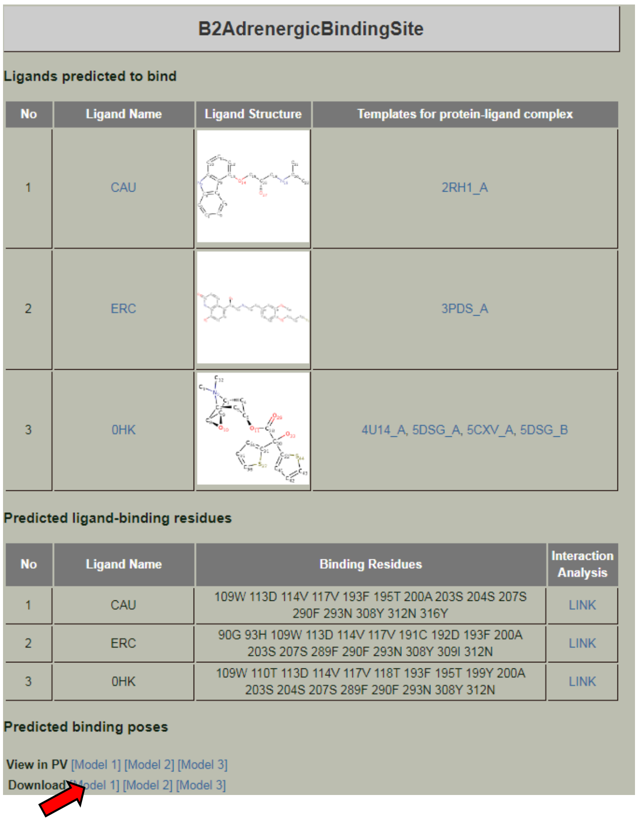

After the identification of the binding Site, perform the flexible docking experiment using the Galaxy-7TM service:

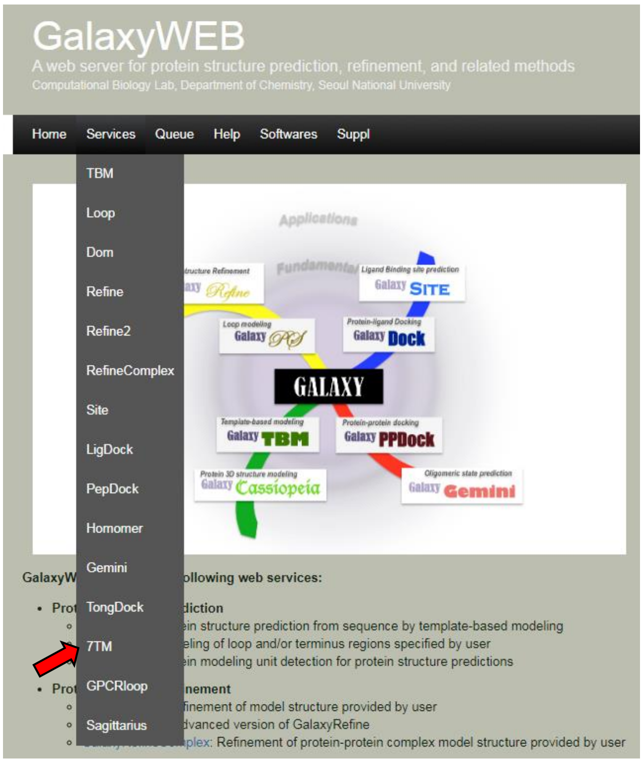

In the Galaxy7TM page service, fill the details of the receptor and the ligand:

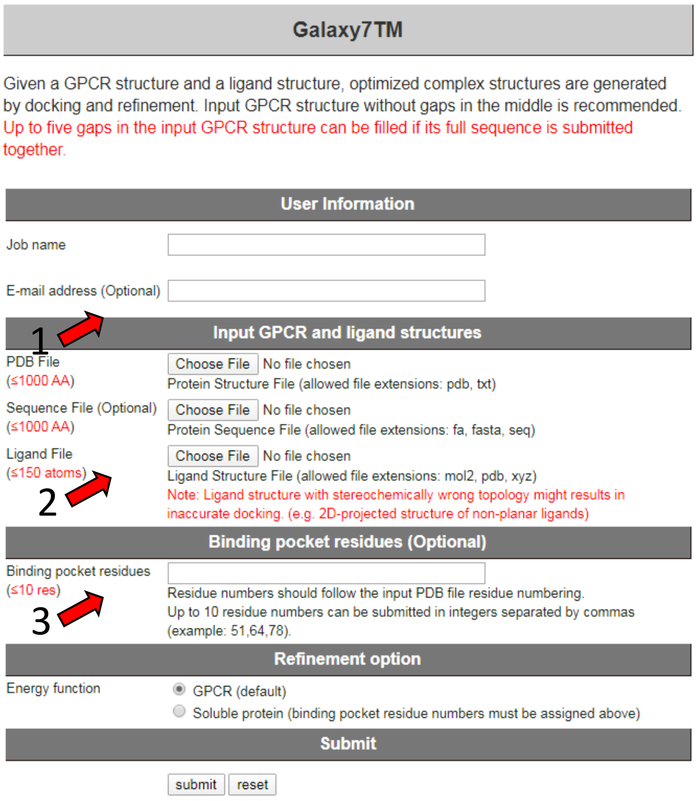

and then submit the job:

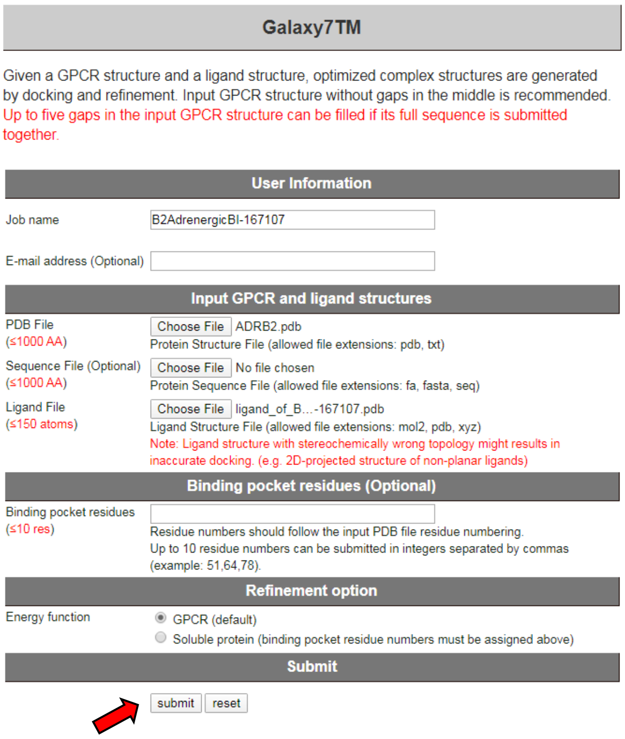

Once Galaxy7TM finishes the calculations, the page refreshes and includes the results. One can download the models:

ADRB2_BI-167107

View in PV [1] [2] [3] [4] [5] [6] [7] [8] [9] [10] [All Models]

Download [1] [2] [3] [4] [5] [6] [7] [8] [9] [10] [All Models]

Model Information

Model	Refinement energy	Docking energy	Ligand RMSD from Model 1	Binding site interactions	Residues in contact
MODEL 1	-17929.648	-13.302	0.000	LINK	LINK
MODEL 2	-17915.544	-13.688	8.512	LINK	LINK
MODEL 3	-17907.180	-14.280	1.860	LINK	LINK
MODEL 4	-17919.192	-13.169	8.402	LINK	LINK
MODEL 5	-17892.591	-13.408	8.490	LINK	LINK
MODEL 6	-17931.349	-12.952	1.654	LINK	LINK
MODEL 7	-17906.680	-13.106	8.456	LINK	LINK
MODEL 8	-17888.649	-13.433	3.228	LINK	LINK
MODEL 9	-17876.625	-14.328	8.229	LINK	LINK
MODEL 10	-17900.008	-13.062	7.237	LINK	LINK

We can see the binding site interactions:

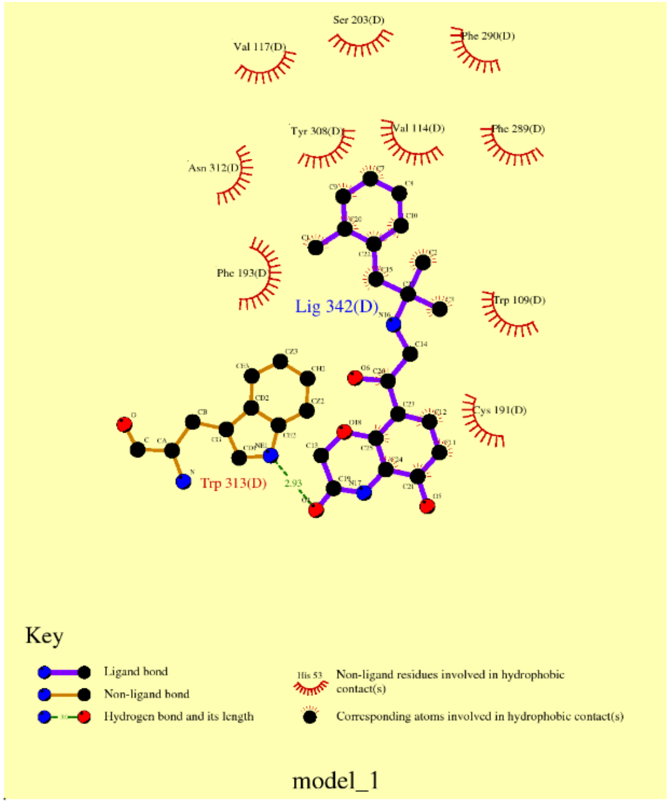

and the residues in contact:

```
RES    90  GLY
RES    93  HIS
RES    94  ILE
RES   109  TRP
RES   113  ASP
RES   114  VAL
RES   117  VAL
RES   191  CYS
RES   193  PHE
RES   203  SER
RES   207  SER
RES   289  PHE
RES   290  PHE
RES   293  ASN
RES   308  TYR
RES   309  ILE
RES   312  ASN
RES   313  TRP
RES   316  TYR
```

We can open the pdb file of the complex receptor-ligand using the Chimera program:

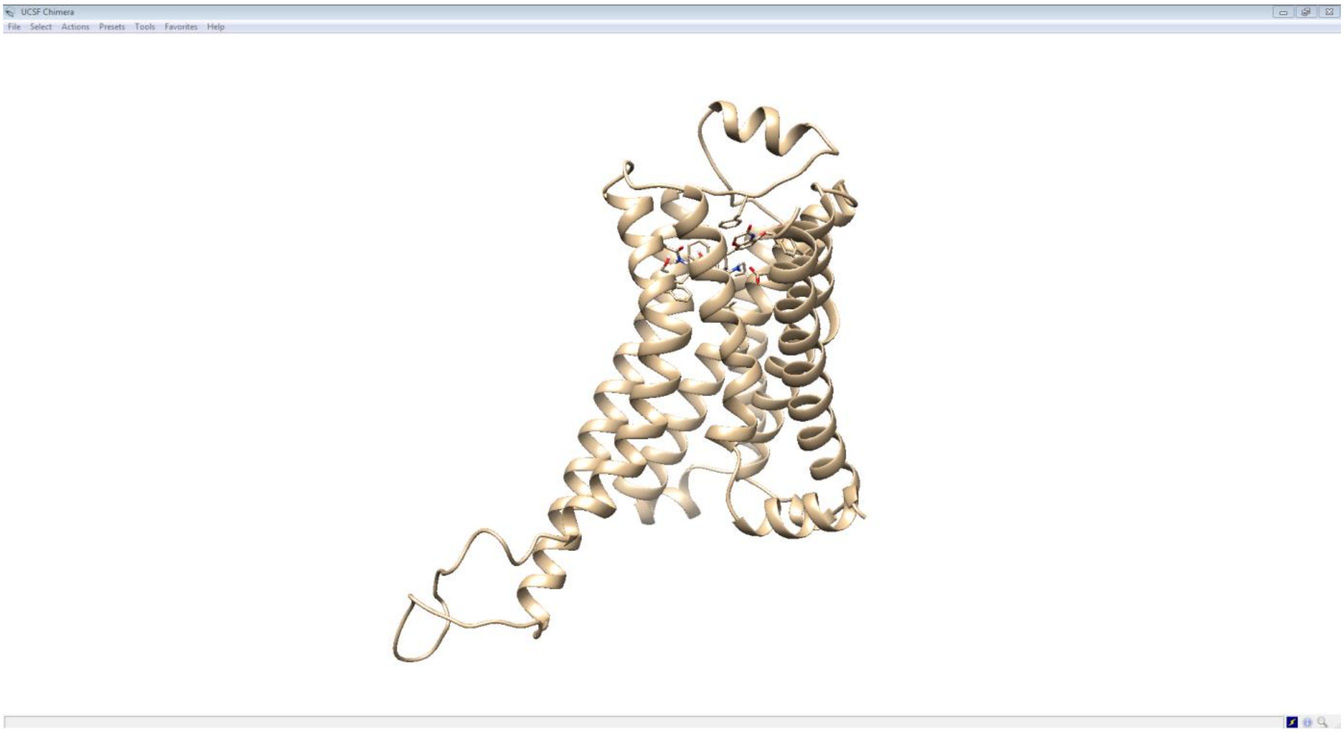

Ligand pose comparison

At this stage, one can open the pdb file and with a text editor keep only information concerning the ligand.

The same is also done on another pdb file (for example that of the crystal structure).

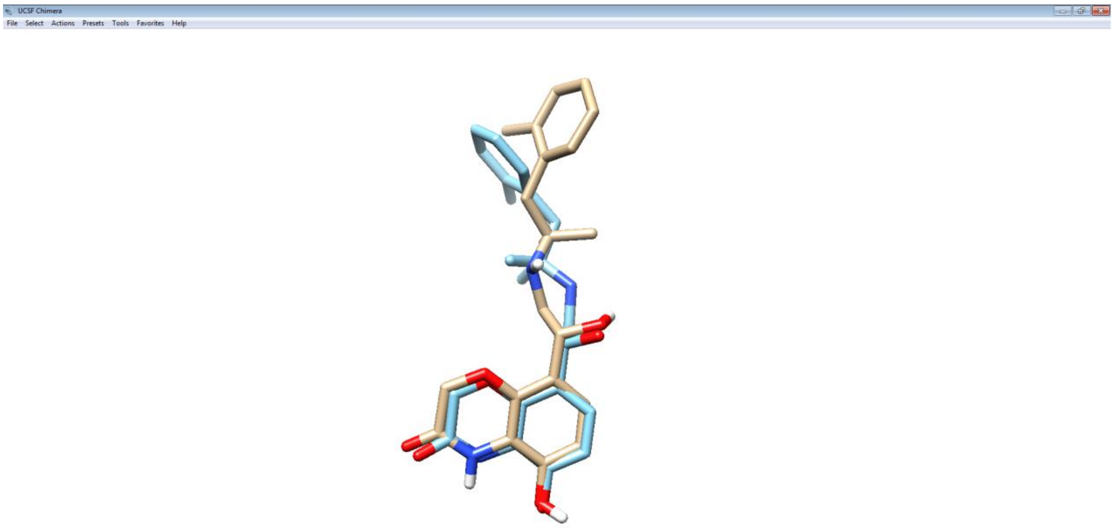

After that, we save the pdb file of the two ligand in this position.

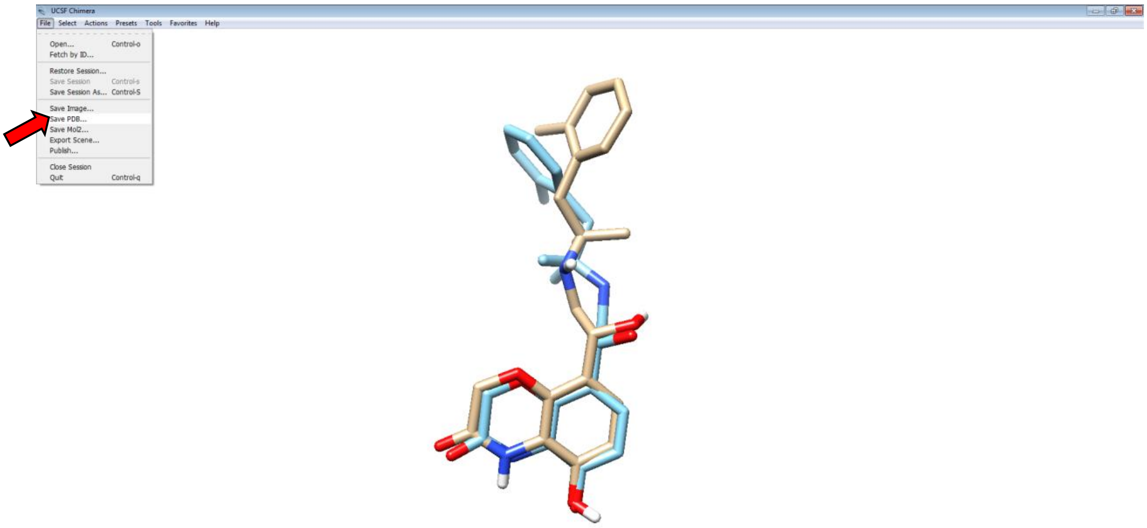

We open again the pdb file with the two ligand.

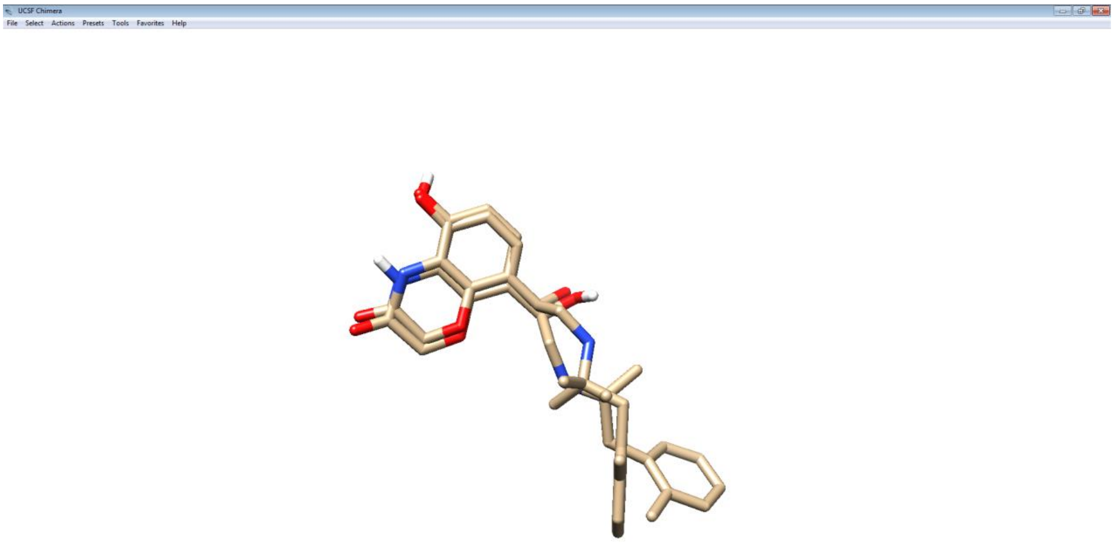

From the select panel,

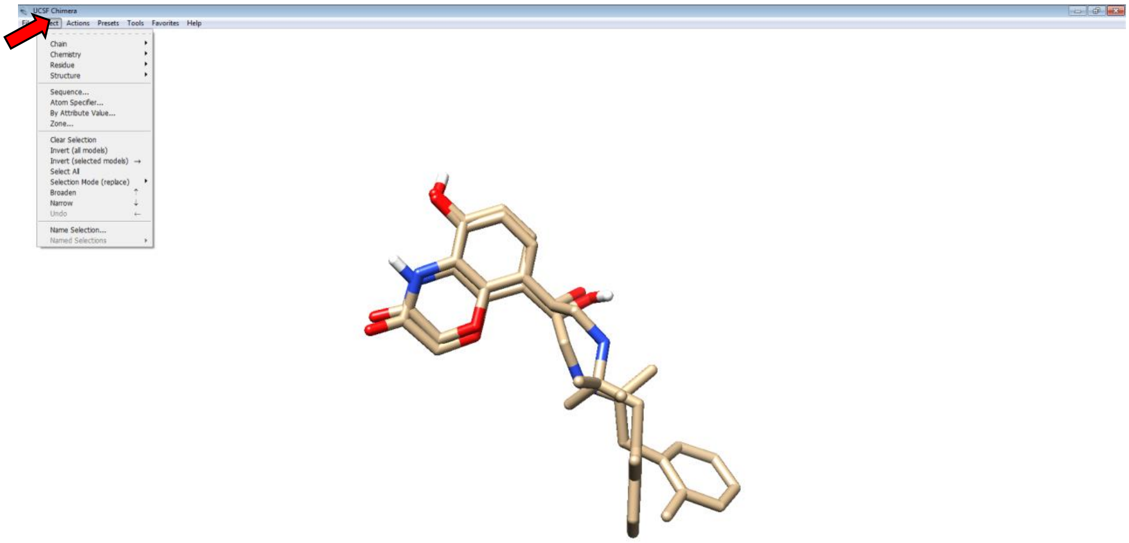

we select the one from two ligand from ‘’Chain, D or A’’,

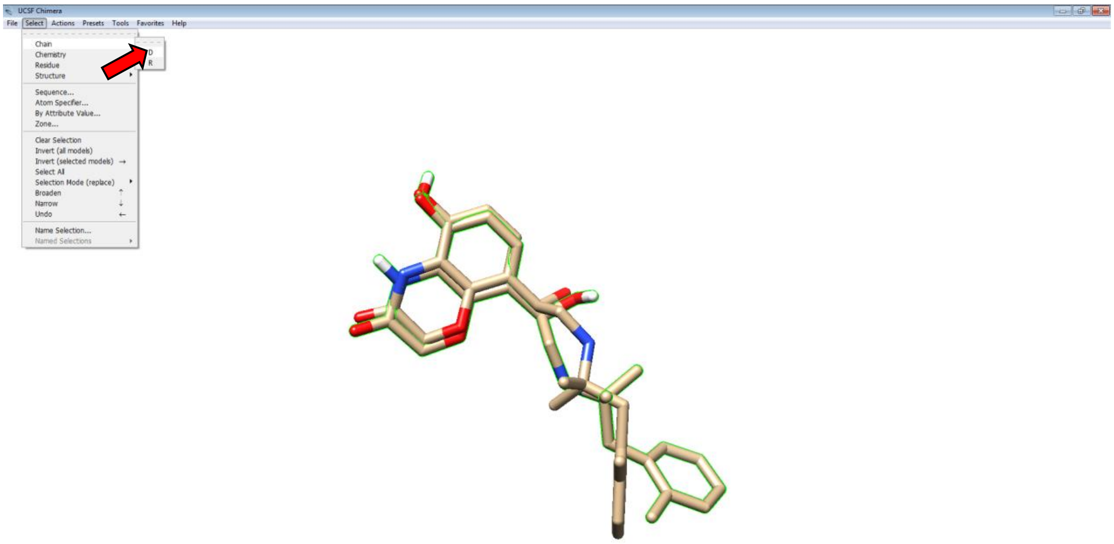

after that, we delete the ligand, from ‘’Actions’’,

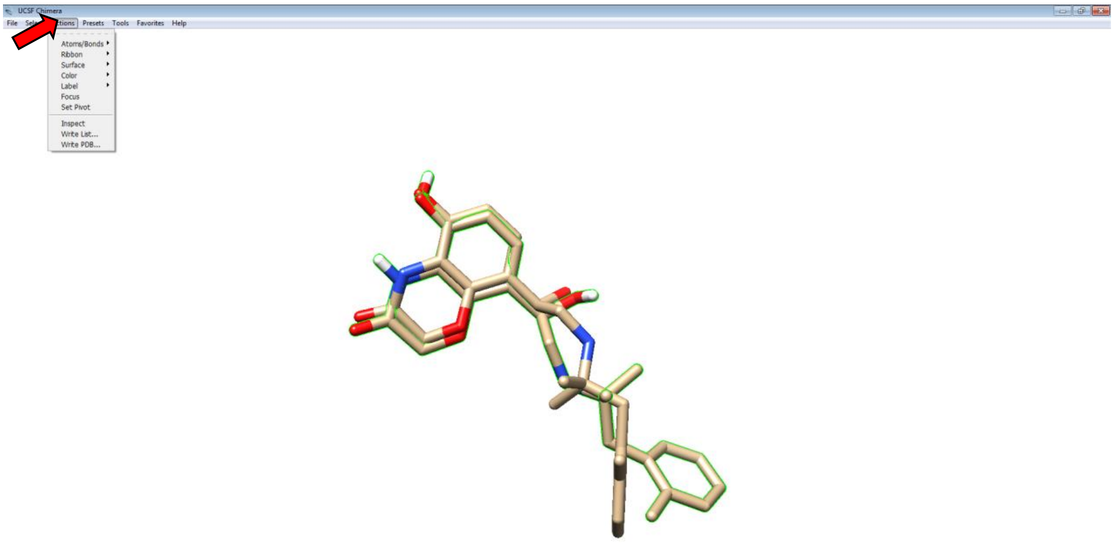

‘’Atoms/Bonds’’ and ‘’delete’’,

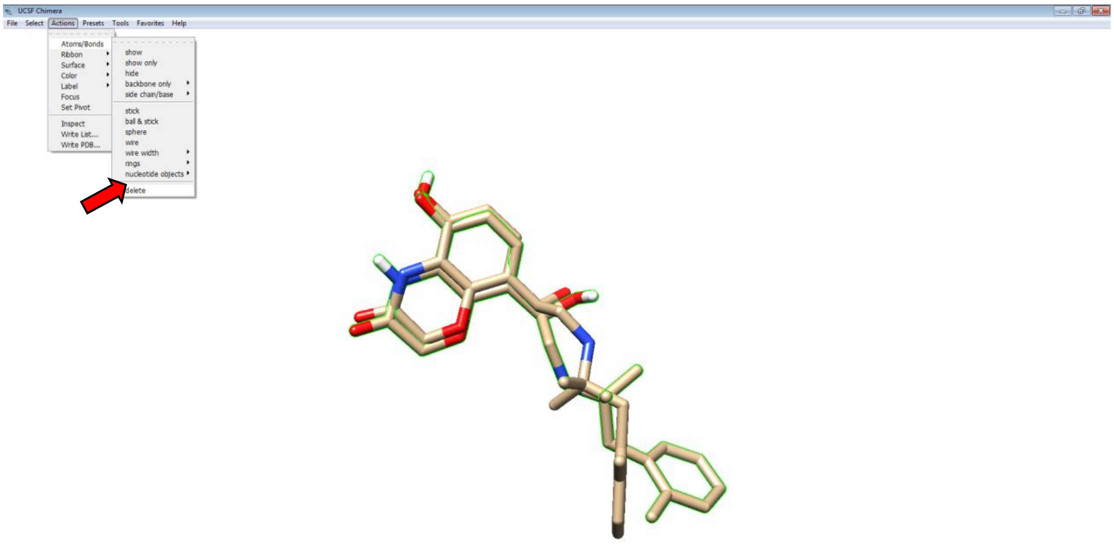

We save the remained ligand

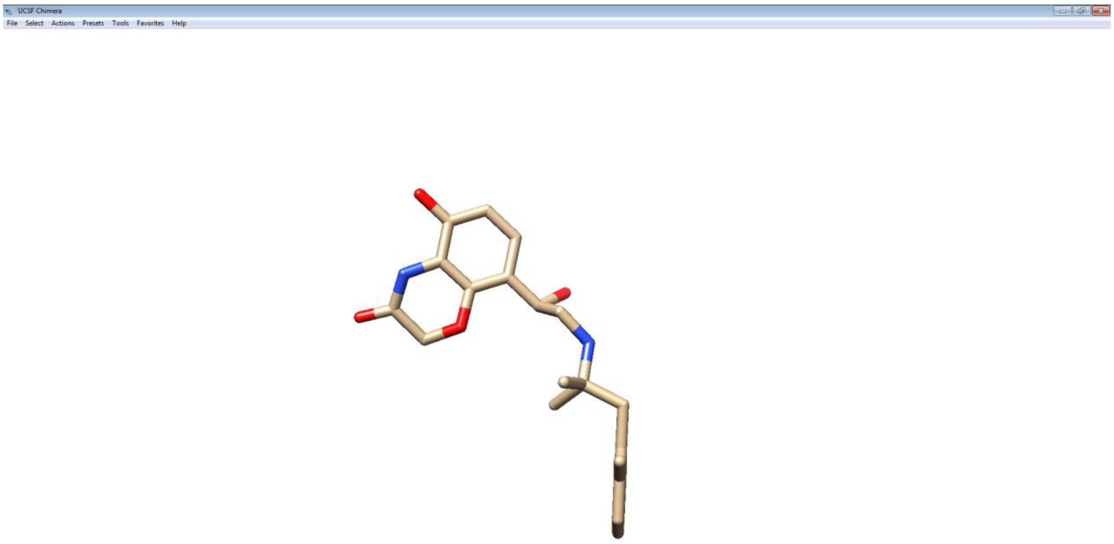

as mol2 format.

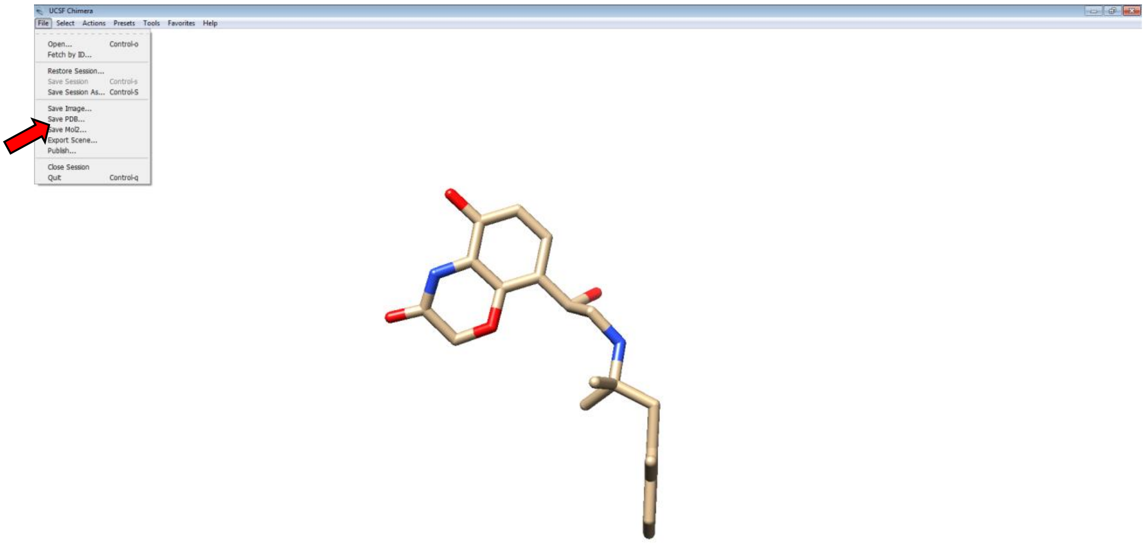

We repeat the same process for the second ligand.

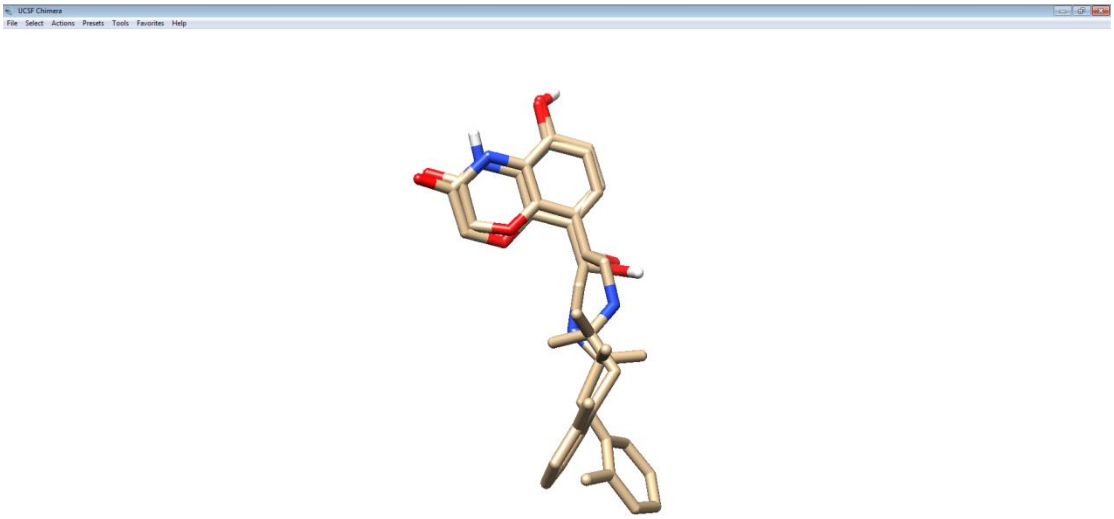

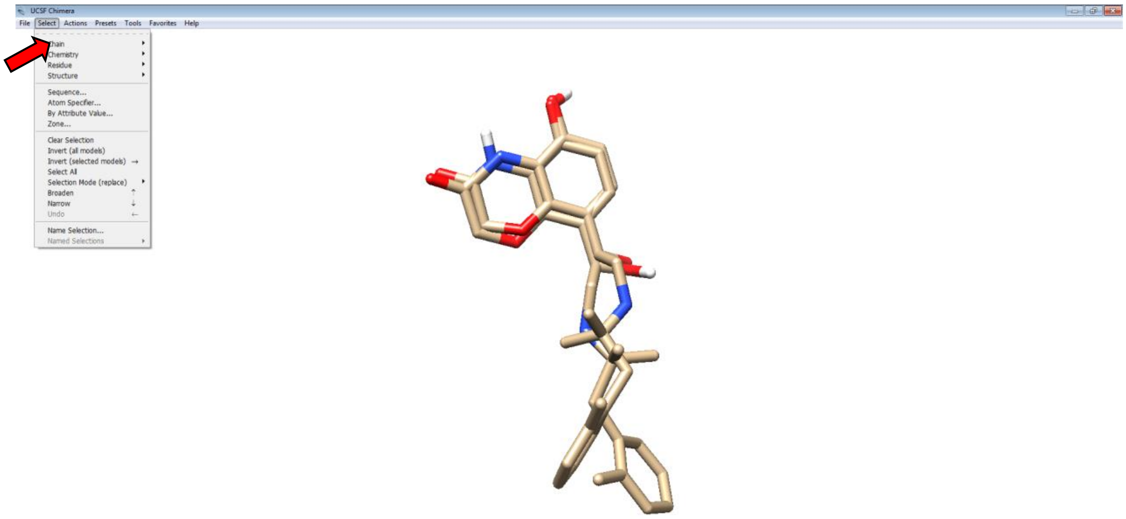

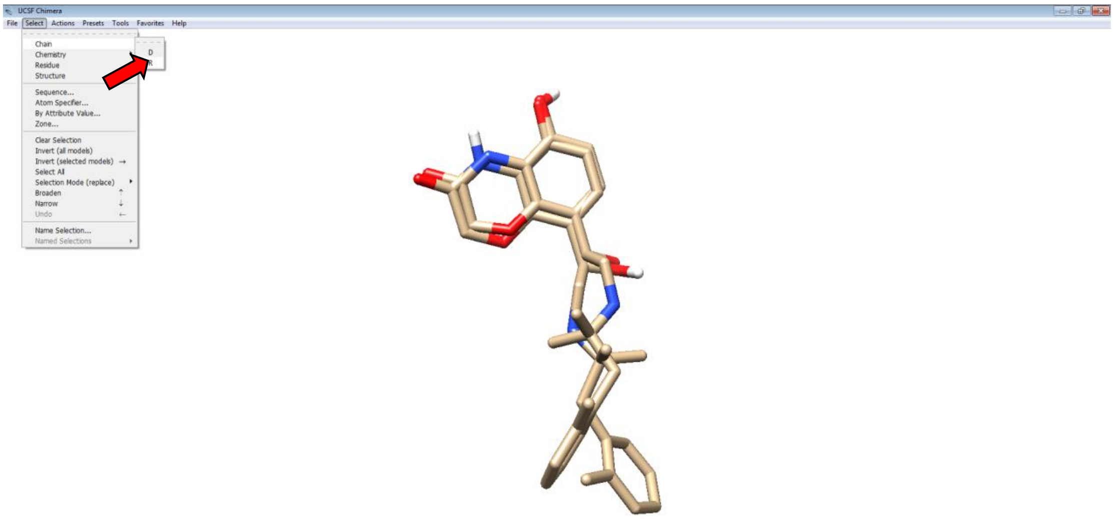
UCSF Chimera
File Select Actions Presets Tools Favorites Help
Chain
Chemistry
Residue
Structure
Sequence...
Atom Specifier...
By Attribute Value...
Zone...
Clear Selection
Invert (all models)
Invert (selected models)
Select All
Selection Mode (replace)
Broaden
Narrow
Undo
Name Selection...
Named Selections
D

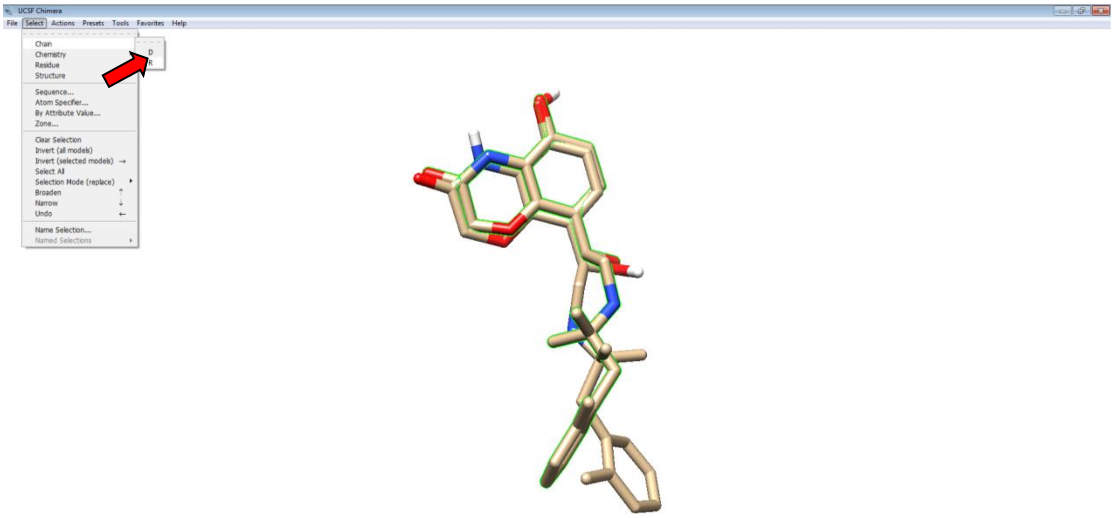
UCSF Chimera
File Select Actions Presets Tools Favorites Help
Chain
Chemistry
Residue
Structure
Sequence...
Atom Specifier...
By Attribute Value...
Zone...
Clear Selection
Invert (all models)
Invert (selected models)
Select All
Selection Mode (replace)
Broaden
Narrow
Undo
Name Selection...
Named Selections
D

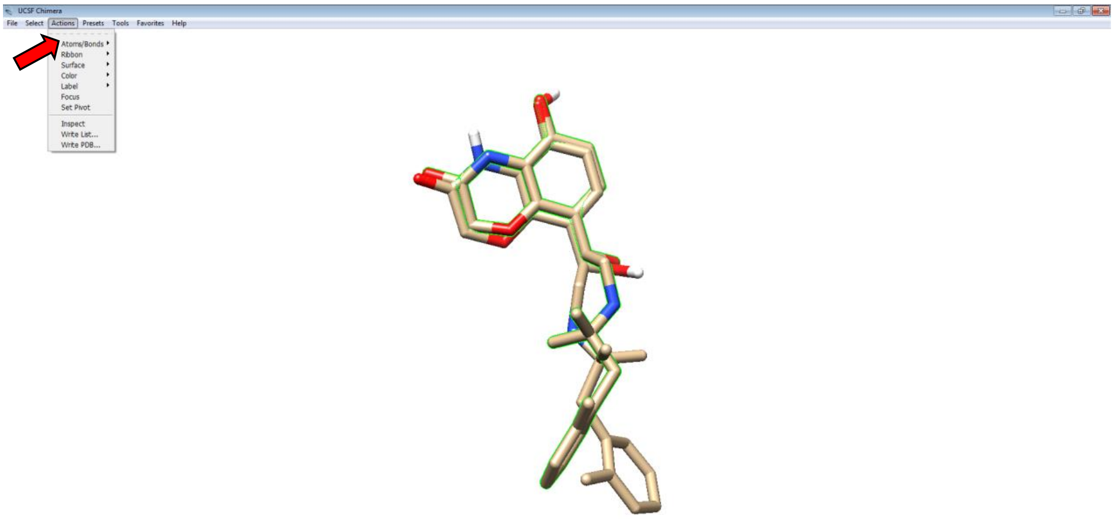
UCSF Chimera
File Select Actions Presets Tools Favorites Help
Atoms/Bonds
Ribbon
Surface
Color
Label
Focus
Set Pivot
Inspect
Write List...
Write PDB...

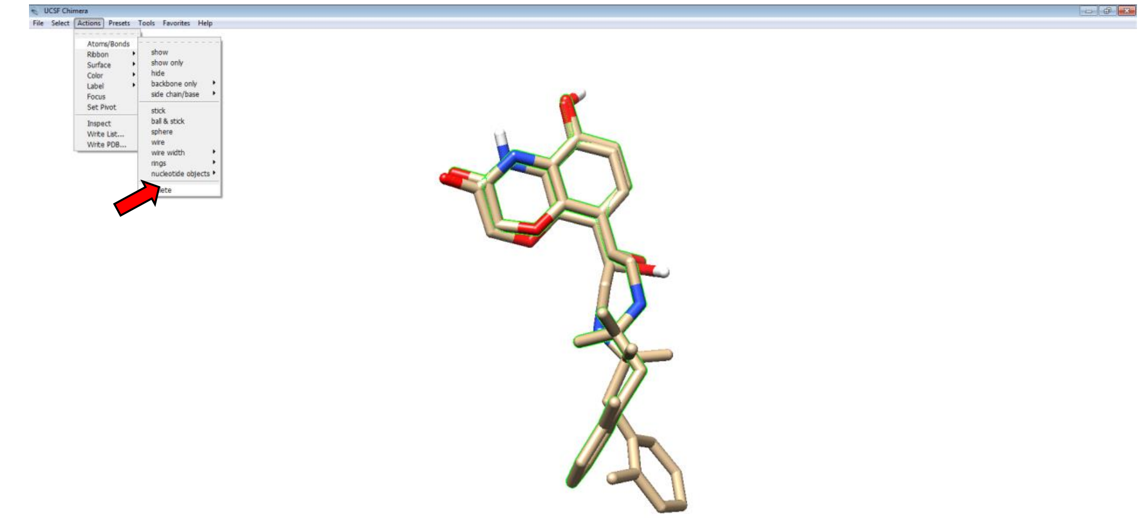

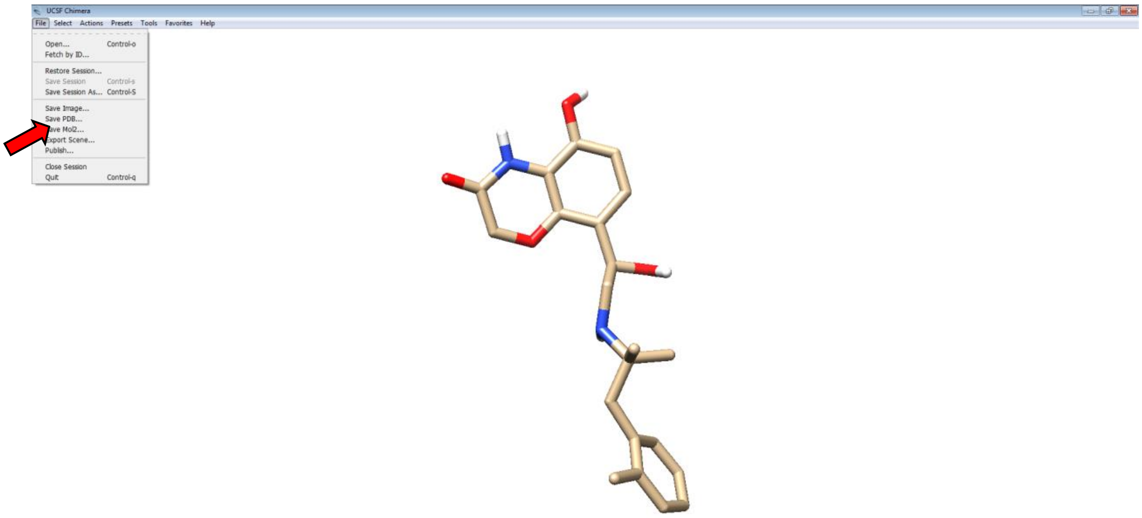

We open together the two ligands with the Chimera program.

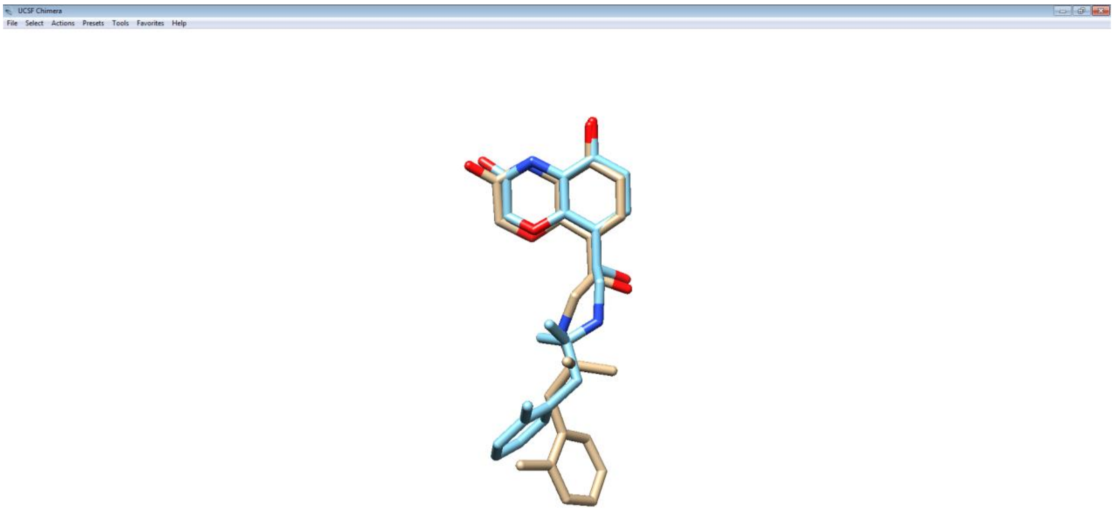

In the panel of ‘’Favorites’’, we select ‘’Command Line’’,

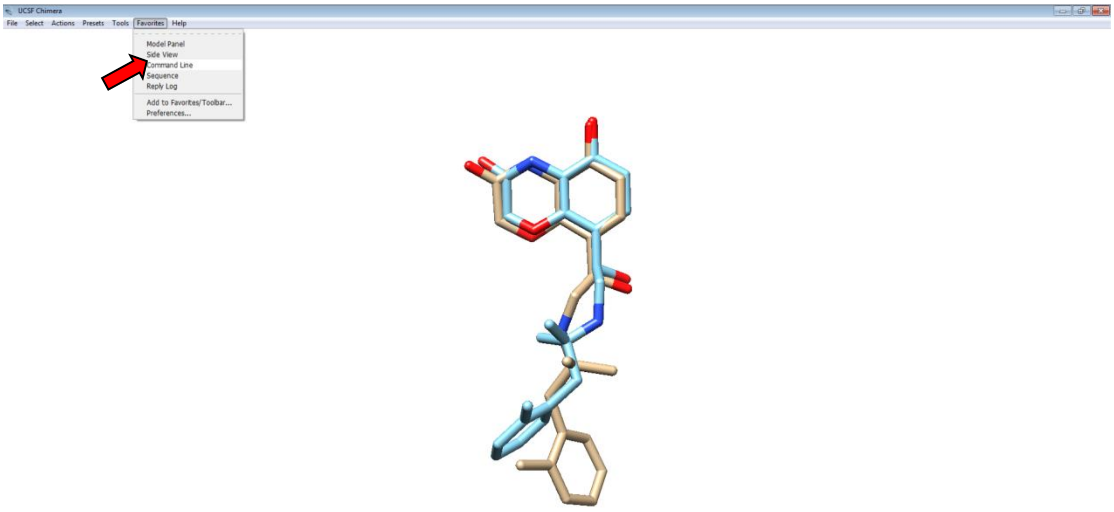

at the command line, we write ‘’rmsd#0: 1#’’ and click ‘’Enter’’. The RMSD value appear in the command line window.

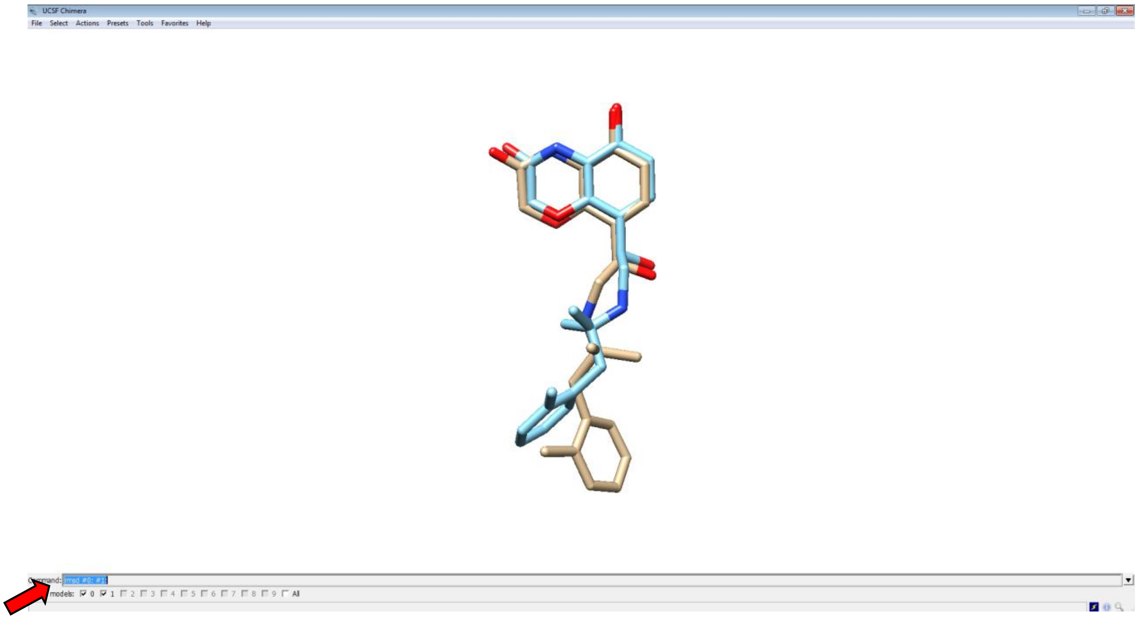

Alternative, we open together the two ligands with pyMOL program.

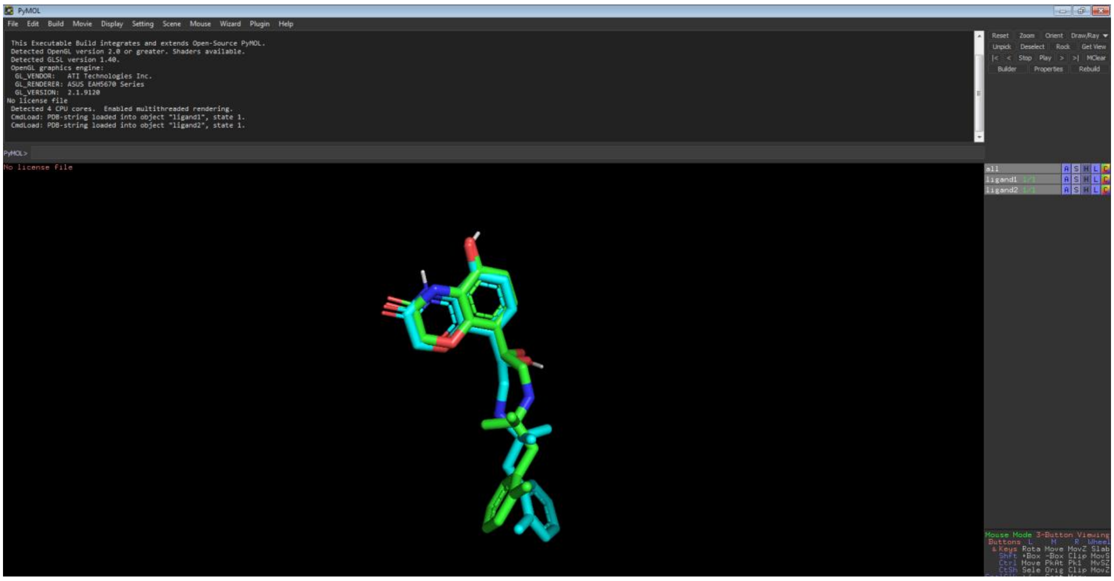

From the Plugin panel we select to ''Initialize Plugin System'',

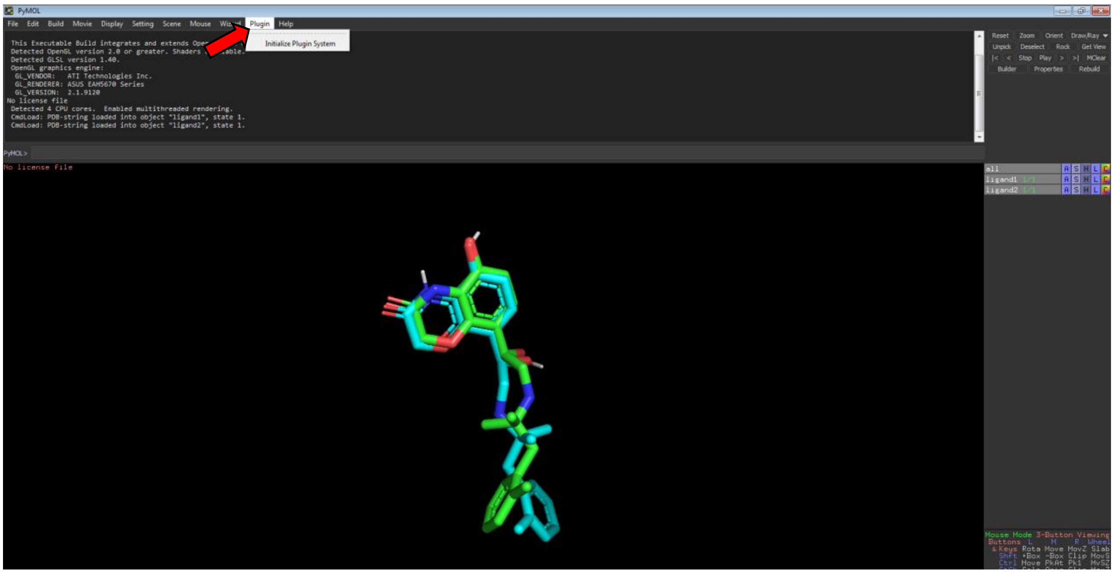

After that, we select the option for ‘’Aligment/Superposition’’,

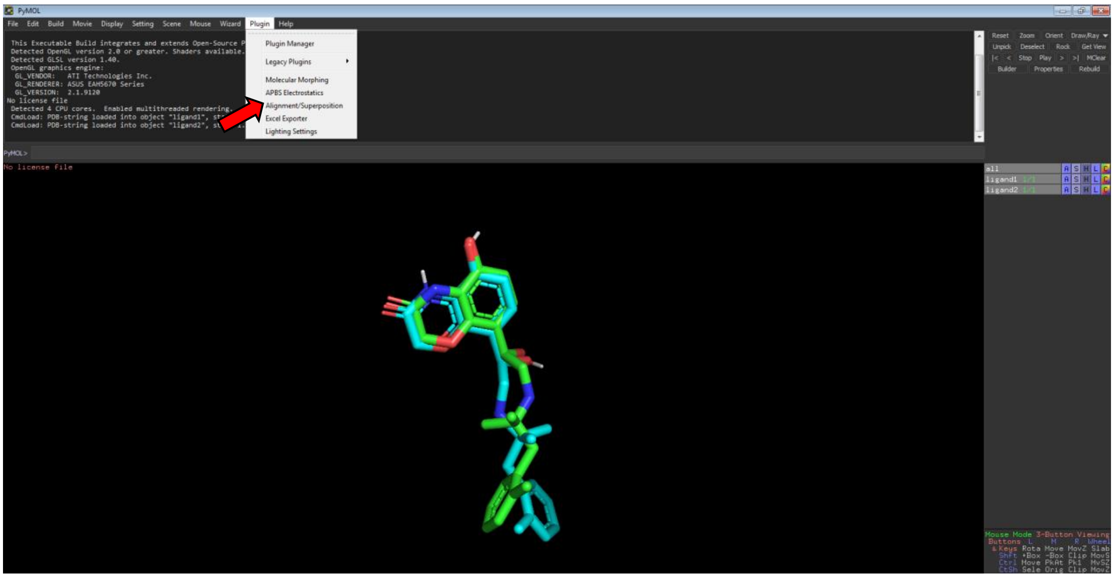

In the new window, se select the ‘’ok’’ option for align of two ligands and the RMSD value appear in the command line window.

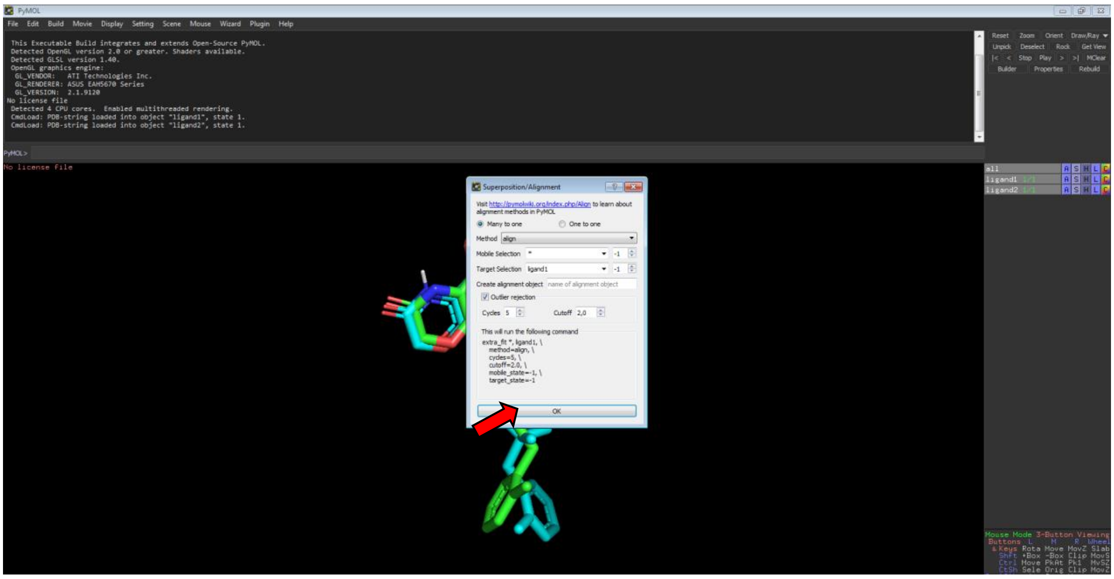

G-protein interactions

A subsequent step of GPCR-ligand activation is the binding with G-proteins, as specific sites. More specifically, G_{α} is bound to intracellular activated receptor loops 2 and 3, and $G_{\beta,\gamma}$ is bound to intracellular chain 8, initiating different intracellular signaling events. In this work, we have examined the interaction of GPCRs with G_{α} proteins. It is to note that, after binding of G_{α} protein (bound to GDP and denoted as Gα-GDP), the nucleotide is exchanged after receptor activation to GTP (Gα-GTP), and the Gα-GTP protein is liberated and subsequently triggers specific signaling pathways. Here, we explored the interaction of known and novel GPCRs with Gα proteins.

Gα protein model construction

At a first step, we have retrieved the sequences of G_{α}-proteins, in fasta format from the NCBI protein database (see the previous chapter about the receptor preparation). The Gα sequence is introduced in the GalaxyWeb server (module Refine) to generate and refine the 3D structures. This step was necessary, as the reported crystal structures of the different G_{α} molecules present significant gaps. Model structures, generated at the previous step can be refined, through the module "Refine2".

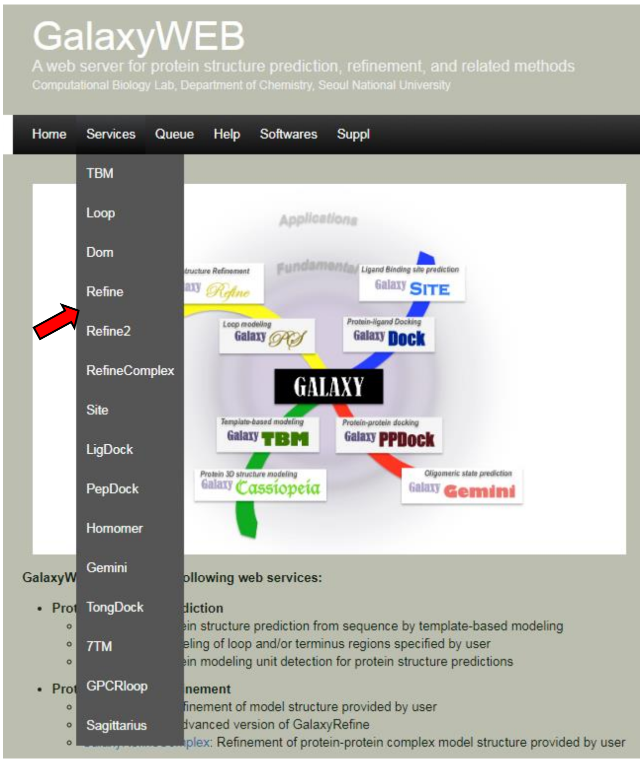

In the GalaxyRefine window, we fill the Job name, we choose the pdb file,

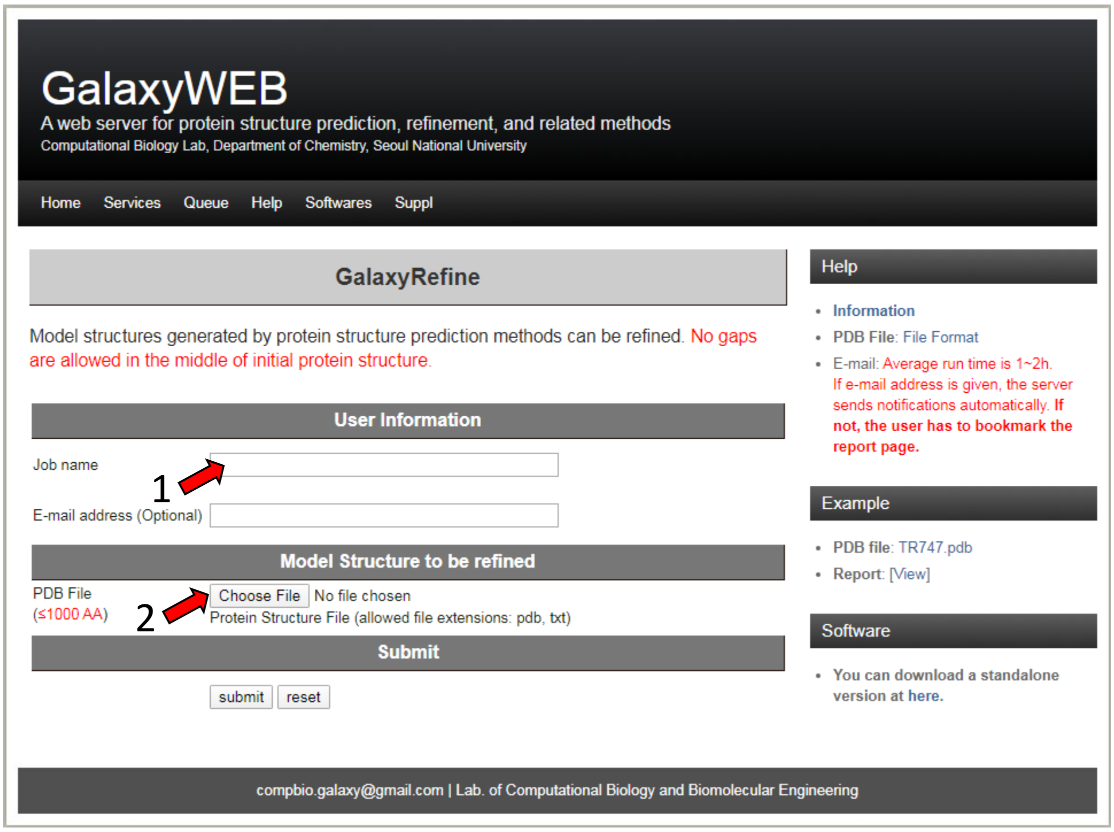

and finally, we submit the job.

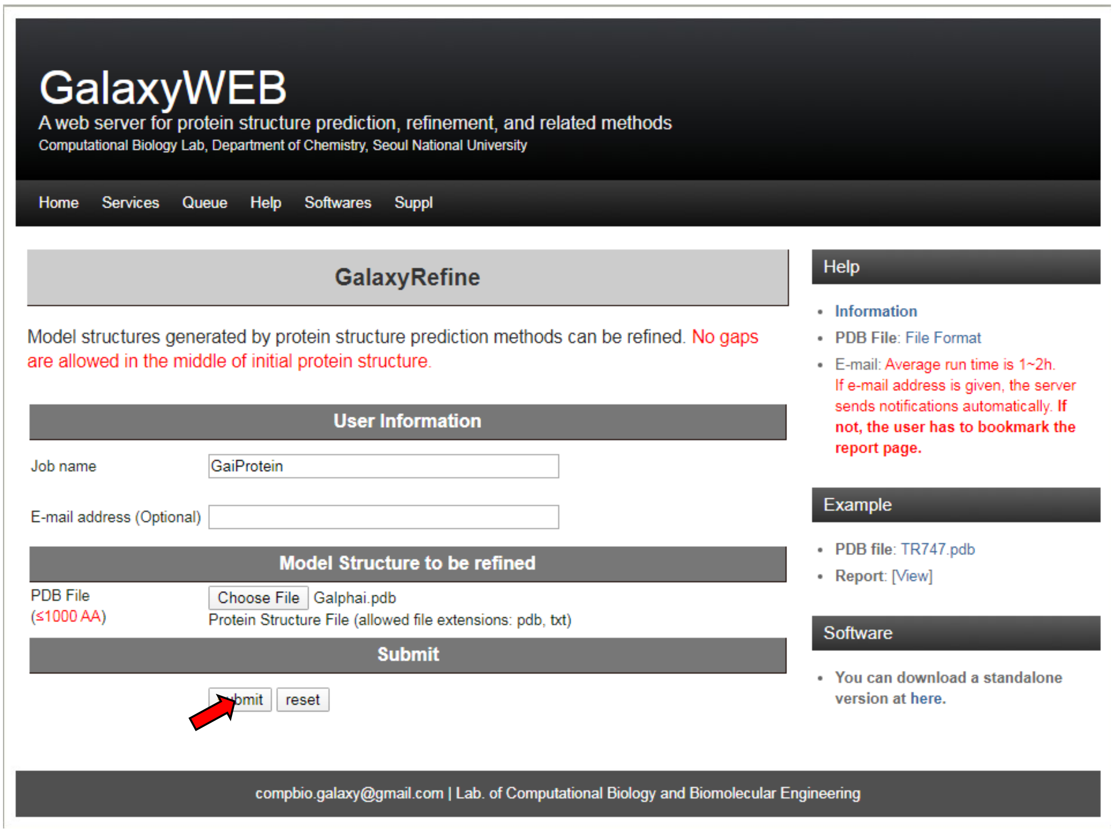

Binding of Ga proteins with GDP or GTP

The retrieved structures of G_{α}-proteins were then docked with GDP or GTP in a fully flexible on-the-fly method, in the GalaxyWEB server (Module LigDock).

For this reson, in GalaxyWEB Server, we select the LigDock service.

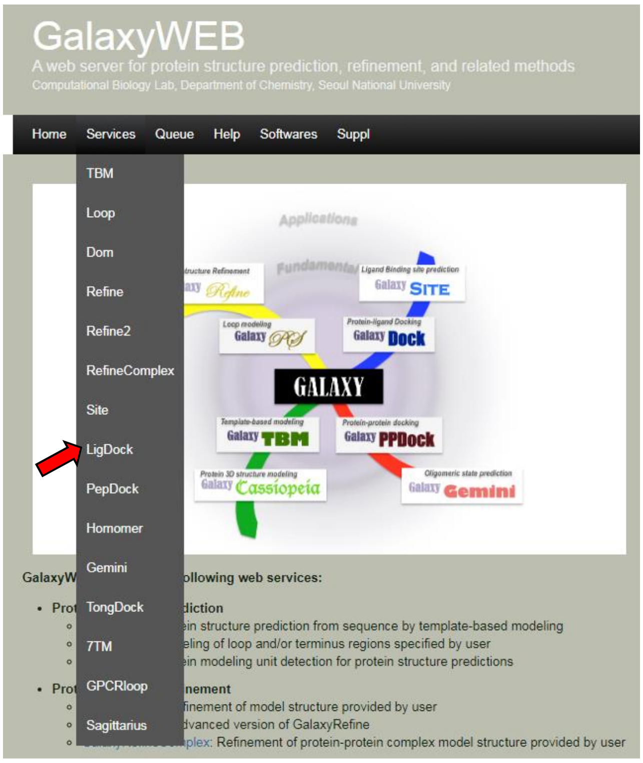

In the GalaxyDockWEB window, we fill the Job name, we choose the pdb file of protein and pdb file of ligand,

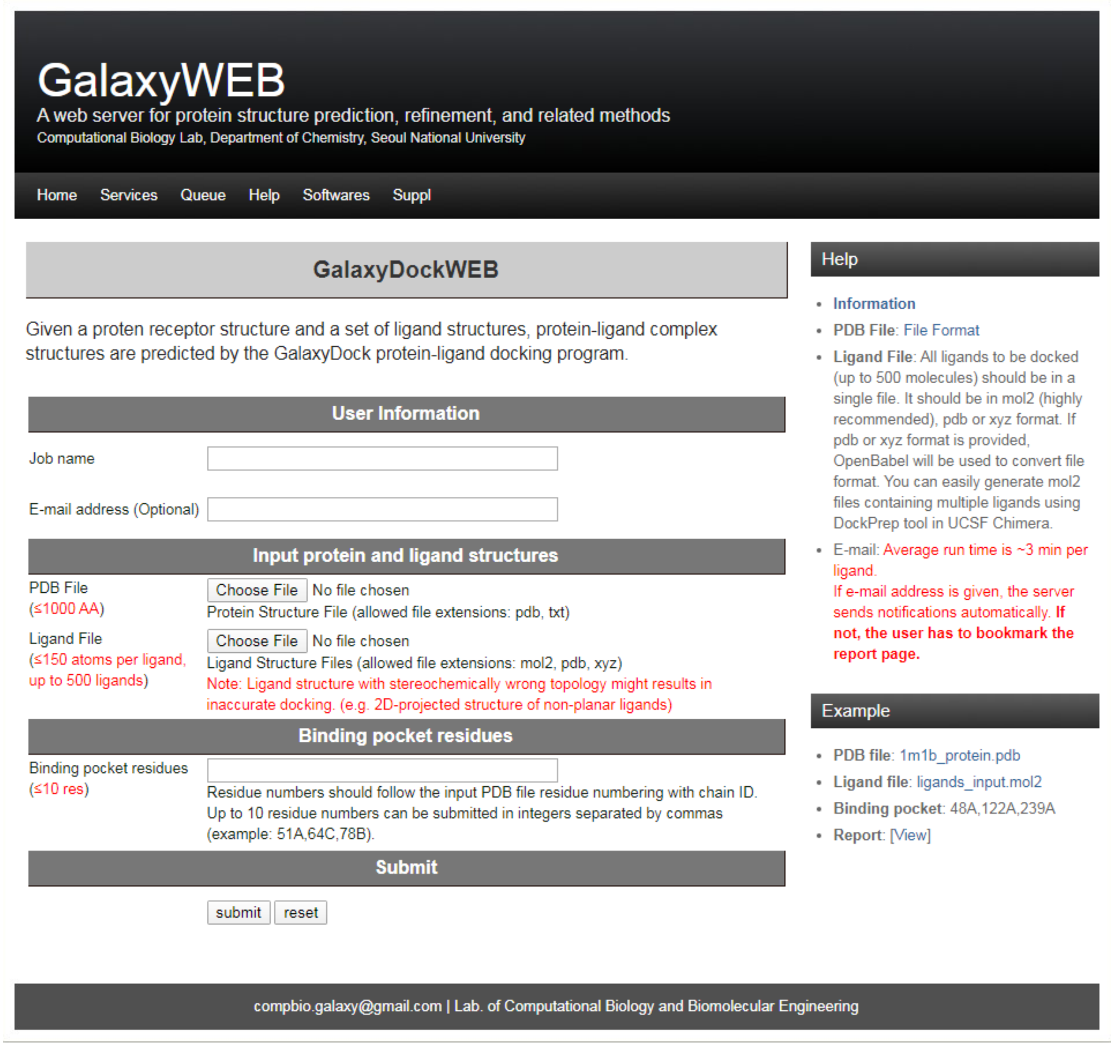

and finally, we submit the job.

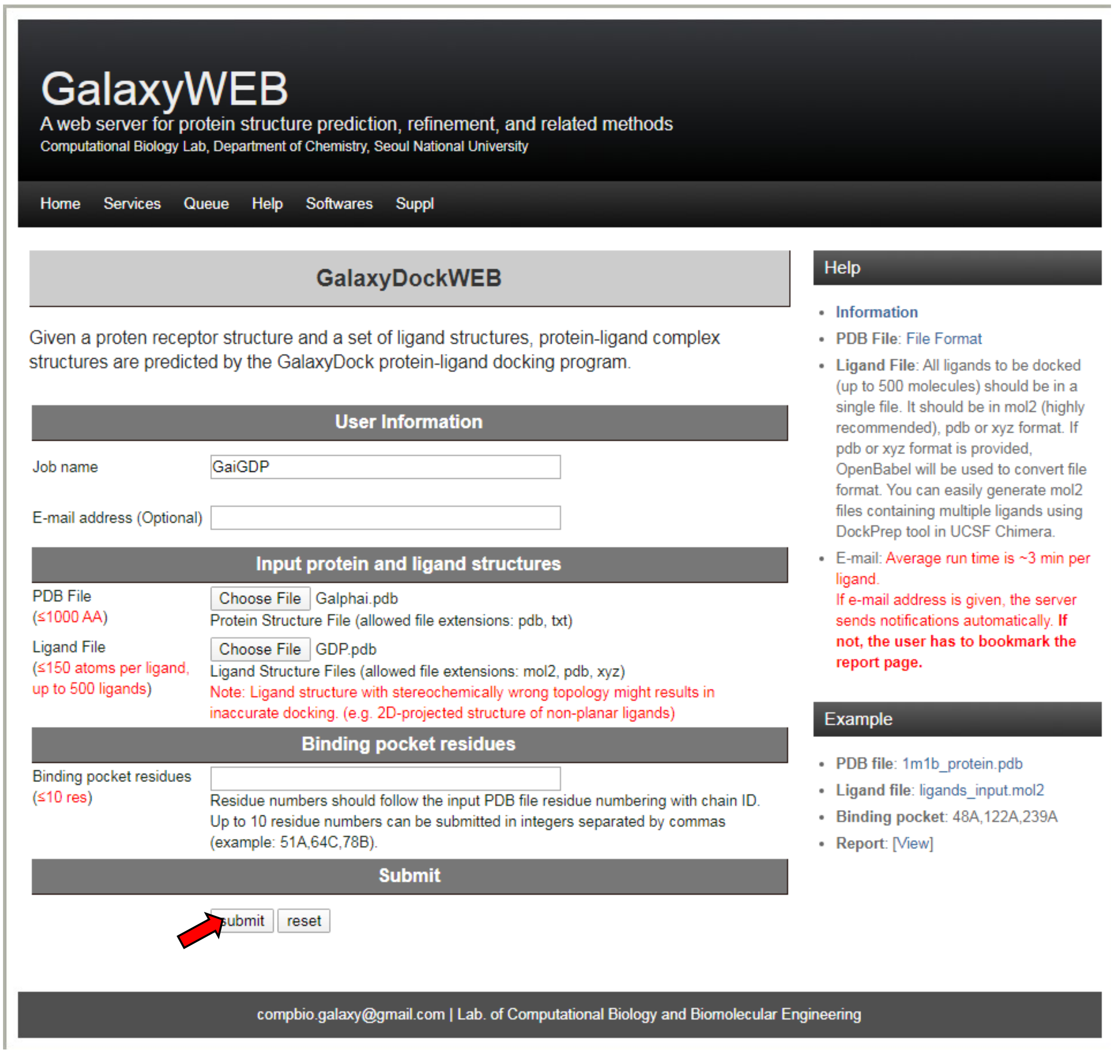

The liganded G_α pdb files are retrieved.

Binding of liganded receptor and liganded Gα protein

Protein-protein interaction servers do not permit the adequate solutions with large proteins with a high number of rotational bonds. Therefore, here, we have used the locally executed program Hex. This is an interactive molecular graphics program, which calculates and displays feasible docking modes of pairs of protein and/or DNA molecules, by performing spherical polar Fourier (SPF) correlations and exploring the similarities/complementarities of the two molecules, and provides through a graphical user interface, a set of >100 solutions, with the corresponding ΔG values (http://hex.loria.fr).

The files used are: (1) the ligand-receptor pdb file; (2) the G_α-GDP or Gα-GTP pdb files.

At a first step, we open the HEX 8.0.0 program:

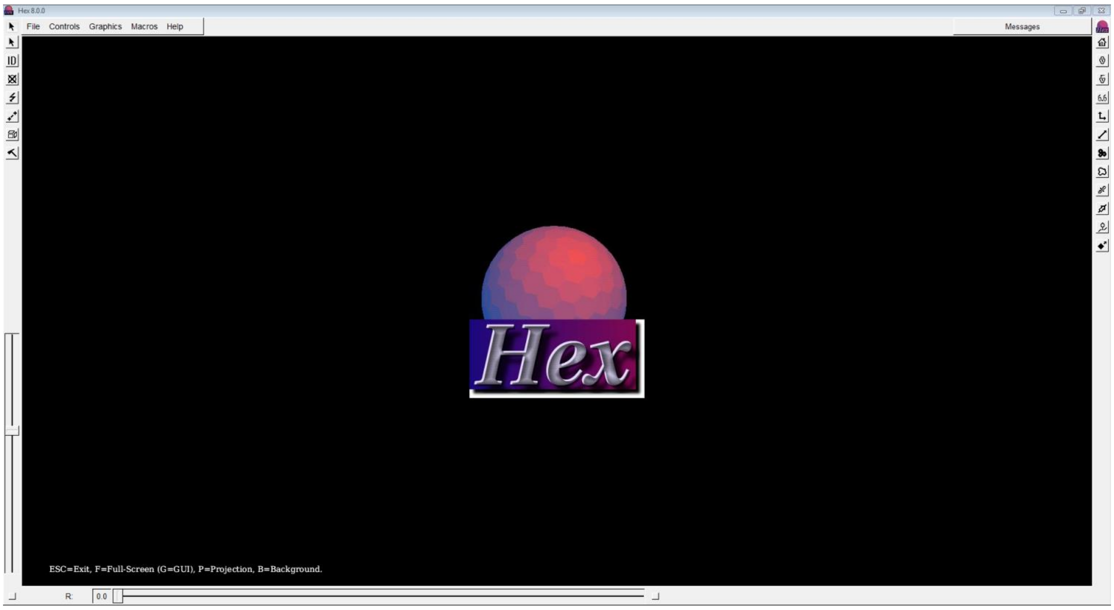

We open the receptor (complex of receptor-ligand) pdb:

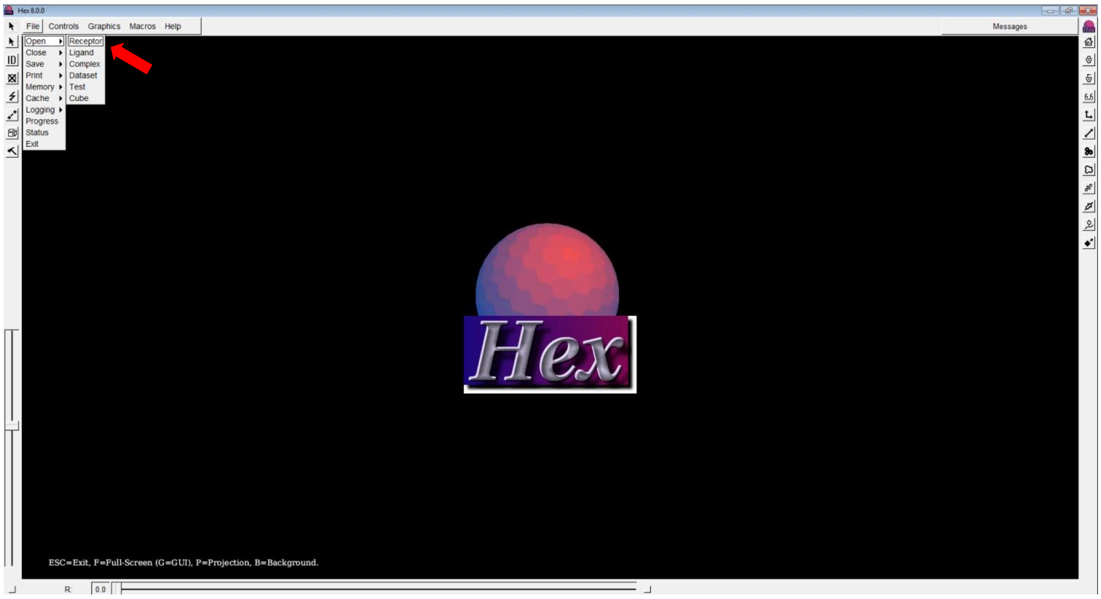

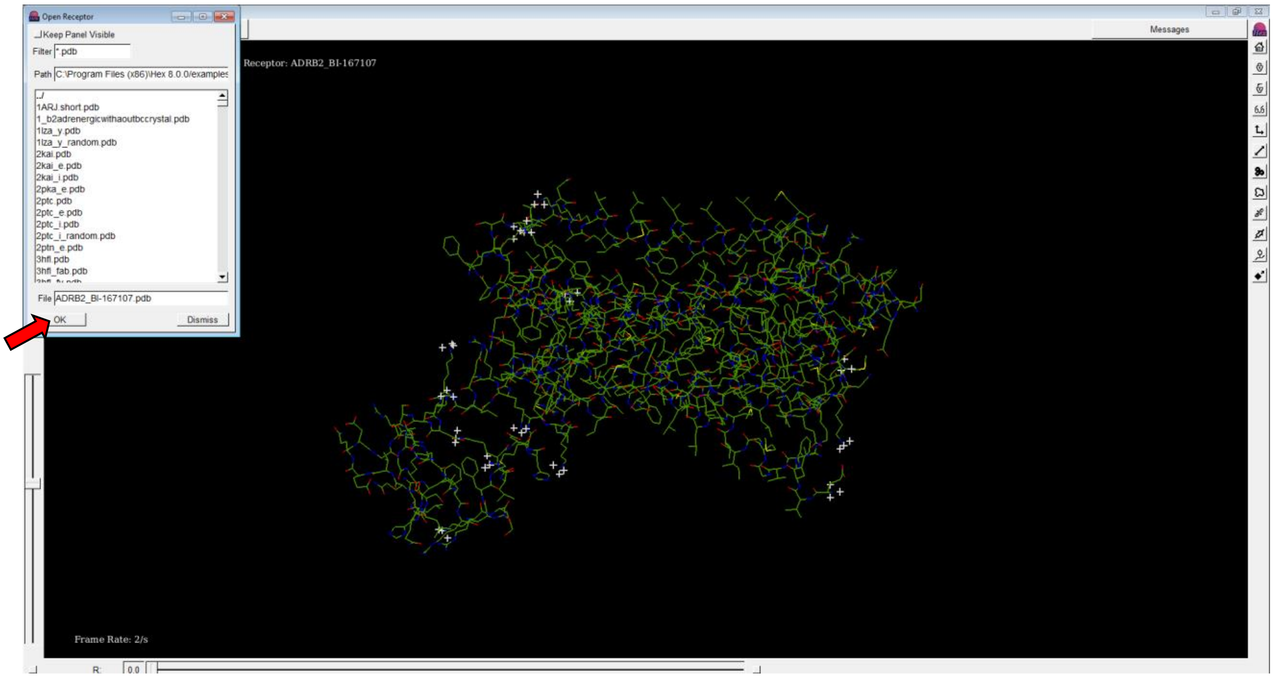

Then, we open the pdb file of Gα-protein (as ligand)

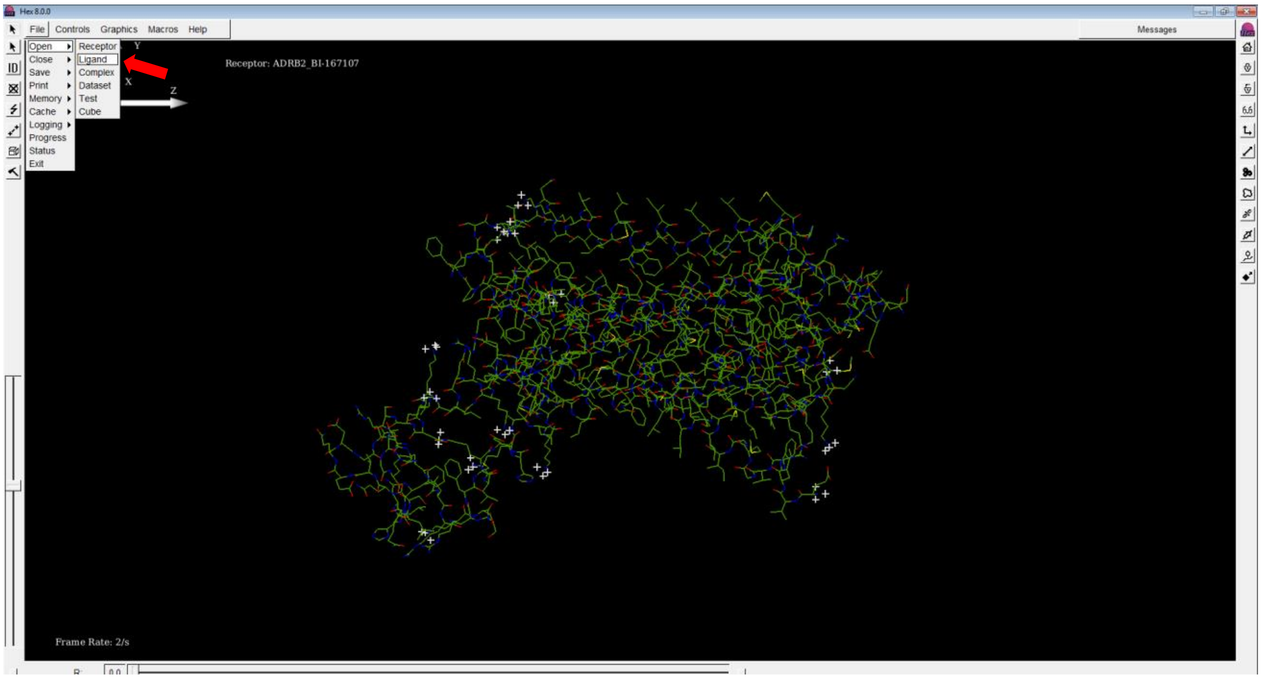

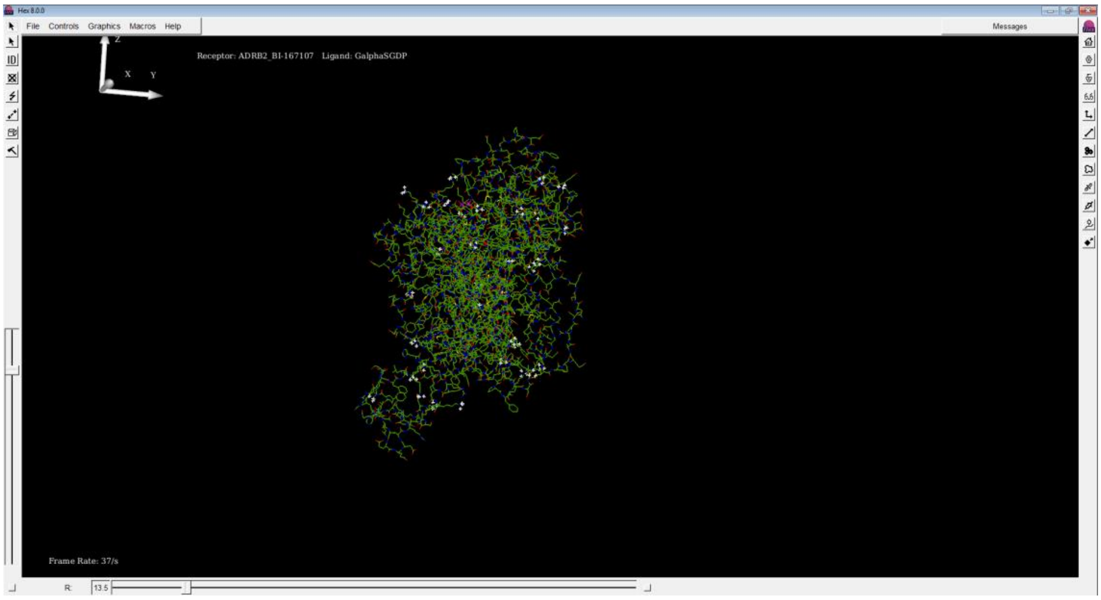

After this process, we select the docking properties from the control panel:

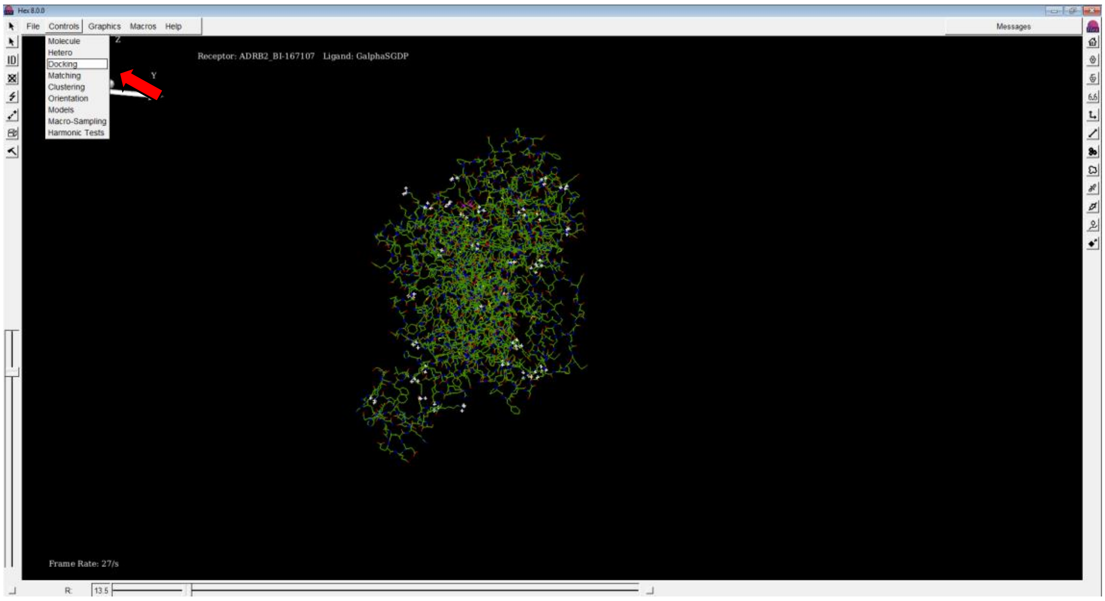

We change the correlation Type:

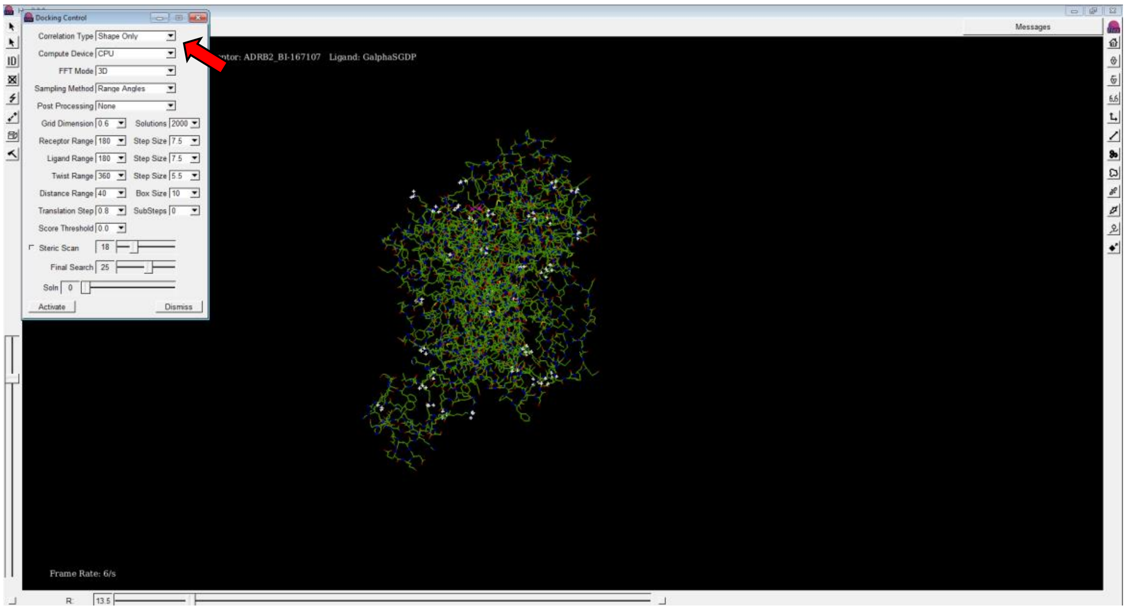

and we add <u>shape and Electro type</u>

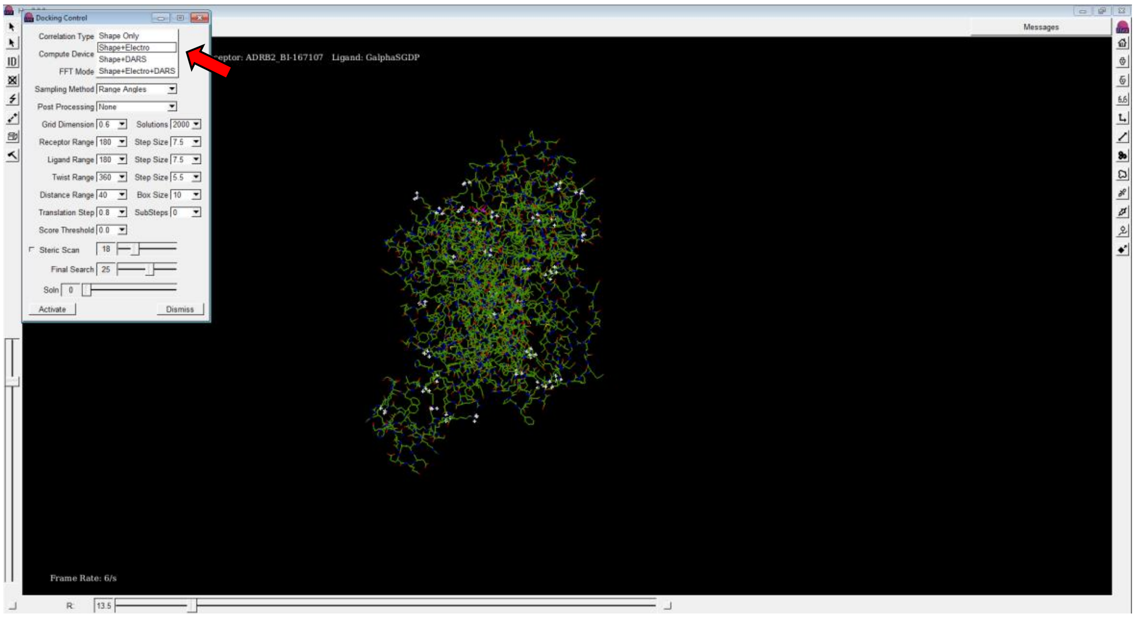

Then, we click on Activate to perform the docking experiment:

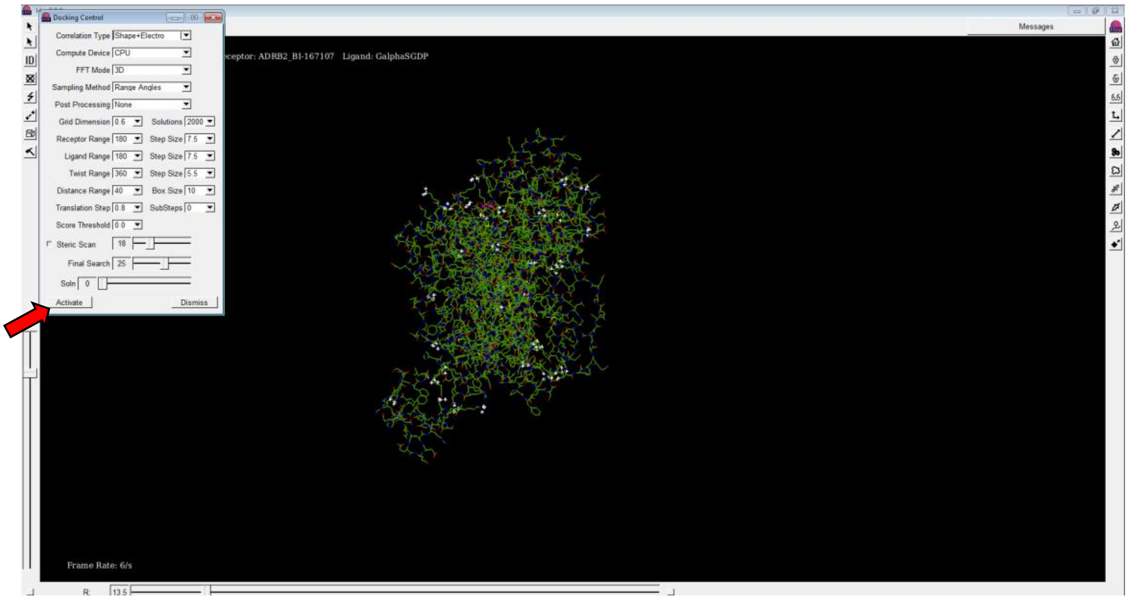

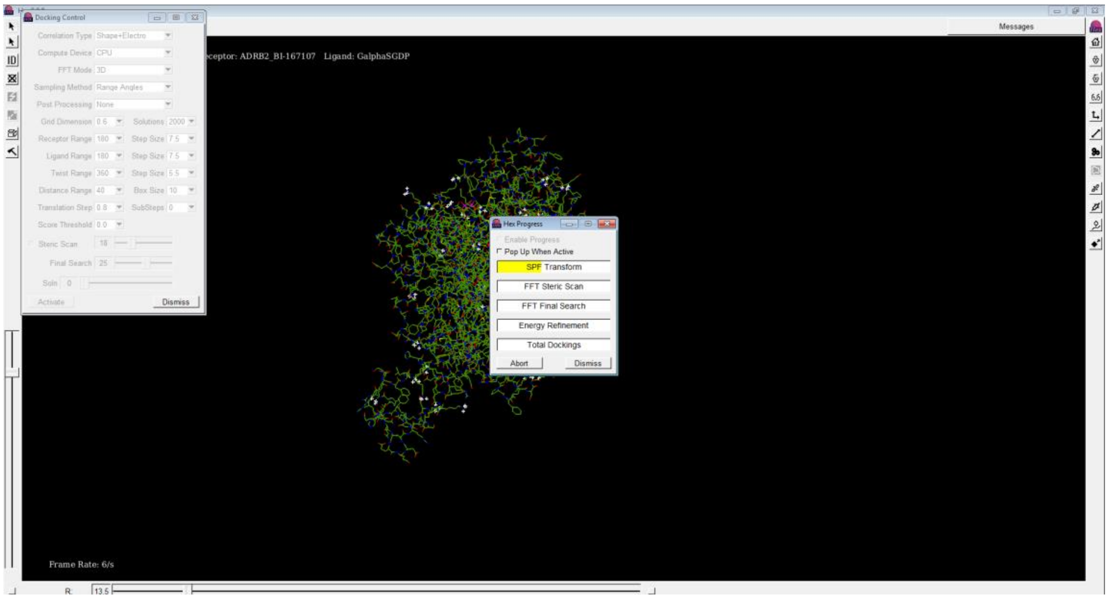

After completion, the program retains 100 models of protein-protein interactions, with the corresponding ΔG values.

It is to note that ΔGs are very high, as thay rely on all aminoacid interactions of two macromolecules. We scroll through the models and select select the best solution in which Gα binds to theintracellular loops 2 and 3 of the receptor. This solution (in pdb coordinates) is retrieved and inspected in the Chimera program.

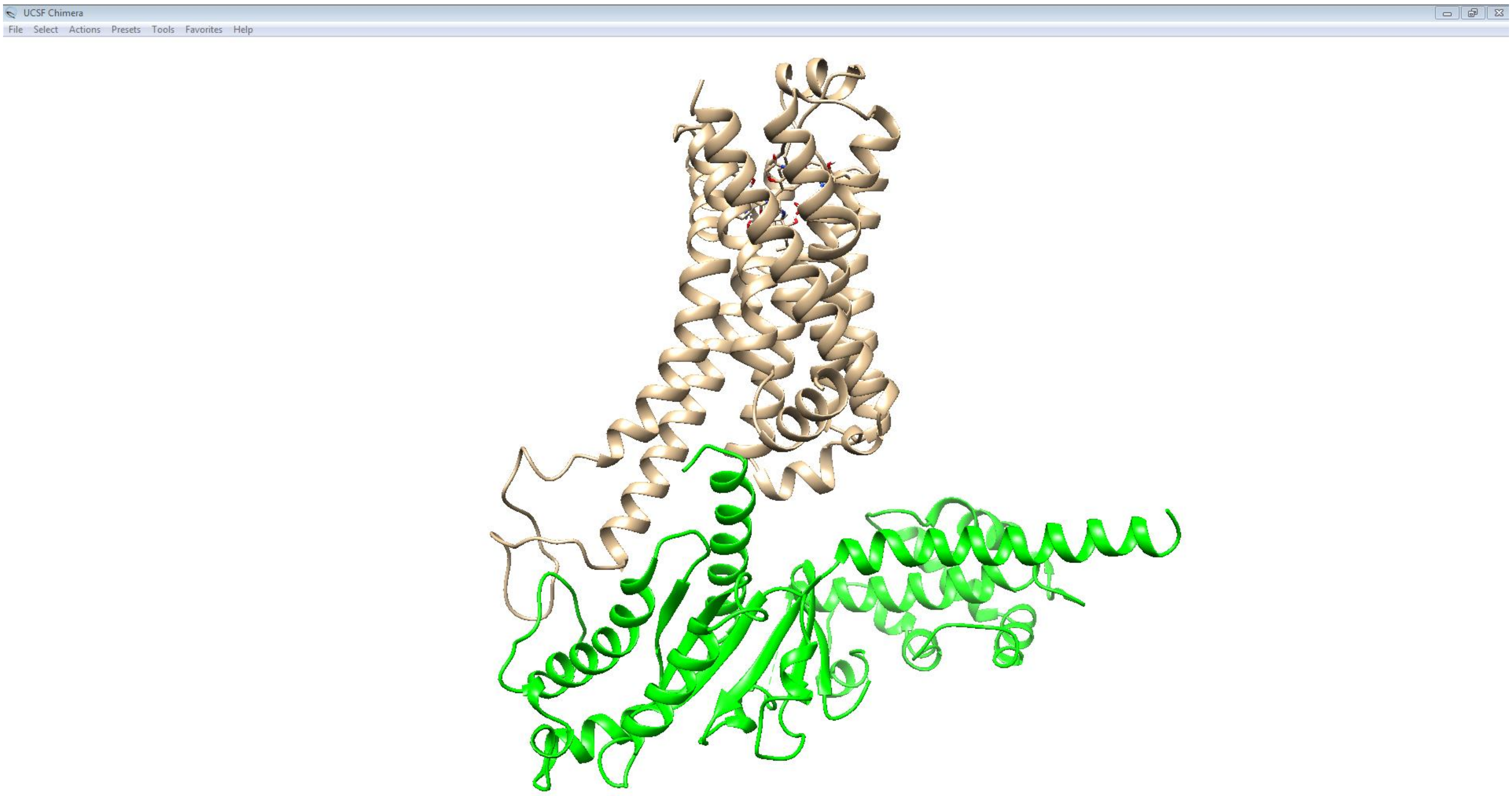

At this point we can compare the existing crystal structure of the liganded receptor-Gα structure (in this example we have used the β_2-adrenergic receptor with ligand BI-167107) with the solution from HEX 8.0.0 docking, and calculate the RMSD values using the Chimera program or the pyMOL program.

We open the crystal structure of the receptor with G-protein and the model from bioinformatic solution from HEX 8.0.0. together with Chimera program.

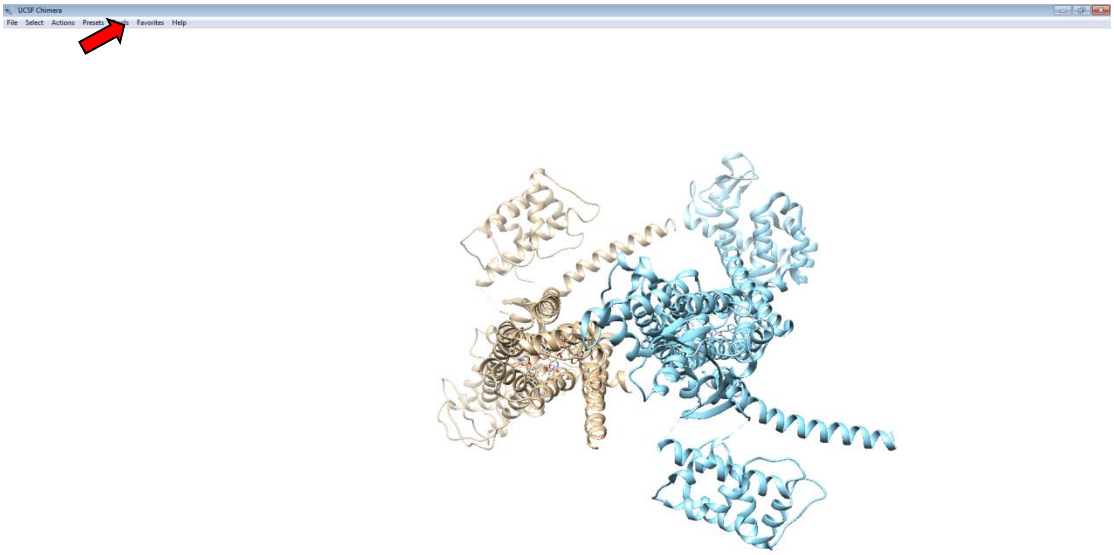

After that, we compare the two structures using the MatchMaker tool from Structure Comparison Tools.

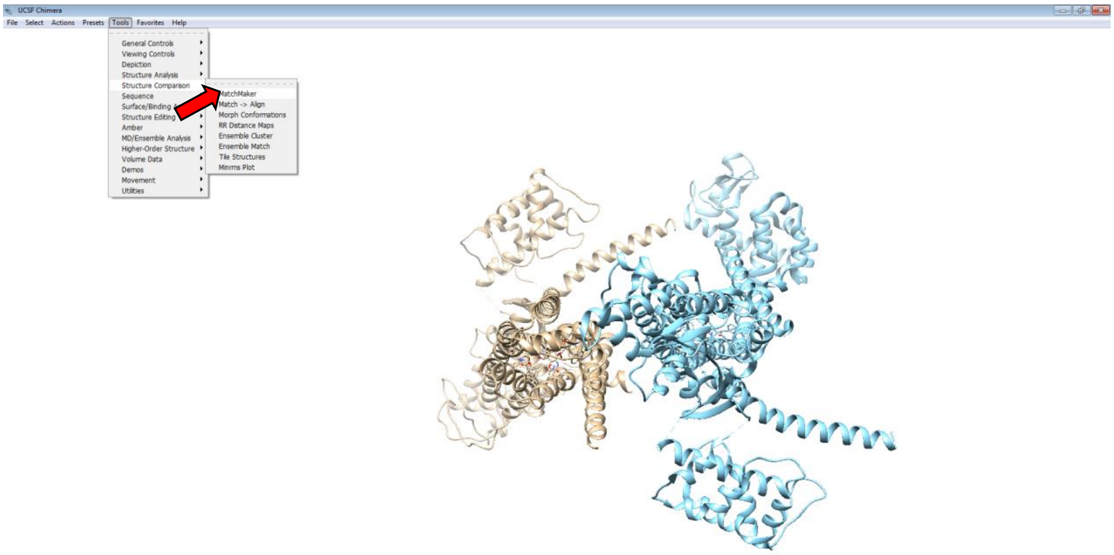

We select the first receptor as ‘’Reference structure’’ and the second receptor as ‘’Structure to match’’. Also, we select the option ‘’After superposition, compute structure-based multiple sequence aligment’’ and finally, we apply the MatchMaker.

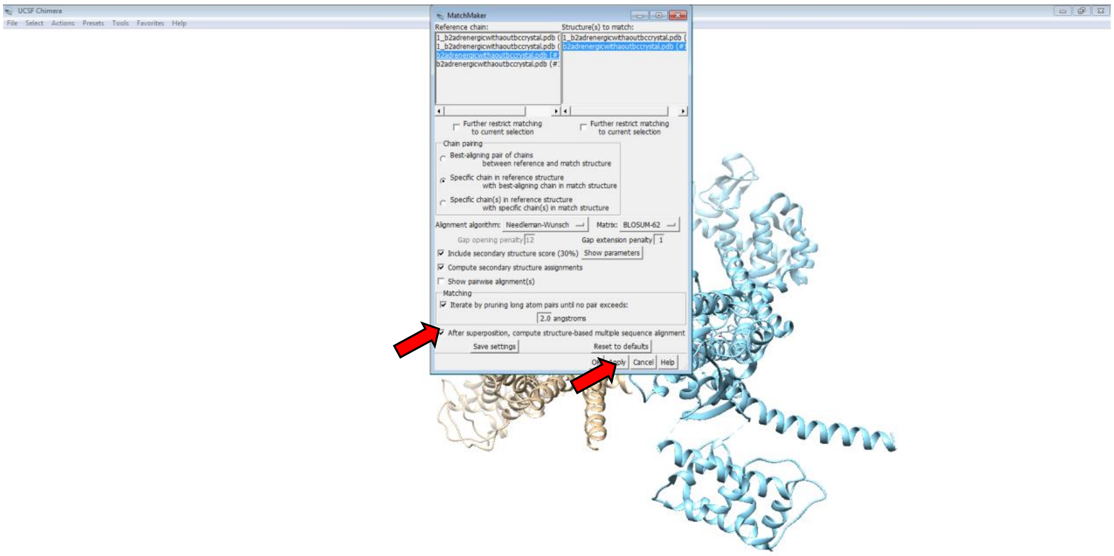

In the window that appears we select ‘’Apply’’, to display the receptor sequences.

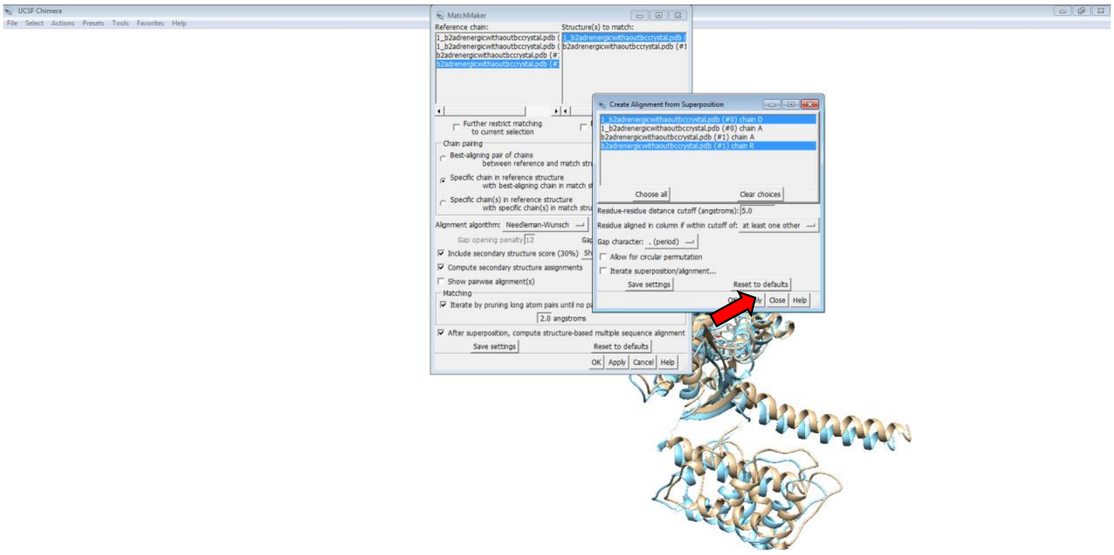

After that the window with the sequences of the receptors complexes being compared is displayed. We select all amino acids to calculate the RMSD (root-mean-square deviation of atomic positions) of each region.

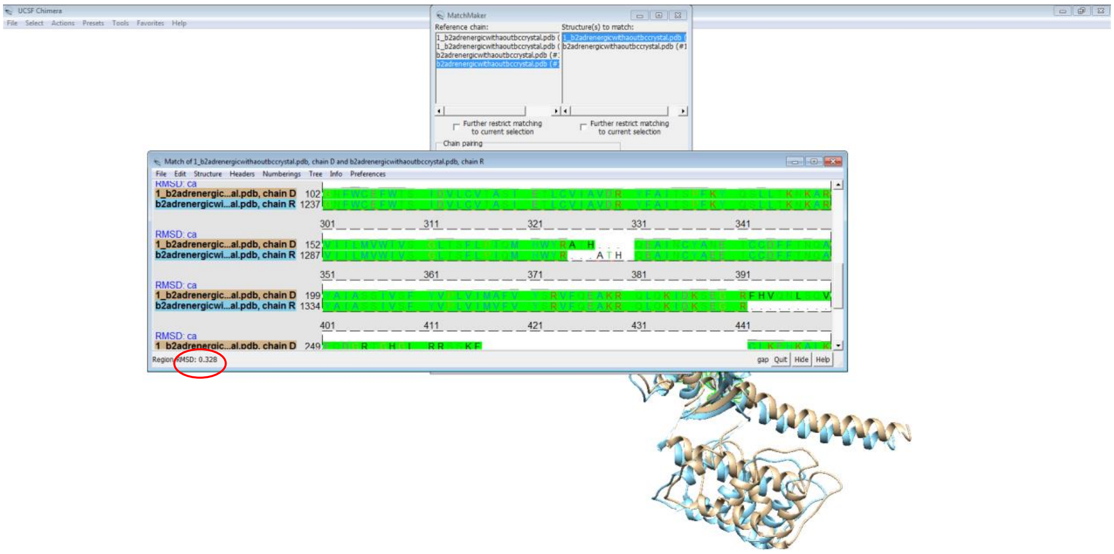

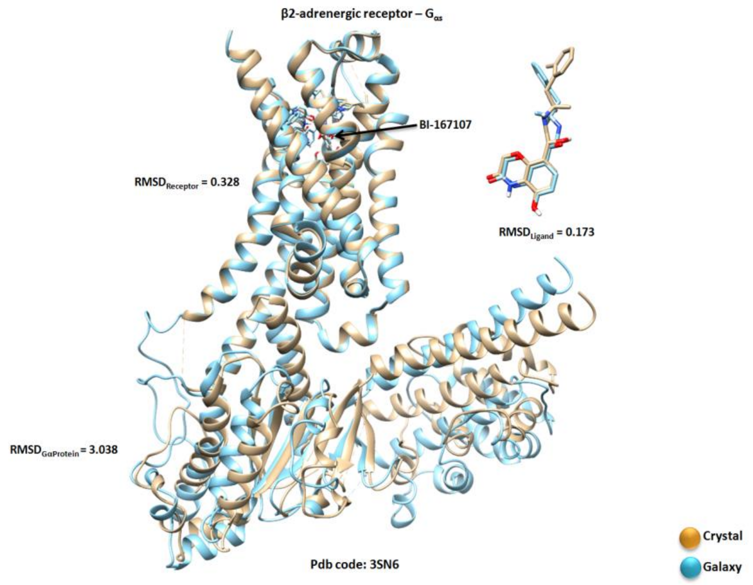

Comparison of crystal structures of receptors-G_{α} complexes, with the docking results computed with our approach. Data of the $G_{\alpha s}$-complexed β2-adrenergic receptor are presented. The crystal structure is shown in brown and docking model result in cyan. RMSD for the two molecules (in Å) are also given, together with the crystal structures used (pdb files). RMSD of the GPCR and the corresponding Gα proteins are presented. Measures are in Å.
